# Genetic and Cultural Reconstruction of the Migration of an Ancient Lineage

**DOI:** 10.1155/2015/651415

**Published:** 2015-09-30

**Authors:** Desmond D. Mascarenhas, Anupuma Raina, Christopher E. Aston, Dharambir K. Sanghera

**Affiliations:** ^1^Mayflower Organization for Research and Education, Sunnyvale, CA, USA; ^2^Department of Forensic Medicine and Toxicology, All India Institute of Medical Sciences, New Delhi 110029, India; ^3^Department of Pediatrics, University of Oklahoma Health Sciences Center, Oklahoma City, OK, USA

## Abstract

A rare R1a1 Y-haplogroup (Y-HG) L657 clade subtype designated as LPKSTR is found in most male members of a clan of “founder” families within the Goud Saraswat Brahmin community in Lotli town in Western India. TMRCA calculations using pairwise comparisons to control cohorts suggested a probable migration history distinct from the canonical narrative for medieval migration of orthodox Brahmin families to South India. Using Y-HG centroid analysis, chi-square analysis of TMRCA distributions and archeological find-spots, and discriminant function analysis we show that the parental Z93 L342.2 subclade in which LPKSTR occurs originated in West Asia and that LPKSTR individuals migrated toward the southeast by a Bolan Pass route distinct from the traditionally presumed route of Brahmin ingress into the Indian subcontinent. The proposed migration route is supported by archeological, toponymic, numismatic, linguistic, iconographic, and literary data. Lastly, we present cultural metrics demonstrating that these LPKSTR lineages retained distinct family practices with respect to literacy, religious practice, and emigration not shared with orthodox Brahmins of canonical geographic origin within the same community, despite centuries of intermarriage. Long-term transmission of differentiated family practices within a patrilineal endogamous community has rarely been documented.

## 1. Introduction

Increasingly granular subclade typing and the rapid growth of public databases have improved our ability to reconstruct the spread of R1a1 Y-haplogroup (Y-HG) branches along ancient trade and migration corridors in Asia. These new insights have established the dominant clade of R1a1 in the Iran-Afghanistan migration corridor to South Asia as carrying the defining SNPs Z93 and L342.2. In this group, the L657 branch is most abundant [1, 2].

In order to further dissect patrilineages within L657, unique microsatellite short tandem repeat (STR) profiles can be helpful. In this work we attempt to reconstruct a context for the migration of a culturally defined and highly endogamous group of priestly families carrying the rare haplotype “LPKSTR,” defined as R1a1 Y-HG individuals carrying the STR profile: DYS456 < 16, DYS458 > 15, and GATAH4 > 12. LPKSTR individuals listed in public databases also carry the SNPs Z93 and L342.2. The LPKSTR type is essentially absent from European R1a1 individuals, abundant in Afghanistan, and quite rare in L657 individuals from South Asia and the Arabian Peninsula.

Brahmin communities of South Asia have documented cultural and patrilineal antecedents (such as* gotra*) since ancient times [3–7]. Based in part on the ancient system of* gotra,* the prevalence of strictly endogamous patrilineages within Brahmin communities in South Asia simultaneously offers possibilities for studying horizontal transmission of distinct family practices over long intervals while also complicating the selection of geographic comparators traditionally employed in statistical methods. A Brahmin family Y-chromosome lineage is likely to exhibit greater genetic proximity to a culturally related group situated thousands of miles away than to any of the dozens of geographically proximate communities that might traditionally serve as comparators. Thus, in assessing genetic proximity within highly differentiated Brahmin milieu, the rational choice of selected comparators for TMRCA computation requires the use of historical data [8].

Ancient tradition places Saraswat Brahmins with the Sarasvata tribe on the banks of the Rig Vedic Sarasvati River, whose exact geographic location is not certain but is now believed by some scholars to be the Helmand-Arghandab basin in Afghanistan [8, 9], though this conclusion is still controversial. The region in question was settled prior to 4000 BCE and its ancient pre-Indo-European name was Haraxvaiti (later Harahuvati/Sarasvati) [10, 11]. In first millennium BCE, post-Achaemenid Sanskrit literature places Saraswat Brahmins in the northern Punjab, and a substantial Saraswat community remains there to this day. The other large Saraswat group in South Asia is the Goud Saraswat Brahmin (GSB) community, whose arrival in Goa's Salcete province during early medieval times is memorialized in local oral histories and legends variously claiming western (Gujarat-Rajasthan), central (Kannauj), or eastern (Tirhut) proximal origins. It is likely that today's Goan GSB community is the product of multiple migration events between the 7th and 11th centuries CE, each comprising families of claimed or actual tribal kinship with the original Sarasvata tribe.

Within the GSB Brahmin community, a high incidence of LPKSTR was anecdotally observed in male representatives of Pai (surname) families of a single* gotra* (exogamous lineage, Kaundinya) who are known founder families in a town communidade (Lotli). GSB are a subset of all Saraswats, who are a subset of all Brahmins.  Table 1(a) describes these identifiers. These so-called Lotli Pai Kaundinya (LPK) families may have retained distinct family practices, despite centuries of intermarriage with other GSB families of neighboring towns. In this work we explore the hypothesis that, unlike other Brahmins in the community, the LPK clan entered Goa via a western migration route from eastern Persia and Afghanistan, quite distinct from the traditional narrative for ingress of Vedic peoples into the subcontinent via a northern route through Gandhara and the Khyber Pass into the Punjab/Madhyadesa in the first millennium BCE and subsequently southward from there in medieval times. We compare genetic and cultural characteristics of LPK families with another clan within the same endogamous GSB community, with whom they have intermarried. Oral and written records of a unique form of town governance known as the “communidade” confirm each clan's identity and privilege in their respective towns for centuries [12–14]. Samples of Khatri (mercantile, Bolan Pass) and Saraswat Brahmin (priestly, Khyber Pass) communities are used as genetic comparators in this study because their cultural and migration histories represent the two alternatives we wish to compare.

Among modern migrants, social success and family practices prior to migration predict relative social success after migration [15]. Such observations point to portable, family-borne attitudes and capabilities including, for example, business and educational skills, or the pragmatism required for cultural adaptation. The matter has implications for other disciplines in the study of ancient history, for example, the “pots-are-not-people” maxim in archeology [16].

## 2. Materials and Methods

### 2.1. Samples and Subjects

The collection under informed consent of 35 male Saraswat Brahmin and 29 male Khatri DNA samples (listed in Table 2) has been described in previous publications [32–34]. 16 GSB samples (15 listed in Table 1(b); one non-R1a1 GSB individual (Q1a3 Y-haplogroup) was omitted from the analyses) were collected as follows: volunteers registered for cheek swab DNA analysis directly with Genebase, Inc. (Vancouver, BC), signed informed consent forms, and followed requisite procedures, as required by the Genebase Testing Laboratory. Each volunteer ordered Y-HG, subclade, and 67-STR tests. Volunteers were reimbursed for the cost of testing but received no other compensation. After testing was complete, volunteers provided investigators with access to their results on the Genebase database. Permission to use data for research and publication was provided in writing.

### 2.2. Ethics Statement

Studies were approved by each of the participating organizations' institutional committees in charge of oversight for ethical conduct in research. The collection and research use of Khatri DNA samples were approved by the Institutional Review Board of the University of Oklahoma Health Sciences Center, OK (IRB number 2911), and the Ethical Committee of Hero DMC Medical College and Heart Institute, Ludhiana, Punjab, India. The GSB study was approved by the Ethical Committee of the Mayflower Organization for Research and Education, Sunnyvale, CA. The collection and research use of Saraswat Brahmin DNA samples were approved by the Institute Ethical Clearance Body of the All India Institute of Medical Sciences, New Delhi.

### 2.3. DNA Analysis

Typical tests included the standard 67-STR, Y-backbone, and subclade tests. The details of these tests can be found on the Genebase website (http://www.genebase.com/). Assignment of Y-HG and subclade in this study used the tests for the markers and single nucleotide polymorphisms (SNPs) listed in Table S1 (see Supplementary Material available online at http://dx.doi.org/10.1155/2015/651415). The microsatellite short tandem repeats (STR) tested are listed in Table S2.

### 2.4. Validation of Lineages

Volunteers were identified and validated using oral communications with town social networks, communidade records, electoral rolls, church baptismal and marriage records going back 150–400 years for Christian subjects, and the Ramnathi temple community for Hindu participants. Brahmin status for Christian participants was confirmed from marriage records showing endogamy with other Brahmin families over several generations (Supplementary files S3, S4, S5, and S6).

### 2.5. TMRCA Computation

**Figure 1:**
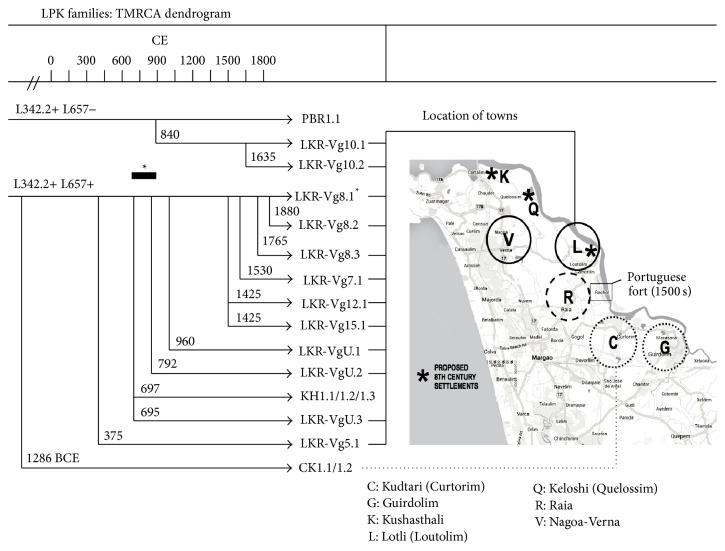
Dendrogram of LPK individuals. TMRCA branch-points for GSB cohort and controls. TMRCAs between pairs of L342.2+ individuals were computed using the modifications and validation steps for short time intervals described in Sections 1 and 2. Star symbol and dark horizontal bar indicate medieval Brahmin migration event. CE = common era; C = Kudtari; G = Guirdolim; K = Kushasthali; L = Lotli; Q = Keloshi; R = Rai; V = Nagoa-Verna.

**Figure 2:**
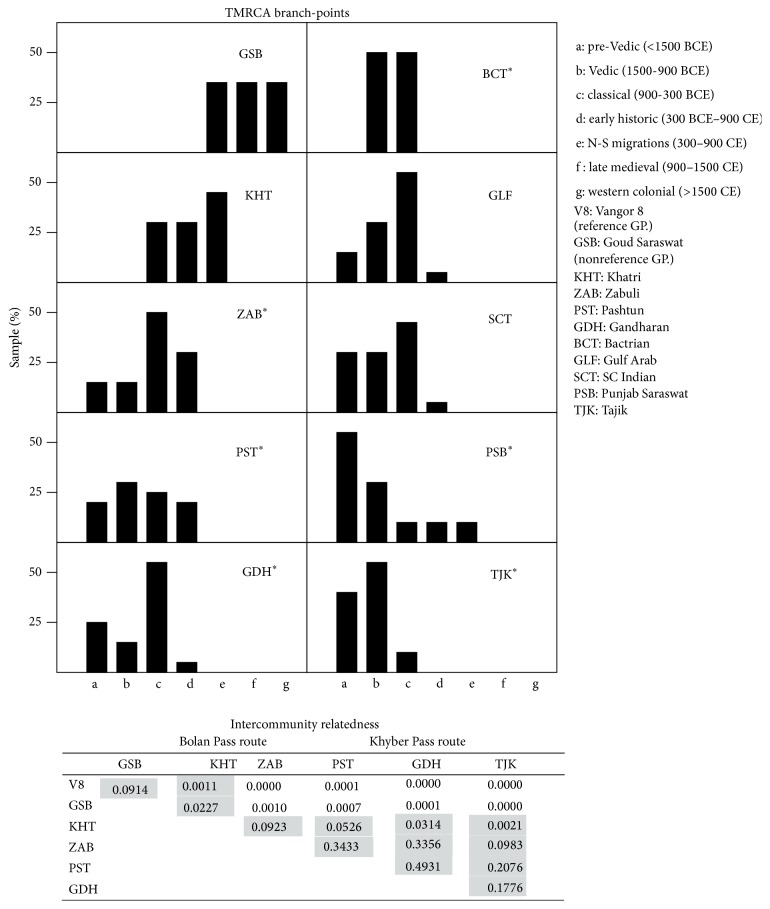
TMRCA branch-point histogram. Each bar represents a 600-year interval. For each community, pairwise TMRCA computations were performed between each R1a1 Y-HG individual and a reference modal STR profile of 3 LPK individuals of Vangor 8 (see Section 2). TMRCAs are plotted as a percentage of each population. Inset at top right shows *p* values (chi-square) for histogram differences between GSB and communities located near the Bolan and Khyber Passes. GSB = Goud Saraswat Brahmins; KHT = Khatri; ZAB = Zabulistan; PST = Pashtun; GDH = Gandhara; BCT = Bactria; GLF = Persian Gulf Countries; SCT = South, East, and Central Peninsular India; PSB = Punjabi Saraswat Brahmins; TJK = Tajiks; asterisks indicate that the comparison was made with a 17-STR profile (see Supplementary Files S3–S7).

The largest number of individual samples (three) available from a single LPK Vangor lineage was from Lotli Vangor 8 (V8) (Supplementary File S4 and Table 1(b)). Consequently, we selected V8 as a reference STR profile. 59 of 63 loci were identical in these three individuals plus another V8 individual not reported in this study. Modal values for the remaining loci were used in the reference profile. All V8 pairwise STR comparisons utilized only R1a1 Y-HG individuals (in the case of GSB, Khatri, Punjabi Saraswat, Gulf Arab, and Indian samples, all R1a1 individuals carried Z93 and L342.2 SNPs; individuals from the published cohorts were not tested for these SNPs). At least 40 common STRs were used for each comparison listed in Figure 1 and the dendrogram was constructed using the TMRCA values as branch-points. From church records between the late seventeenth century and the mid-twentieth century, when marriage and child-bearing cultural practices are believed to have remained stable, the mean generation time was 30 ± 2 years. In each pairwise TMRCA computation we input 28 and 32 years as generation times and averaged the values in each case. As this procedure yields proportional error values, these are not reported. Other variables in the formula (e.g., mutation rate of 0.025) were heuristically determined using 5 known branch-points of test subjects' ancestors from genealogical histories going back to ca. 1600 CE. For Figure 2, published data for the indicated cohorts allowed pairwise comparison of 17 or more STRs. Bidirectionally labile STRs were excluded from all comparisons. TMRCA computations used the following settings: average mutation rate 0.0025, >66% probability, and average generation of 28 years. These variables were selected based on five pairwise comparisons between GSB individuals of known TMRCAs between 50 and 400 years, based on baptismal and other historical records. By restricting comparisons to the same subclade, excluding bidirectionally labile STR markers and carefully calibrating formula settings with the aid of attested historical data, we expect our TMRCAs to be more accurate than those derived in traditional studies, thus increasing confidence that these computations may be accurate even over short time spans.

### 2.6. Public Databases

Y-haplogroup (Y-HG) data were downloaded from public databases at familytreedna.com in October 2013 (https://www.familytreedna.com/public/R1aY-Haplogroup and https://www.familytreedna.com/public/R1a). The latter link provides a useful diagram of R1a subclades, including the major Z93+ branches of Persian, Arabian, and South Asian provenance.

### 2.7. Data Availability Statement

All nonpersonally identifiable data used in the analyses is included either in the tables and figures of the main document and in supplementary files, cited published references, or in the public databases referenced. Individual STR profiles were deemed to be personally identifiable because the families of LPK Vangors are well known, and every male in each lineage would be expected to carry a nearly identical STR profile. For this reason, actual STR data are not disclosed, including those used for TMRCA calculations.

### 2.8. Toponymy, Numismatics, and Iconography

Toponymic stems were manually counted for each of the 20 major states in India using the database at https://www.facebook.com/whoyiz?fref=nf (viewed in September 2012) which lists all towns and villages for each state. Numismatic and iconographic data were collected online using the Google search engine or compiled from the published references cited. See Supplementary Files S17, S18, S19, and S20.

### 2.9. Gotra

Data on the gotras of Brahmin brides and grooms were collected and tabulated manually from two major matrimonial sites, http://www.jeevansathi.com/ (viewed in May 2012) and http://www.shaadi.com/ (viewed in January 2011). The data were quite similar. As the sample size was much larger from the first source, only those data are reported here (Supplementary File S25).

### 2.10. Church Records

In addition to the references cited, church records for Lotli town for the period 1914–1950 were obtained on microfilm from the Church of Latter Day Saints, Salt Lake City, UT. As these records are photographed from poorly stored, often water- or insect-damaged books handwritten by parish priests in Portuguese, not all records could be satisfactorily recovered or deciphered. Nevertheless, we estimate that at least 90% of all records were successfully tabulated (Supplementary File S5).

### 2.11. Statistical Methods

Probability values (*p* values) were computed using Student's *t*-test and expressed relative to indicated controls, except where otherwise noted. Group size was as noted in each case. For “fellow traveller” data, a discriminant function analysis was run to show that groups determined by genetic classification were significantly separate geographically based on distances to nearest point on the northern and southern migration routes. These same distances were then used in an *F*-test analysis of the geographic variation of each genetic group from each migration route testing the null hypothesis that it was not different from each route.

For centroid analysis, comparison of standard distances (square root of the average of the squared latitude and longitude differences from the centroid of the group) between pairs of populations used *F*-test analysis equivalent to testing for differences in variance between two groups. Linear regression was used to test for significant linear reduction in standard distances along the geographic line of migration (based on latitude and longitude of the centroids). Linear regression also was used to determine direction of migration through related centroids. The distributions of individuals across TMRCA determined categories were compared between populations using chi-squared tests of the null hypothesis that the distributions were equal. The chi-squared statistics from each of the pairwise population comparisons were then compared using *F*-tests to determine if one pair of populations was closer (i.e., more related) than another pair. Data analyses were conducted using Microsoft EXCEL 2013 and IBM SPSS Statistics (IBM Corp., released in 2011. IBM SPSS Statistics for Windows, Version 20.0. Armonk, NY; IBM Corp.). Results corresponding to *p* values lower than 5% are described as significant and reported.

## 3. Results

### 3.1. Validation of LPK Cohort and Comparison with Controls

16 male representatives of Brahmin founder families of ancient* vangors* in the towns of Lotli and Kudtari of Salcete province provided cheek swabs under informed consent. Their DNA was analyzed and compared with controls as described in Section 2 and their identifying characteristics are summarized in Table 1. All GSB samples (save for one Q1a3 individual, who was not studied further) were mapped to the Z93 L342.2 subclade, and 12 males were, additionally, L657. Ten individuals were of the LPKSTR haplotype, based on STR profile. Pairwise comparisons of STR profiles using 40–65 common loci per comparison were used to establish TMRCAs as described in Section 2. The Khatri and Kudtari control samples were compared as modal profiles. As the actual branch-points of five pairwise comparisons were known or inferred from baptismal and other records going back four centuries, it was possible to calibrate the TMRCA computation variables precisely, lending confidence to the remaining branch-point assignments. A dendrogram of these TMRCA computations was shown in Figure 1 (left panel, with town locations shown on the map in the right-hand panel).

40% of all branch-points between males of Lotli* vangors* (and between them and the Khatri controls) are clustered between 695 CE and 960 CE. This interval coincides with the peak period of medieval north-south Brahmin migrations [6]; southward expansion of maritime trade [35], Arab invasions of Sindh and Saurashtra in 712–738 CE (Supplementary File S43 and references), a documented 8th century migration of Chavda (“Chardo”) Gujjars to Goa [36], and upheavals and opportunities caused by the mid-eighth century transition from Chalukya to Rashtrakuta imperial rule provide additional backdrop to this historical period. Thus, these branch-points would seem to agree with folkloric accounts placing the initial GSB migration event to Salcete in 740 CE. The proximal origin of this migration was probably Saurashtra, based on the naming of their first new settlement (Kushasthali) after a famous ancient city in Saurashtra (modern Dwarka).

An additional set of three branch-points maps to the interval 1435–1530 CE, immediately preceding and following the arrival of Jesuit missionaries and the Portuguese Inquisition in 1560. Portuguese documents record the flight of some Hindu LPK, carrying with them the ancient idol of the town deity Ramnath [37]. LPK council seats (i.e.,* vangors*) vacated during the exodus may have been purchased and held by the other LPK of the original council. Two L657 control individuals (CK1.1, CK1.2) of families within the same* vangor* lineage in the neighboring town of Kudtari share a nearly identical STR profile (62/63 loci identity) and appear to be only distantly related to the LPK (TMRCA of ca. 3200 years, going back to early Vedic times). The families of Lotli and Kudtari claim a common membership in the GSB community, but Kudtari Brahmins may derive from a distinct migration event from Madhyadesa (Tirhut in Bihar, according to tradition).

We next examined the presumed kinship between the LPK samples and two communities in Punjab (see Section 1). Table 2 shows the results obtained from Y-HG and subclade analysis of 64 Khatri and Saraswat Brahmin males. Khatris, a mercantile community, are the traditional patrons of Saraswat priests. L657 Khatri individuals were the biggest subclade in this cohort, and 3 of 7 L657 individuals tested were LPKSTR. Unfortunately, the Saraswat Brahmin DNA samples available to us were insufficient for STR analysis in the present study, but a report of 17-STR profiles for Punjabi and Himachali Saraswat Brahmins has been published [32]. Surprisingly, LPKs appear more closely related to secular Khatri individuals than to Saraswat Brahmins. Khatris are historically associated with the ancient city of Multan, in southwestern Punjab, close to the Bolan Pass.

These results, taken together, suggest that the ancient LPK families who form the main subject of this study are a close-kinship clan of LPKSTR migrants, most likely from the northwest, and more closely related to Khatris than Punjabi Saraswats.

### 3.2. Route and Direction of Migration

We next examined genetic relatedness using Y-HG data from public databases and published studies.

#### 3.2.1. TMRCA Histograms

Figure 2 shows TMRCA branch-point histograms created using pairwise comparisons of R1a1 Y-HG individuals from various communities in the Persian Gulf-South Asia region as described in Section 2. Each histogram bar represents the percentage of individuals in each community whose TMRCA with the LPK reference (modal Vangor 8; see Methods) falls within an interval of 600 years. This methodology reveals the distribution of kinship to the reference sample in various geographic regions.

The inset table (Figure 2, top right) shows *p* values from TMRCA histogram comparisons between the LPK reference and five communities located in proximity either to the Bolan Pass (KHT, ZAB) or to Khyber Pass (PST, GDH) and one (TJK) further away on the Khyber route. Using a Bonferroni corrected threshold of *p* = 0.0011 for significance, shaded cells indicate pairs of communities that are not significantly different. As these populations are of similar size, the size of *p* values is consistent with the suggestion that the reference sample (V8) is more closely related to KHT and ZAB (Bolan Pass) than to PST, GDH, or TJK. One explanation of this result is that communities near the Bolan Pass are more closely related to LPK than Punjabi Sarawats.

#### 3.2.2. “Fellow Traveller” Footprints

**Figure 3:**
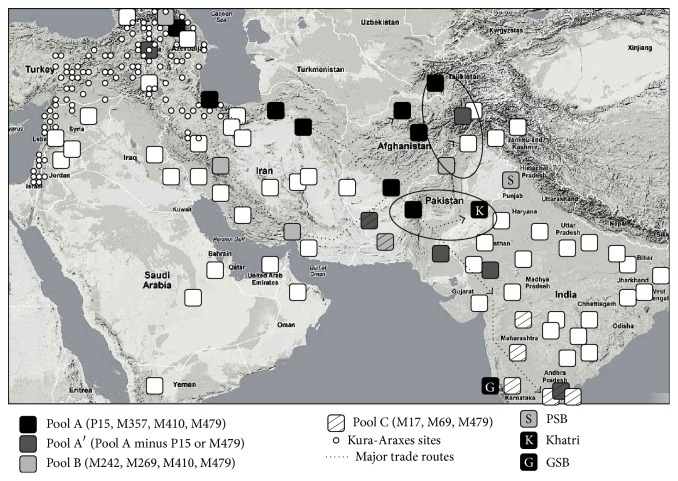
Genetic footprint of haplotypes. Coincident presence of SNPs above an arbitrary threshold of 2% (“fellow traveller”) was calculated from published genetic data [17–25]. See Section 2 for details. Major trade and migration routes deduced from archeological data are shown (dotted lines and arrows) as well as known archeological sites of the Kura-Araxes civilization (small circles) [26, 27]. Ovals indicate the areas adjacent to two major mountain passes used for genetic ingress into the subcontinent (Bolan: thick oval; Khyber: thin oval). K = Khatri; S = Saraswat Brahmin; G = GSB. Pool A (P15, M357, M410, and M479); Pool A′ (Pool A alleles minus either P15 or M479); Pool B (M242, M269, M410, and M479); Pool C (M17, M69, and M479); data from Supplementary File S11.

A previous study showed that the genetic makeup of communities to the north of the Dasht-i-Lut desert in Iran (“northern route”) is significantly different from the communities to its south (“southern route”). Moreover, the ingress of Anatolian genes was deemed to be greater than that from Pakistan, suggesting a directional flow from West Asia [38]. Based on published data on various communities and our analysis of the Punjabi cohort, we asked which non-R1a1 SNPs observed in the Punjabi samples are also found above an arbitrary incidence threshold of 2% in each of the other populations tested. This “fellow traveller” footprint (Figure 3) is shown for those communities for whom published data are available in the Transcaucasia-South Asia geographic corridor and adjacent regions [17–25]. Clusters of coincident SNPs appear to lie along ancient “northern” and “southern” Persian migration routes between Transcaucasia and the northwestern regions of South Asia, as suggested by the previous study [38]. Pools A and A′, which is a subset of A, initially follow a north Persian route and then spread southeast through Kandahar and the Bolan Pass into Sindh, Gujarat, and the Deccan. The Khatri cohort contains Pool A. Pool B, which is characteristic of Nakh communities of Hurro-Urartian lineage, such as Chechen and Ingush [39, 40] runs along the south Persian route into the Pakhtun belt and is represented in the Punjabi Saraswat Brahmin cohort. Note that these two putative migration routes cross at Kandahar, the suggested location of the ancient Sarasvata tribe. Pool C, which includes Dravidian language groups, partially overlaps the Pool A/A′ footprint, beginning in Baluchistan.

**Figure 4:**
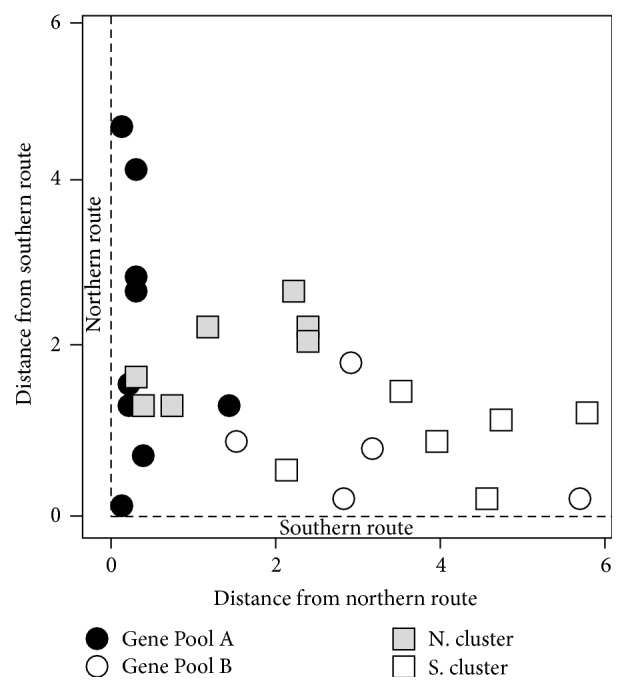
Discriminant function analysis. *F*-test analysis of the geographic variation of each fellow traveller genetic pool from each migration route showing distance (in arbitrary map units) between Pool A or Pool B and two major routes of migration: northern and southern Persian routes (see text). Pool A was not significantly distant geographically from the northern route (*p* = 0.50) but was significantly distant from the southern route (*p* < 0.0001) while Pool B was not significantly distant geographically from the southern route (*p* = 0.50) but was significantly distant from the northern route (*p* = 0.0051). This analysis confirms the association of Pool A with the northern Persian trail.

To establish whether Pool A and Pool B are associated with the northern and southern routes, respectively, we first used discriminant function analysis to show that groups determined by genetic classification were significantly separate geographically, at which they were *p* = 0.0009. *F*-test analysis of the geographic variation of each genetic pool from each migration route (Figure 4) showed that Pool A was not significantly distant geographically from the northern route (*p* = 0.50) but was significantly distant from the southern route (*p* < 0.0001) while Pool B was not significantly distant geographically from the southern route (*p* = 0.50) but was significantly distant from the northern route (*p* = 0.0051). This analysis confirms the association of Pool A with the northern Persian trail and its extension through Zabulistan into western India.

#### 3.2.3. Incidence of LPKSTR Haplotype

LPKSTR is absent from R1a1 clades other than Z93 clades, which are abundant in the corridor from Transcaucasia to South Asia on both sides of the Persian Gulf (Supplementary File S9). LPKSTR is almost entirely absent from Indian and Arab L657 individuals in the public databases. LPKSTR is found in eastern Transcaucasia and western Afghanistan. Table S7 shows incidence of the LPKSTR type within R1a1 Y-HG individuals for ten communities used in the TMRCA branch-point analysis (Figure 2). In addition, eastern Transcaucasians (Avars, Dargins, and Lezghins) show a high incidence of LPKSTR, unlike Nakh and Western Transcaucasian R1a1 cohorts suggesting provenance to the eastern side of Transcaucasia (Supplementary File S8). Regional communities within Iran are not well represented in this analysis, as their STR profiles are not publicly available. With that caveat, LPKSTR abundance appears to track the distribution of Pool A (Figure 2). Interestingly, the average incidence of LPKSTR among R1a1-HG individuals of the Khatri and Zabuli cohorts closest to the Bolan Pass (36.1%) is four times that of the Pashtun and Gandhara communities (9.1%) that live closer to the Khyber Pass, consistent with a history of LPKSTR migration along the so-called northern Persian trail into the subcontinent.

#### 3.2.4. Centroid Analysis

**Figure 5:**
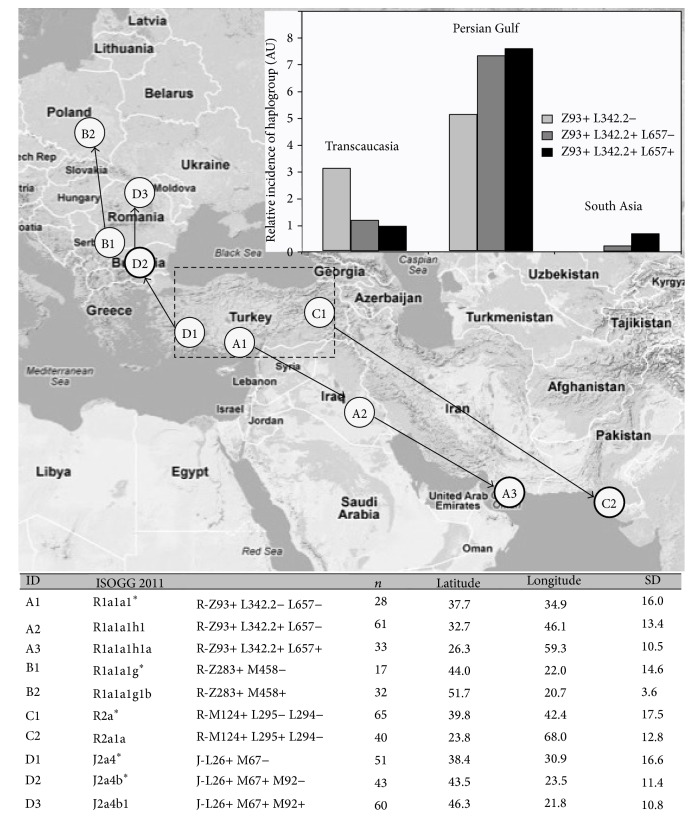
Net direction of migration deduced from centroid analysis. Centroids and average standard deviations were computed for several obligate allele series as described in Section 2. Each circle in the plot represents an allele. Thick-edged circles are alleles found in the Punjabi Khatri cohort. The bottom panel lists the series used in the computation. Inset: relative incidence of alleles (corrected for country sampling variance) in the public database, October 2012. Transcaucasia zone included Armenia, Georgia, Azerbaijan, and Turkey. Persian Gulf zone included all nations bordering the Persian Gulf, plus Afghanistan. South Asia zone included India and Pakistan. AU = arbitrary units (Supplementary Table S10).

Earlier published studies have primarily shown data from arbitrarily selected and sampled communities. To gain a less biased snapshot of haplotype distribution, we queried public databases and expressed relative incidence after correcting for unequal regional representation. In this way, it is possible to map virtual geographic centroids and standard distances (from the centroids) for obligate chronological allele series such as Z93 > Z93 L342.2 > Z93 L342.2 L657 and Z283 > Z283 M480. The prediction is that standard distance (SD) should* decrease* along a series and that the average position of centroids will reveal a direction of migration. This prediction was observed with SD for Z93 L342.2 L657 being significantly smaller than that for Z93 (*p* = 0.034) and Z93 L342.2 (*p* = 0.0006). The SD for Z283 M480 was less than that for Z283 (*p* < 0.0001). The SD for Z93 L342.2 L657 was numerically smaller than for Z93 L342.2 but did not reach significance (*p* = 0.068). Furthermore the Z93 group was significantly distant from the Z93 L342.2 group (lat. *p* = 0.0041; long. *p* = 0.032) and Z93 L342.2 from Z93 L342.2 L657 (lat. *p* = 0.0004, long. *p* = 0.0037) and a regression line fit to these groups has a direction just slightly south of southeast.  Figure 5 shows this for R1a1, R2a, and J2a4, all found in the Khatri cohort. A plot of the incidence of the series (inset in Figure 5) shows the more archaic alleles of the series preferentially in West Asia, with more recent alleles abundant in South Asia. The frequency of newer alleles is higher in the “Persian/Gulf” region, possibly suggesting a general location for their most significant expansion. This analysis supports the conclusion that Z93 and L342.2 expanded in a southeasterly direction from Transcaucasia into South Asia. By contrast, the series for the R1a1 Z283 M480 clade abundant in the Baltic and Slavic countries of Europe [1] proceeds north into Europe.

Finally, we plotted the mean-minus-mode values of the five nonconserved (i.e., “drifting”) STRs from the common set available for Transcaucasian (TC), Afghan (AF), and South Asian (IE) samples. Although this test does not establish the direction of migration, it suggests a gradient; that is, the Afghan sample shows intermediate values between Transcaucasian and Indian samples in all five cases (Supplementary File S12) consistent with an intermediate location for genetic drift.

#### 3.2.5. Archeological Data

A remarkable feature of the above genetic reconstructions is their compatibility with the archeological records of eastward expansion of West Asian populations in the 4th millennium BCE culminating in the so-called Kura-Araxes migrations of the post-Uruk IV period, with the advent of wheeled transport [26, 27, 41]. A ca. 3000 BCE model of a wheeled cart was recovered at Altyndepe [42]. New, possibly West Asian, body types are reported from the graves of Mehrgarh beginning in the Togau phase (3800 BCE) and this influx may have led to early pre-Harappan settlements of the Indus basin, Sothi-Siswal and Sorath regions between 3200 and 2800 BCE [43, 44]. Small circles in Figure 3 indicate the locations of known Kura-Araxes archeological sites [26, 27].

Several lines of evidence support the existence of a long-standing “Bolan Pass corridor” for genetic and cultural ingress from western Afghanistan into the subcontinent.

**Figure 6:**
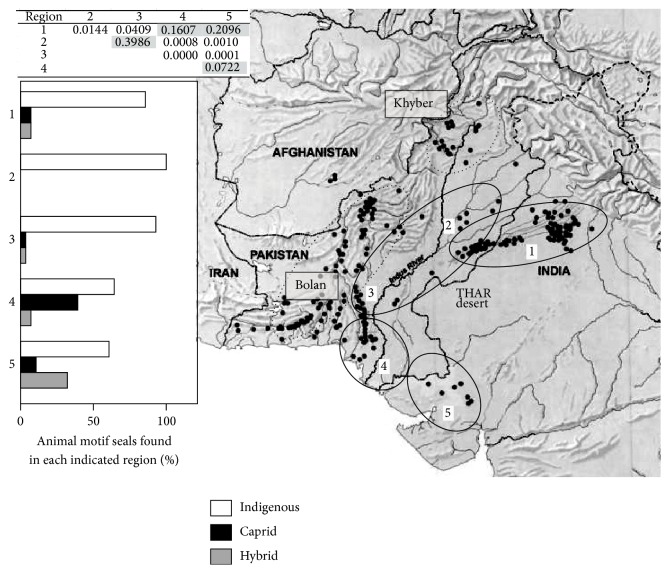
Distribution of find-spots for Harappan animal motif seals. Pre-Harappan archeological settlements, black dots (map adapted from [28]), are divided into 5 zones (ovals) for the purpose of scoring the incidence of animal seal icons (Supplementary Files S18, S22). Dotted ovals show the regions adjacent to the Bolan and Khyber Passes. Left top inset: *p* values (chi-square) from pairwise comparisons of regions. Left bottom inset: Elamite-Persian caprid iconography and indigenous (all other animal totems) and hybrid seals are scored as a percentage of all animal seals found in each of the five domains.

*(a) Harappan Iconography*.  Figure 6 shows the location of archeological sites in the pre-Harappan period (black dots), which strongly suggests a Bolan Pass route of entry radiating south to coastal Sindh and Sorath, as well as northeast to the Sothi-Siswal area. By contrast, the Gandharan (Khyber Pass route) appears to be much less significant, based on the number of settlements. An analysis of the iconography on Harappan animal seals (2600–1900 BCE) reveals a dominant “unicorn” type, widely distributed in the archeological record [45]. The provenance of minor animal totems, on the other hand, reveals regional differences. The caprid-ibex type (a well-known proto-Elamite Persian icon) and the bovid type (Sorath region) and 3-headed hybrids often seen in early 2nd millennium Dilmun seals from the Persian Gulf are more prevalent in the Bolan-Gujarat corridor. Seal totems are believed to be reliable indicators of tribal or religious cultural affinities. The distribution of chi-square *p* values from pairwise comparisons of seal find-spots in five Harappan regions (Figure 6, top left inset) suggests that the core Harappan domain represented by Mohenjodaro (region 3) and Harappa (region 2) shows similar cultural affinities, as represented in animal totems. Likewise southern Sindh (region 4) and Gujarat (region 5) show similarity, based on seal finds. But regions 4-5 are clearly distinct from 2 to 3, the core Harappan domain. These data may indicate the presence of a nonindigenous, proto-Elamite-Persian cultural influence along the region 4-5 corridor running through coastal Sindh into Gujarat. Additional support for a long-standing northwestern Indian cultural corridor is shown in Supplementary Files S41, S42.

**Figure 7:**
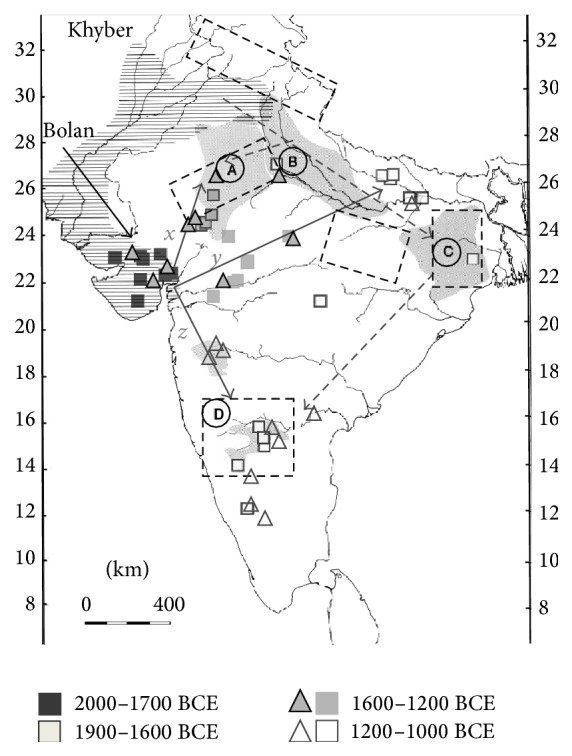
Second millennium BCE archeological footprint. Hypothetical migration (arrows) from Khyber and Bolan areas is shown. Horizontal hatched zone is the approximate extent of the Harappan civilization (2500–1900 BCE). Grey-shaded zones are the OCP-CH horizons A, B, C, and D (Supplementary File S14) [29, 30]. Hatched rectangles are the major areas of known copper ore deposits, associated with the OCP-CH cultures. Squares (WP-BRW find-spots; Supplementary File S13) and triangles (Persian-style channel-spouted bowl find-spots; Supplementary File S15) show temporal and spatial radiation of BRW migration tracks from the deduced Gujarat entry area [31].

*(b) 2nd Millennium Archeological Footprint*. The post-Harappan 2nd millennium BCE archeological record of the subcontinent (Figure 7) is dominated by two major traditions: the Ochre-Colored Pottery horizon, which is contiguous with the Copper Hoard tradition (OCP-CH) of the north and northeast [46, 47], and the Black-and-Red Ware (BRW) horizon which originates in Gujarat and covers much of the subcontinent. Based on provenance of the pottery, the BRW introgression through Gujarat is easiest to explain as originating in the northwest and entering the subcontinent through Sindh (not northern Punjab) in the second millennium BCE. This is especially seen in a subset of the BRW tradition, known as the White Painted BRW (WP-BRW) whose find-spots radiate from Gujarat in three directions, in a chronological series (square symbols in Figure 7). Coincident with these tracks is a separate (and slightly younger) archeological trail of “channel-spouted bowls” [28] believed to have Persian cultural origins (triangular symbols in Figure 7; see also Supplementary Files S15, S16).

#### 3.2.6. Medieval Migration

In the mid-eighth century CE, the heartland of Brahmin orthodoxy was located in the northeast of the subcontinent [5]. Qualitative evidence in support of the thesis that LPK families migrated to Salcete from a western, rather than eastern, proximal origin includes the following.

*(1) The Geographic Footprint of Clan Deities.* TMRCA data support a mid-eighth century migration of LPK families to Salcete, Goa (above). Such an event would be consistent with LPK devotional preferences: temples dedicated to their clan deity Ramnath are rare and still retain a distinctly western coastal footprint concentrated in the Saurashtra peninsula of Gujarat (Supplementary File S43). Another rare male clan deity brought to Salcete by Brahmin families other than LPK in the same migration event, according to tradition, is Manganath. Ancient locations for Manganath worship are almost exclusively located in the Saurashtra peninsula. Such striking colocation of minor religious traditions is unlikely to be coincidental, given the rarity of the epithets Ramnath and Manganath (rather than Rameshwar and Mangesh). Supplementary File S31 lists the male deity devotions prevalent in Salcete during the 16th century based on an inventory of Hindu idols in the >300 temples destroyed by the Portuguese Inquisition in 1567 [29]. This listing shows that Ramnath was worshipped as a primary deity only in Lotli town. For a summary of the emergent religious environment in early medieval Saurashtra, see Supplementary files S27, S28, and S29.

*(2) Arab Invasion.* We examined the possible impetus for southward migration in 740 CE. Supplementary File S43 shows the likely route for invading Arab Muslim armies into Gujarat while Al-Junayd was Emir of Sindh. These adventures include a major Arab raid in 725 CE (defeated by a Gurjar king in Malwa) and a final decisive defeat of Arab armies by Pulakesi at Navsari in 738-739 CE (Supplementary File S43 and references therein). Despite these results, Arab raids are reported to have devastated Vallabhi, the Maitraka capital, the most likely place of employment for literate Brahmins who lived in Saurashtra at the time.

Taken together, the above data support the noncanonical route of ingress proposed by our hypothesis. It should also be noted that genetic differences between LPK and Kudtari control individuals listed in Table 1 are consistent with migration from two different locations. (See also Supplementary Files S33–35, S41, and S42.)

### 3.3. Persistence of Family-Centric Practices within LPK Families

We investigated LPK family practices over recent centuries by tracking metrics that represent secular or pragmatic choices versus more orthodox alternatives. The underlying premise here is that the proposed LPK migration through a “non-Brahmanic” geographical zone influenced by Iranic cultures since ancient times and their genetic proximity to the financially successful (Supplementary File S40) Khatri community both suggest secular, possibly mercantile traditions for these families; by contrast, their counterparts in Kudtari may have migrated from orthodox Brahmin country in the northeast (see Section 1). If this assessment is accurate, one might expect a more secular tradition within LPK families compared with their Kudtari Brahmin neighbors. We therefore examined several metrics of orthodoxy.

**Figure 8:**
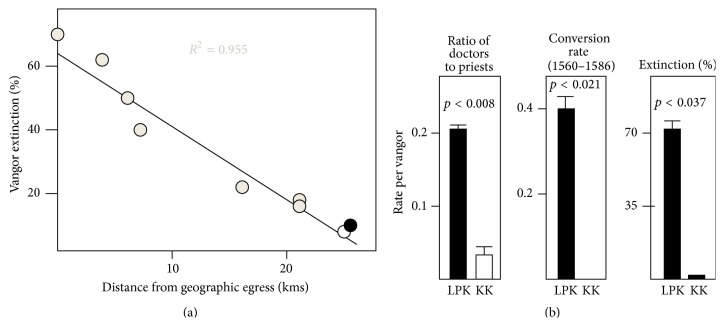
Emigration and orthodox practices. (a) Emigration rate, as deduced from vangor extinction (data from Supplementary File S39). See text for description. Each circle represents one Brahmin-founded town. White and black circles are Lotli and Kudtari towns, respectively. (b) Orthodox versus secular community practices (data from Supplementary Files S36, S38). See text for description. LPK vangors are compared with KK control vangors in the neighboring town, Kudtari.

*(a) Rate of Emigration*. Orthodox Brahmanic tradition discourages all forms of transoceanic travel.  Figure 8 shows extinction rates for LPK and Kudtari GSB (control)* vangors* over time, presumably because males of founder families left town permanently. In Figure 8(a), the cumulative extinction rates for all Brahmin communidade towns of Salcete are shown as of 1848. There is a clear and expected relationship between extinction rate and location relative to the ancient ingress-egress point for Salcete province, the town of Kushasthali (Portuguese: Cortalim). Lotli and Kudtari are equidistant and located furthest away, from this exit point. Thus the extinction rates for these two towns should not be affected by their location.  Figure 8(b) (right panel) shows that the extinction rate for LPK families has been significantly higher than that for Kudtari GSB (*p* = 0.037). The simplest explanation for these data is that the LPK community was historically more open to emigration suggesting, in turn, less orthodox family attitudes.

*(b) Career Choices*. We used family data from the eighteenth through early twentieth centuries to examine the rate at which male offspring joined the seminary [30] versus alternative, more secular way of achieving literacy that was available through the professions [48]. The data in Figure 8(b) (left panel) show that the LPK and Kudtari families emphasized different routes to literacy, with LPKs being more secular (higher ratio of medical school graduates versus ordained priests; *p* < 0.008).

*(c) Reluctance to Convert to Christianity in the Sixteenth Century*.  Figure 8(b) (middle panel) shows data from the history of early conversion to Christianity. Although both Lotli and Kudtari towns were located roughly equidistant from the Jesuit mission at Rachol, during its first three decades (between 1560 and 1586) virtually all Brahmin converts to Christianity originated from LPK families, with none from Kudtari families (*p* < 0.021). The greater reluctance to convert on the part of GSB families of Kudtari may be read as stronger (pre-Christian) religious orthodoxy.

*(d) Choice of First Names*. An additional supporting metric is shown in Supplemental File S32: in a census of first names of male children in the 16th century, Kudtari families were 3 times as likely as LPK families to select names associated with Shiva, who was by then of an older religious tradition, three centuries having elapsed since the conversion of most GSB from Shaivism to Vaishnavism.

*(e) Choice of Godparents at Conversion*. By emergent custom, the first Brahmin convert from each* vangor* in the sixteenth century took on the first and last names of the godparent, generally the baptizing Jesuit missionary. The names of all Jesuits missionaries posted in Goa during the sixteenth century are known from records kept at Society of Jesus headquarters in Rome. From this and other 16th century records we tentatively identified the godfather after whom each of the initial Brahmin converts was named. The choice of godfathers for early converts of the LPK families is informative: two of the first five converts, whose names are memorialized in a document dated May 31, 1586 [12], appear to have broken with tradition by taking godfathers who were not Jesuit missionaries but wealthy sea merchants instead (see Supplementary File 37). This extraordinary behavior may also indicate a strong secular attitude within the LPK families.

Taken together, the above data suggest a consistently more secular culture for the families of the LPK lineage compared to their more orthodox Kudtari neighbors, over a span of centuries. Nevertheless, both groups appear to have intermarried during this time and maintained their status as social elites in the same GSB priestly community for over a millennium.

## 4. Discussion

We have used statistical analysis and cultural data to construct a meta-narrative for the migration history of an endogamous LPK Brahmin lineage, largely comprising individuals of the rare type LPKSTR. This geographically restricted haplotype within the R1a1 Y-HG is found primarily in Eastern Persia/Western Afghanistan along an ancient migration corridor extending from greater Khurasan to the west coast of the Indian subcontinent. Multiple methodologies including centroid analysis, “fellow traveller” analysis, and TMRCA segmentation were implemented to examine the migration history of the LPK patrilineage. As L657 individuals in the Indian subcontinent rarely carry the LPKSTR signature (in early 2014 the largest public database did not contain a single example), it is possible that LPKSTR expansion in Afghanistan is linked to the late introgression of Saka, Parthian (Pahlava), or Hephthalite (Huna) tribes in a post-Iron-Age time frame. Such a hypothesis might also explain the high incidence of LPKSTR we found in some tribes of eastern Transcaucasia. The largest of these tribes, the Avars, is believed to have migrated there from Khurasan in the 6th century CE [49]. Perhaps this hypothesis can be reexamined when STR data for additional northern Persian communities is published.

Supplementary File S26 suggests that at least three different (large) pools of the R1a1 Y-HG entered the subcontinent separately over a span of at least four millennia. The ancestors of orthodox Brahmins may have entered the subcontinent in the Iron Age, while LPK individuals may entered with the most recent of those introgressions (first millennium CE). We supported a putative migration history for these lineages within archeological horizons backed by radiocarbon dating (Kura-Araxes, Quetta Ware, Harappan, OCP-CH, and WP-BRW). We further constructed a possible cultural context for LPK migration based on toponymic, iconographic, numismatic, epigraphic, architectural, and ancient literary data.

The earliest recognizable cultural zone along the LPKSTR migration path formed by the end of the fourth millennium BCE, with the advent of wheeled transport and a rapid influx of agricultural and metallurgical communities from Transcaucasia (the so-called Kura-Araxes migrations; Figure 3; [26, 27]). The archeological context for this cultural zone is the horizon comprising Tureng Tepe, Tepe Hissar, Altyndepe, Mundigak, Shahr-i-Sokhta, and Mehrgarh archeological sites from about 3000 BCE onward. This zone is associated with Burnished Grey Ware, Quetta Ware, and Faiz Mohammad Grey Ware and partially overlaps the Damb-Sadaat archeological horizon [50]. The mid-3rd millennium BCE Haraxvaiti urban complex (including Mundigak and Shahr-i-Sokhta, near present-day Kandahar and Seistan) rivaled Mesopotamian city-states in both size and sophistication [51, 52]. Southern reaches of this horizon included pre-Harappan settlements in Cholistan and Sothi-Siswal. If domesticated species were brought by these migrant agricultural communities, as is likely, founder effects may help explain the curious observation that house mice from Transcaucasia and North India are closely related by isozyme typing [53].

We show data in support of the proposition that coastal Sindh and Sorath (Gujarat) in Harappan times formed a distinct cultural corridor for Elamo-Persian caprid iconography, a proto-Iranic motif (Figure 6). After the collapse of the Harappan civilization, a large influx of settlers entering the subcontinent through Gujarat is memorialized in the BRW archeological tradition (Figure 7). Some of these settlers (marked by the so-called WP-BRW and “channel-spouted bowl” artifacts) may have been Iranic tribes. Mitra and Varuna toponyms are more prevalent near Gujarat and may date back to this period of settlement (Supplementary File S41), suggesting a geographic entry point for Vedic traditions that has not previously been suggested. Moreover, due to its geographic location, the Bolan Pass may have served as an important conduit for such migrations.

Some scholars have suggested that the Rig Veda was composed on the banks of a river in Haraxvaiti province in southern Afghanistan (Persian: Harahvati; Sanskrit: Sarasvati; possibly the Helmand or Arghandab) though their conclusion is considered controversial [8, 9]. The origin of the name “Rig” is unclear. It is noteworthy that the Kandahar region is adjacent to a large (now mostly desert) domain known locally as Rigestan (stan = place). The oldest “family books” of the Rig Veda fall into two categories, one group emphasizing text terms relating to the sacrificial tradition (Indra, Agni, and Soma; books 2, 3, 4, and 6) and the other group emphasizing the sun gods Mitra, Surya, and Vishnu (books 5 and 7) suggesting the apposition of at least two major distinct tribal affiliations in the early Vedic community (Supplementary File S21). Our findings are consistent with the speculative possibility that mainstream orthodox Brahmins (Indra, Agni, and Soma) and LPK lineages (Mitra) correspond to these two coextant traditions within the ancient Sarasvata tribe in Harahvaiti.

It is notable that Rig Vedic Book 7 is ascribed to the sage Vasishta, whose affiliation with the mainly Iranic sun-worshipping Mitra-Varuna tradition is nevertheless codified in the Hindu* gotra* system and became especially popular in the medieval assimilation of sun-worshipping Iranic peoples (Huna, Maitraka, and Gurjar) in Gujarat and the Western states [8]. LPK Brahmins follow the Kaundinya* gotra* of the Vasishta group. Moreover, Brahmins of the Vasishta* gotra* occur in significantly higher numbers along the western side of the subcontinent (Supplementary File S42). Taken together, these observations support the possibility of Mitra-Varuna antecedents for LPKSTR LPK families.

In early medieval times, LPKSTR LPK families probably lived in Saurashtra, Gujarat, at a time of great social mobility, assimilation of Iranic tribes, and devotional syntheses between Mitra sun worship and Pashupata Shaivism (such as Zun worship) (Supplementary Files S27–S30). One may speculate, therefore, that LPKSTR LPK families were exposed to several distinct religious traditions, in different locations over the centuries, a particularly significant matter for any priestly lineage and possibly related to their pragmatic, more secular outlook.

Within the GSB community, members of the oldest Smartha (Shaivite) sect have frequently accused the Madhva (worshippers of Vishnu, a sun god) of “allowing* western Brahmins* into the fold.” Based on our work, there may be some truth to their assertion. It is worth noting that the events in question would be about 1300 years old, which speaks to the astounding longevity of community memory.

One salient weakness of our work (as with most studies of ancient culture) is that only tentative cultural associations may be made with qualitative data types, especially for periods preceding historical times. On the other hand, we have here attempted to bring to the current analysis a cross-disciplinary approach rarely employed by other investigators of Y-HG. Some of the methodological innovations we have employed in this study may be of general use to future investigators of long genealogies.

## Supplementary Material

TABLE S1: Inclusion and exclusion SNPs used in haplotype assignment.
TABLE S2: Short tandem repeats (STRs) tested.
TABLE S3. Communidades of Salcete (1848): vangor listing.
TABLE S4. List of vangor families for Lotli, Rai and Kudtari towns.
TABLE S5. Baptismal records for Lotli town (1914-1932).
TABLE S6. Electoral rolls for KAMAT and PAI lineages (2013).
TABLE S7. TMRCA segmentation table.
TABLE S8. STRa incidence table.
TABLE S9. R1a1 subclades by geographic region.
TABLE S10. Centroid data table.
TABLE S11. Fellow traveller haplogroups: Transcaucasia to India.
FILE S12. STR drift: Transcaucasia to India.
TABLE S13. WP-BRW findspots.
TABLE S14. Copper hoard findspots.
TABLE S15. Channel-spouted bowl findspots.
TABLE S16. Votive tanks findspots.
TABLE S17. Toponym table.
TABLE S18. Harappan animal seal styles.
TABLE S19. Tribal coins: Northwest region.
TABLE S20. Indo-Persian borderlands coins.
TABLE S21. Sacred texts: word incidence.
TABLE S22. Harappan symbols table.
TABLE S23. Tripitaka census table.
TABLE S24. Listing of vibhedas (Skanda Purana).
TABLE S25. Gotra: incidence by community.
TABLE S26. Tribal gene introgression.
FILE S27. Religious traditions in medieval Saurashtra.
FILE S28. Attested Mihira clan locations (5^th^-8^th^ centuries CE).
TABLE S29. Varaha (Chalukya) style Aditya temples.
TABLE S30. Ramnath, Manganath, Siddhanath temples (Gujarat).
TABLE S31. Male deities in Salcete temples in 1567 (Portuguese Inquisition) .
TABLE S32. First names of 16^th^ century Lotli Brahmins.
TABLE S33. West-east gotra gradient.
TABLE S34. Bhaskara Maithili cohort gotras (7^th^ century).
TABLE S35. Orissa donee gotra listing (8-11^th^ centuries).
TABLE S36. Vangor conversion rate.
TABLE S37. Godfathers of converts prior to 1590.
TABLE S38. Ordination of catholic priests of Lotli town (18^th^-20^th^ century).
TABLE S39. Vangor extinction rate.
TABLE S40. Economic elites in South Asia.
FILE S41. Western cultural corridor: ancient period.
FILE S42. Western cultural corridor: Mitra-Varuna tradition (Kaundinya).
FILE S43. Saurashtran worship and Arab invasions (8^th^ century CE).

## Figures and Tables

**(a):** 

Identifier	Description
Town communidade (e.g., Lotli, Kudtari)	Ancient system of male hereditary town governance in which each of the original founder families of the town owns a share (vote) with land ownership rights and tax obligations

Vangor	A share (vote) in a communidade that is a patrilineal lineage, often using a single surname

Brahmins	Members of an ancient hereditary male priestly caste codified in the first millennium BCE

Saraswat Brahmins	The most ancient of all Brahmins, associated with the eponymous Sarasvati River and Sarasvata tribe

Goud Saraswat Brahmins (GSB)	An endogamous branch of the Saraswat Brahmin community formed by early medieval (8th–10th century CE) migration event(s) to Goa, in Western India

Gotra (e.g., Kaundinya)	Ancient Brahmin system of tracking patrilineage; gotra is exogamous; that is, a member must marry outside it; this system has been strictly observed for millennia

Kuldev/clan deity (e.g., Ramnath)	Ancient tradition in which a GSB clan maintains a primary deity at a single place of worship; this is a reliable characteristic for tracking family lineages

Lotli Pai Kaundinya (LPK)	GSB founder families of Lotli communidade of Kaundinya gotra, with the surname of “Pai”

**(b):** 

ID	Cohort	Y-HG clade	STRa	Source location	Vangor	Gotra	Ancient lineage
LKR-Vg5.1	GSB	L342.2+ L657+	No	Lotli (Loutolim)	Lotli 5	n.a.^*∗∗*^	Pai
LKR-Vg7.1	GSB	L342.2+ L657+	Yes	Lotli (Loutolim)	Lotli 7	n.a.^*∗∗*^	Pai
LKR-Vg8.1^*∗*^	GSB	L342.2+ L657+	Yes	Lotli (Loutolim)	Lotli 8	n.a.^*∗∗*^	Pai
LKR-Vg8.2^*∗*^	GSB	L342.2+ L657+	Yes	Lotli (Loutolim)	Lotli 8	n.a.^*∗∗*^	Pai
LKR-Vg8.3^*∗*^	GSB	L342.2+ L657+	Yes	Lotli (Loutolim)	Lotli 8	n.a.^*∗∗*^	Pai
LKR-Vg10.1	GSB	L342.2+ L657−	No	Lotli (Loutolim)	Lotli 10	n.a.^*∗∗*^	Pai
LKR-Vg10.2	GSB	L342.2+ L657−	No	Lotli (Loutolim)	Lotli 10	n.a.^*∗∗*^	Pai
LKR-Vg12.1	GSB	L342.2+ L657+	Yes	Lotli (Loutolim)	Lotli 12	n.a.^*∗∗*^	Pai
LKR-Vg15.1	GSB	L342.2+ L657+	Yes	Lotli (Loutolim)	Lotli 15	n.a.^*∗∗*^	Pai
LKR-VgU.1	GSB	L342.2+ L657+	No	Lotli (Loutolim)	Unknown	Kaundinya	Pai
LKR-VgU.2	GSB	L342.2+ L657+	Yes	Lotli (Loutolim)	Unknown	Kaundinya	Nayak
LKR-VgU.3	GSB	L342.2+ L657+	No	Lotli (Loutolim)	Unknown	Kaundinya	Pai
PBR1.1	GSB	L342.2+ L657−	No	Verem	Unknown	Kaundinya	Pai
CK1.1	GSB	L342.2+ L657+	No	Kudtari (Curtorim)	Kudtari 8	n.a.	Kamat
CK1.2	GSB	L342.2+ L657+	No	Kudtari (Curtorim)	Kudtari 8	n.a.	Kamat
KH1.1	Khatri	L342.2+ L657+	Yes	Punjab	None	n.a.	n.a.
KH1.2	Khatri	L342.2+ L657+	No	Punjab	None	n.a.	n.a.
KH1.3	Khatri	L342.2+ L657+	No	Punjab	None	n.a.	n.a.

^*∗*^Reference individuals for TMRCA computations; ^*∗∗*^Kaundinya gotra prior to conversion; n.a.: not applicable.

**(a):** 

ID	Haplogroup/subclade	Community
1139	L3 (M357+)	Khatri
159	G2a3 (P15+ S126+)	Khatri
173	**R1a1a1h1a (L342.2+ L657+)**	Khatri
20034	G2a3 (P15+ S126+)	Khatri
20062	J2b (M12+)	Khatri
20150	L3 (M357+)	Khatri
20180	J2a (M410+)	Khatri
214	**R1a1a1h1a (L342.2+ L657+)**	Khatri
216	J1 (M267+)	Khatri
30282	**R1a1a1h1a (L342.2+ L657+)**	Khatri
30809	**R1a1a1h1 (L342.2+ L657−)**	Khatri
30944	L3 (M357+)	Khatri
31311	L3 (M357+)	Khatri
40029	J2b (M12+)	Khatri
40036	R2 (M479+ M124−)	Khatri
40038	J1 (M267+)	Khatri
5002	**R1a1a1h1a (L342.2+ L657+)**	Khatri
5003	**R1a1a1h1a (L342.2+ L657+)**	Khatri
5005	R1a1	Khatri
5018	L1 (M76+)	Khatri
6008	J1 (M267+)	Khatri
6012	**R1a1a1h1a (L342.2+ L657+)**	Khatri
6024	R2 (M479+ M124−)	Khatri
6036	R1a1a1h1 (L342.2+ L657−)	Khatri
6040	R2a1a (L294+)	Khatri
6043	J2a4b (M67+)	Khatri
6230	R2 (M479+ M124−)	Khatri
934	L3 (M357+)	Khatri
940	L3 (M357+)	Khatri

**(b):** 

ID	Haplogroup/subclade	Community
HS21	**R1a1a1h1a (L342.2+ L657+)**	Saraswat
HS26	J2a4 (L26+)	Saraswat
HS28	L (M11+)	Saraswat
HS31	R2 (M479+)	Saraswat
HS32	L (M11+)	Saraswat
HS33	H1	Saraswat
HS34	R1a1	Saraswat
HS38	Q1a3 (M346+ M3−)	Saraswat
HS39	R1a1	Saraswat
HS4	R1a1a1h1 (L342.2+ L657−)	Saraswat
HS40	L (M11+)	Saraswat
HS44	**R1a1a1h1a (L342.2+ L657+)**	Saraswat
HS48	L (M11+)	Saraswat
HS51	L (M11+)	Saraswat
HS54	R1b1a2a1 (L150+)	Saraswat
PS10	Q1a3 (M346+ M3−)	Saraswat
PS15	**R1a1a1h1a (L342.2+ L657+)**	Saraswat
PS18	H1	Saraswat
PS22	R2 (M479+)	Saraswat
PS25	R1a1	Saraswat
PS29	**R1a1a1h1a (L342.2+ L657+)**	Saraswat
PS31	**R1a1a1h1a (L342.2+ L657+)**	Saraswat
PS32	R2 (M479+)	Saraswat
PS35	R2 (M479+)	Saraswat
PS36	L (M11+)	Saraswat
PS37	**R1a1a1h1a (L342.2+ L657+)**	Saraswat
PS47	H1	Saraswat
PS51	H1	Saraswat
PS53	R2 (M479+)	Saraswat
PS54	J (M304+)	Saraswat
PS57	R1a1	Saraswat
PS60	R1a1	Saraswat
PS7	J (M304+)	Saraswat
PS8	C	Saraswat
PS9	R1a1	Saraswat
