# Supplementary material for: Genetic and Cultural Reconstruction of the Migration of an Ancient Lineage
Source: Biomed Res Int. 2015 Sep 30;2015:651415. doi: 10.1155/2015/651415 (PMC4605215; doi:10.1155/2015/651415)
Supplement: Supplementary file 1 — TABLE S1: Inclusion and exclusion SNPs used in haplotype assignment. TABLE S2: Short tandem repeats (STRs) tested. TABLE S3. Communidades of Salcete (1848): vangor listing. TABLE S4. List of vangor families for Lotli, Rai and Kudtari towns. TABLE S5. Baptismal records for Lotli town (1914-1932). TABLE S6. Electoral rolls for KAMAT and PAI lineages (2013). TABLE S7. TMRCA segmentation table. TABLE S8. STRa incidence table. TABLE S9. R1a1 subclades by geographic region. TABLE S10. Centroid data table. TABLE S11. Fellow traveller haplogroups: Transcaucasia to India. FILE S12. STR drift: Transcaucasia to India. TABLE S13. WP-BRW findspots. TABLE S14. Copper hoard findspots. TABLE S15. Channel-spouted bowl findspots. TABLE S16. Votive tanks findspots. TABLE S17. Toponym table. TABLE S18. Harappan animal seal styles. TABLE S19. Tribal coins: Northwest region. TABLE S20. Indo-Persian borderlands coins. TABLE S21. Sacred texts: word incidence. TABLE S22. Harappan symbols table. TABLE S23. Tripitaka census table. TABLE S24. Listing of vibhedas (Skanda Purana). TABLE S25. Gotra: incidence by community. TABLE S26. Tribal gene introgression. FILE S27. Religious traditions in medieval Saurashtra. FILE S28. Attested Mihira clan locations (5th-8th centuries CE). TABLE S29. Varaha (Chalukya) style Aditya temples. TABLE S30. Ramnath, Manganath, Siddhanath temples (Gujarat). TABLE S31. Male deities in Salcete temples in 1567 (Portuguese Inquisition) . TABLE S32. First names of 16th century Lotli Brahmins. TABLE S33. West-east gotra gradient. TABLE S34. Bhaskara Maithili cohort gotras (7th century). TABLE S35. Orissa donee gotra listing (8-11th centuries). TABLE S36. Vangor conversion rate. TABLE S37. Godfathers of converts prior to 1590. TABLE S38. Ordination of catholic priests of Lotli town (18th-20th century). TABLE S39. Vangor extinction rate. TABLE S40. Economic elites in South Asia. FILE S41. Western cultural corridor: ancient period. FILE S42. Western cultural corridor: Mitra- [file 651415.f1.pdf]

Supplemental Files

TABLE S1: Inclusion and exclusion SNPs used in haplotype assignment.
TABLE S2: Short tandem repeats (STRs) tested
TABLE S3. Communidades of Salcete (1848): vangor listing
TABLE S4. List of vangor families for Lotli, Rai and Kudtari towns
TABLE S5. Baptismal records for Lotli town (1914-1932)
TABLE S6. Electoral rolls for KAMAT and PAI lineages (2013)
TABLE S7. TMRCA segmentation table
TABLE S8. STRa incidence table
TABLE S9. R1a1 subclades by geographic region
TABLE S10. Centroid data table
TABLE S11. Fellow traveller haplogroups: Transcaucasia to India
FILE S12. STR drift: Transcaucasia to India
TABLE S13. WP-BRW findspots
TABLE S14. Copper hoard findspots
TABLE S15. Channel-spouted bowl findspots
TABLE S16. Votive tanks findspots
TABLE S17. Toponym table
TABLE S18. Harappan animal seal styles
TABLE S19. Tribal coins: Northwest region
TABLE S20. Indo-Persian borderlands coins
TABLE S21. Sacred texts: word incidence
TABLE S22. Harappan symbols table
TABLE S23. Tripitaka census table
TABLE S24. Listing of vibhedas (Skanda Purana)
TABLE S25. Gotra: incidence by community
TABLE S26. Tribal gene introgression
FILE S27. Religious traditions in medieval Saurashtra.
FILE S28. Attested Mihira clan locations (5th-8th centuries CE).
TABLE S29. Varaha (Chalukya) style Aditya temples
TABLE S30. Ramnath, Manganath, Siddhanath temples (Gujarat)
TABLE S31. Male deities in Salcete temples in 1567 (Portuguese Inquisition)
TABLE S32. First names of 16th century Lotli Brahmins
TABLE S33. West-east gotra gradient
TABLE S34. Bhaskara Maithili cohort gotras (7th century)
TABLE S35. Orissa donee gotra listing (8-11th centuries)
TABLE S36. Vangor conversion rate
TABLE S37. Godfathers of converts prior to 1590
TABLE S38. Ordination of catholic priests of Lotli town (18th-20th century)
TABLE S39. Vangor extinction rate
TABLE S40. Economic elites in South Asia
FILE S41. Western cultural corridor: ancient period
FILE S42. Western cultural corridor: Mitra-Varuna tradition (Kaundinya)
FILE S43. Saurashtran worship and Arab invasions (8th century CE).

TABLE S1: Inclusion (shaded) and exclusion SNPs used in haplotype assignment.

Backbone SNPs: (highlighted SNPs are for R1a1 Y-haplogroup)

L62 A > G	M304 A > C
M11 A > G	M343 C > A
M122 T > C	M45 G > A
M130 C > T	M479 C > T C Negative
M168 C > T	M60 T ins
M170 A > C	M69 T > C
M173 A > C	M70 A > C
M174 T > C	M89 C > T
M175 TTCTC del	M9 C > G
M201 G > T	M91 T del
M207 A > G	M96 G > C
M214 T > C	P256 G > A
M230 T > A	SRY10831.1 A > G
M231 G > A	SRY10831.2A
M242 C > T	

R1a1a1h1a subclade:

L175 CTGTdel	M434 G > A
L260 G > T	M458 A > G
L342.2 A > G	M56 A > T
L365 T > C	M87 T > C
L366 TAACdel	P278.2 G > A
L657 G > A	P98 C > T
M157.1 A > C	PK5 C > T
M198 C > T	Z280 C > T
M334 C > A	Z93 G > A
M417 G > A	

J2a4b subclade:

M12 G	M47 G
M172 T > G	M62 T
M205 T	M67 A > T
M241 G	M68 A
M267 T > G	M99 No A del
M280 G	P279 G
M319 T	P56 A
M321 C	P58 T
M339 T	P81 C
M340 G	P84 C
M365 A	L24 G
M390 No A Ins	L26 T > C
M410 A > G	L136 No T del
M419 No AAAAG del	

Other subclades:

M124 C > T	M274 C
L295 G > A	M317 No GA del
L294 A > T	M349 G
M20 G	M357 A
M76 T	PK3 T

TABLE S2: Short tandem repeats (STRs) tested*.

DYS 19	DYS 385a	DYS 385b	DYS 388	DYS 389i	DYS 389ii	DYS 390	DYS 391	DYS 392	DYS 393
DYS 413a	DYS 413b	DYS 426	DYS 436	DYS 437	DYS 438	DYS 439	DYS 442	DYS 444	DYS 445
DYS 446	DYS 447	DYS 448	DYS 449	DYS 450	DYS 452	DYS 453	DYS 454	DYS 455	DYS 456
DYS 458	DYS 459a	DYS 459b	DYS 460	DYS 461	DYS 462	DYS 463	DYS 464a	DYS 464b	DYS 464c
DYS 468	DYS 472	DYS 481	DYS 485	DYS 492	DYS 511	DYS 518	DYS 520	DYS 522	DYS 527a
DYS 527b	DYS 531	DYS 534	DYS 537	DYS 540	DYS 557	DYS 565	DYS 570	DYS 568	DYS 576
DYS 578	DYS 588	DYS 590	DYS 607	DYS 612	DYS 614	DYS 640	DYS 641	DYS 644	DYS710
DYS 711	DYS 724a	DYS 724b	GATA A10	GATA C4	GATA H4	YCA IIa	YCA IIb		

* Not all samples were tested for all STRs.

Table S3. **Communidades of Salcete (1848): Vangors** [from ref 1]. Subject towns of this study are highlighted.

Town / Village	Original	Extinct	Brahmin	Chardo	Other
Aquem	3	0	2	1	0
Benaulim	12	2	10	0	0
Betalbatim	5	0	0	5	0
Calata	5	0	0	5	0
Camorlim	8	0	0	8	0
Cansaulim	10	8	?	?	?
Canna	3	0	0	3	0
Carmona	10	0	0	10	0
Cavelossim	10	0	0	0	10
Cavorim	4	1	0	3	0
Chandor	4	1	0	3	0
Chicalim	2	0	2	0	0
Chicolna	3	2	0	1	0
Chinchinim	8	0	0	7	1
Coelim	6	0	0	6	0
Collua	5	3	0	3	0
Cortalim	24	17	6	1	0
**Curtorim (Kudtari)**	**20**	**1**	**19**	**0**	**0**
Dabolim	2	2	0	0	0
Dramapur	5	0	1	0	4
Davorlim	15	0	0	1	14
Deussua	10	1	0	4	5
Dicarpale	19	12	0	0	7
Doncolim	3	0	0	3	0
Gandaulim	3	0	0	3	0
Gonsua	3	0	0	3	0
Guirdolim	4	1	3	0	0
Hissorim	3	1	0	1	1
**Loutolim (Lotli)**	**26**	**23**	**23**	**0**	**0**
Macazana	3	0	3	0	0
Majorda	10	0	0	10	0
Margao	28	6	22	0	0
Mormugao	3	0	0	3	0
Nagoa	14	7	7	0	0
Orlim	5	0	0	5	0
Palle	5	0	0	5	0
Quelossim	8	5	3	0	0
**Raia (Rai)**	**14**	**2**	**12**	**0**	**0**
Sancoale	14	0	14	0	0

Sarzora	4	0	0	4	0
Seraulim	6	2	0	4	0
Sernabatim	5	2	0	3	0
Sirlim	7	0	0	0	7
Telaulim	12	0	0	0	12
Vaddem	5	0	5	0	0
Vanelim	4	2	0	2	0
Varca	10	0	0	10	0
Velcao	6	0	0	6	0
Verna	48	19	29	0	0
Utorda	4	0	0	4	0

References:

1. Xavier, Filipe Nery (1848) *Communidades de Salcete*. Bosquejo Historico.

Table S4. **List of vangor families for Lotli, Rai and Kudtari towns**

Town: Lotli (Loutolim)

[Source: Primeiro Cristaos em Salcete. Oriente Portuguez

VANGOD	Ancestral Name	Post-Conversion	Name of Early Convert
1	KINI	LOBO	
2	PAI	COSTA	Antonio da Costa
3	SINAI?	GOMES	
3		MIRANDA	
4	SINAI?	MENEZES	
5	PAI	VALADARES	Joao de Valadares
5		VAZ	
6	NAYAK	MENDANHA	
6	NAYAK	SILVA	
7	PAI	SA	Pantaleao de Sa
8	PAI	MASCARENHAS	Jeronimo Mascarenhas
8		CARVALHO	
9	NAYAK?	ALVES	
9		BAPTISTA	
9		CRUZ	
9		CARDOSO	
9		NORONHA	
10	PAI	COLACO (extinct)	Pedro Colaco
11	NAYAK	(extinct)	
12	PAI	ARAUJO	
12	PAI	BARRETO	
12	PAI	MAGALHAES	
13	NAYAK?	BRITO	
13		CRUZ	
13		FARIA	
13		QUADROS	
14	NAYAK?	REGO	
15	PAI?	MONTEIRO	

Note: 11 vangors added by merger with adjacent village of Racaim in 1627 were excluded from this list.

Town: Rai (Raia)

[Source: Primeiro Cristaos em Salcete. Oriente Portuguez

VANGOD	Ancestral	Post-Conversion	Name of Early Convert

1	PAI	ESTEVAO	Thomas Estevao
1	PAI	ALBUQUERQUE	Mathias de Albuquerque
2	KAMAT	VIEGAS	Amador Viegas
2	KAMAT	PEREIRA	Goncalo Pereira
3	PAI	QUADROS	Antonio de Quadros
3	PAI	LIMA (LEMOS)	Francisco de Lima
4?	SINAI	extinct?	
5?	PAI	PARRAS	Pedro Parras
5?	PAI	PEREIRA	Bernardo Pereira
5?	PAI	GONCALVES	Manuel Goncalves
5?	PAI	BARBOSA	Sebastiao Barbosa
5?	PAI	COUTINHO	Antonio Coutinho
5?	PAI	RANGEL	Joao Rangel
6?	NAYAK	DIAS?	
7?	MALO	extinct?	
8?	PAI	??	
9	NAYAK	MENEZES	Lourenco de Menezes
9	NAYAK	FERNANDES	Agostinho Fernandes
10	KAMAT	SOUZA	Joao de Souza
10	KAMAT	VALERIANO	Aleixo Valeriano
11	PAI	MONIZ	Antonio Moniz
12	KAMAT	FALEIRO	Manuel Faleiro
13	PAI	MENDONCA?	

Town: Kudtari (Curtorim)

VANGOD	Ancestral	Post-Conversion	Name of Early Convert
1	KAMAT	MENEZES	Aleixo de Menezes
1	KAMAT	BORGES	Joao Borges
2	PAI	XAVIER	
2	PAI	COSTA	
3	KAMAT	VAZ	
4	KAMAT	FERNANDES	Brizio Fernandes
4	KAMAT	GOMINDES	Aleixo Gomindes

5	KAMAT	DINIZ	Agostinho Diniz
6	PAI	COELHO	
6	PAI	MENEZES	Alexandre de Menezes
7	KAMAT	COSTA	
8	KAMAT	PIMENTA	
8	KAMAT	COSTA	Lourenco da Costa
9	KAMAT	ESTIBEIRO	
10	KAMAT	SARDINHA	
10	KAMAT	CRUZ	
11	NAYAK	LUIS	Francisco Luis
12	KAMAT	BARRETO	Francisco Barreto
12	KAMAT	FERNANDES	Brizio Fernandes
12	KAMAT	ANDRADE	
13	PAI	RODRIGUES	Goncalo Rodrigues
14	KAMAT	VEIGA	Manuel de Veiga
15	KAMAT	PAES	
16	KAMAT	COSTA	Antonio da Costa
16	KAMAT	VIEGAS	
17	KAMAT	COSTA	Antonio da Costa
18	PAI	BRITO	Antonio de Brito
19	UNKNOWN	LOURENCO	

Table S5. **Baptismal records for Lotli town (1914-1932).** Handwritten Portuguese baptismal records for founder LPK families for the period 1914 through 1932+ were obtained from the Lotli parish church (Salvador do Mundo), translated and transcribed.

Family	Birth	Baptism	Child	Father
MONTEIRO	14-Apr-14	14-Apr-14	Isidoro Francisco Aleixo Manoel Monteiro	Rosario Mariano Alleluia Monteiro
ARAUJO	20-Jan-14	15-Apr-14	Maria Luzia Araujo	Joao Caetano Sebastiao Eduardo A
MASCARENHAS	5-May-14	14-May-14	Maria Carolina Alda de Araujo Mascarenhas	Joaquim Lourenco de Araujo Masca
MONTEIRO	7-Nov-14	22-Nov-14	Agostinho Francisco Esmeraldo Monteiro	Joao Cosme Damiao Bossuet Mont
DE SA	22-Nov-14	2-Dec-14	Maria Podenciana Quiteria do Rosario e Sa	Jeronimo Gabriel de Sa
VALADARES	31-Jan-15	21-Feb-15	Piedade Sofia Valadares	Joaquim Sebastiao Valadares
DE SA	23-Mar-15	30-Mar-15	Lourenco Manuel de Sa	Antonio de Sa
MONTEIRO	16-Apr-15	25-Apr-15	Francisco Agostinho Gracias Monteiro	Cesar Baronio da Conceicao Monte
ARAUJO	18-Apr-15	25-Apr-15	Dominginho Anselmo de Araujo	Jose Pascoal Araujo
MONTEIRO	12-Apr-15	9-May-15	Julio Cesar Francisco Monteiro	Joaquim Heliodoro Bernadino Mont
ARAUJO	18-Jul-15	25-Jul-15	Joao Vicente Francisco Camilo Teles Araujo	Joaquim Mariano Hilario dos Milagr
MONTEIRO	26-Apr-15	2-Aug-15	Ana Margarida Maria Monteiro	Isidoro dos Remedios Santana Mor
VALADARES	24-Nov-15	30-Nov-15	Etelvina Joaninha da Piedade Valadares	Vinancio Francisco da Piedade Vala
ARAUJO	10-Nov-15	5-May-16	Maria Quiteria Felicidade Araujo	Joao Caetano Sebastiao Eduardo A
MASCARENHAS	10-Aug-16	17-Aug-16	Maria Pasciencia Carolina Mascarenhas	Pedro Sebastiao Teofilo Mascarenh
MASCARENHAS	20-May-16	10-Sep-16	Constancio Bernardo Santana Mascarenhas	Jose Pedrinho Teotonio Mascarenh
MONTEIRO	6-Oct-16	18-Dec-16	Telruno Joao Baptista Lino Monteiro	Vinancio Bernardo Ubaldino Montei
MONTEIRO	27-Jan-17	1-Feb-17	Maria Teresa Herminia Gracias Monteiro	Cesar Baronio da Conceicao Monte
MONTEIRO	18-Mar-17	21-Mar-17	Alvaro Bossuet Monteiro	Joao Cosme Damiao Bossuet Mont
ARAUJO	15-May-17	22-May-17	Exaltacao Nicolau Mariano D'Araujo	Joao Caetano Sebastiao Eduardo A
ARAUJO	15-Sep-17	22-Sep-17	Amelia Prudencia Araujo	Jose Pascoal Araujo
MONTEIRO	11-Sep-17	28-Sep-17	Prudencia Helena Monteiro	Joaquim Heliodoro Bernadino Mont
MASCARENHAS	5-Mar-18	5-Mar-18	Vicente Caetano Francisco Mascarenhas	Jose Pedrinho Teotonio Mascarenh
MONTEIRO	12-Aug-18	18-Aug-18	Francisco Sebastiao Gracias Monteiro	Cesar Baronio da Conceicao Monte
MONTEIRO	20-Dec-18	26-Dec-18	Sedonio Pais Monteiro	Joaquim Heliodoro Bernadino Mont
MASCARENHAS	15-Oct-18	28-Dec-18	Leopoldinha Senhorinha Mascarenhas	Jose Salvador Mascarenhas
MASCARENHAS	31-Dec-18	8-Jan-19	Manuel Fr. Xavier Gavino Mascarenhas	Pedro Sebastiao Teofilo Mascarenh
MONTEIRO	19-Jan-19	27-Jan-19	Mario do Coracao de Jesus Bossuet Monteiro	Joao Cosme Damiao Bossuet Mont
ARAUJO	27-Dec-18	1-Mar-19	Joao Sebastiao de Jesus Araujo	Joaquim Mariano Hilario dos Milagr
MONTEIRO	8-Jan-19	1-Mar-19	Jose Joaquim Santana Monteiro	Joaquim Filipe de Araujo Monteiro
ARAUJO	3-Nov-18	19-May-19	Pedro Joaquim Sebastiao Araujo	Joao Caetano Sebastiao Eduardo A
ARAUJO	3-Aug-19	10-Aug-19	Teresinha Escolastica Araujo	Jose Pascoal Araujo
MONTEIRO	21-Oct-19	28-Oct-19	Hilario Joaquim Victorino Monteiro	Rosario Mariano Alleluia Monteiro
MONTEIRO	22-Nov-19	29-Nov-19	Rosario Manuel Monteiro	Vinancio Bernardo Ubaldino Montei
MASCARENHAS	26-Dec-19	27-Dec-19	Leandro Eusebio Mascarenhas	Pedro Sebastiao Teofilo Mascarenh
MONTEIRO	15-Dec-19	5-Jan-20	Maria Joaquina Irena Sebastiana Monteiro	Constancio Piedade Remigio Monte
MASCARENHAS	26-Mar-20	3-Apr-20	Constancio Salvador das Dores Mascarenhas	Marcos Francisco Aleixo Braz Masc
MONTEIRO	19-Jun-20	26-Jun-20	Antonio da Conceicao Gracias Monteiro	Cesar Baronio da Conceicao Monte
ARAUJO	12-Jul-20	26-Aug-20	Maria Alice Helena D'Araujo	Joao Caetano Sebastiao Eduardo A
MONTEIRO	12-Jan-22	18-Jan-22	Alfredo Inocencio Gracias Monteiro	Cesar Baronio da Conceicao Monte
VALADARES	26-Jan-22	26-Mar-22	Visitacao Merces Teresa Valadares	Roque Mariano Lourenco Valadares
MASCARENHAS	21-Mar-22	28-Mar-22	Placido Milagres Thome Mascarenhas	Pedro Sebastiao Teofilo Mascarenh
MONTEIRO	17-Jun-22	25-Jun-22	Analinda Fernandina Monteiro	Constancio Piedade Remigio Monte
MASCARENHAS	21-Oct-22	28-Oct-22	Narciso Sebastiao Mascarenhas	Miguel Joao Mascarenhas
ARAUJO	29-Nov-22	6-Dec-22	Constantino Roque Miguel Araujo	Milagres Santana Gaudino Araujo
MONTEIRO	17-Apr-23	24-Apr-23	Henriqueta Quiteria Divina Monteiro	Rosario Mariano Alleluia Monteiro
MONTEIRO	19-Jun-23	24-Jun-23	Maria Luciana Gracias Monteiro	Cesar Baronio da Conceicao Monte
VALADARES	30-Jun-23	7-Jul-23	Manuel Antonio Nicolau Valadares	Roque Mariano Lourenco Valadares

MONTEIRO	8-Jul-23	18-Jul-23	Jose Lino de Bossuet Monteiro	Joao Cosme Damiao Bossuet Mont
MASCARENHAS	26-Jul-23	24-Aug-23	Camilo Santana Ataide Mascarenhas	Constancio Mascarenhas
ARAUJO	8-Dec-23	15-Dec-23	Remedios Gondicalo Conceicao Araujo	Milagres Santana Gaudino Araujo
MONTEIRO	8-Mar-24	15-Mar-24	Inacio Bernadito Joao de Deus Monteiro	Joao Micael Nascimento Monteiro
MASCARENHAS	21-May-24	28-May-24	Maria Lina Rita Mascarenhas	Salvador do Rosario Erasmo Masca
MONTEIRO	31-Jan-25	9-Feb-25	Carlos Constancio Jesus Monteiro	Jose Sebastiao da Piedade Monteir
ARAUJO	31-Jan-21	4-Mar-25	Maria Emilia Filomena D'Araujo	Joao Caetano Sebastiao Eduardo A
ARAUJO	2-Feb-25	4-Mar-25	Luis Joao Gonzaga D'Araujo	Joao Caetano Sebastiao Eduardo A
MONTEIRO	12-Apr-25	19-Apr-25	Maria Margarida Gracias Monteiro	Cesar Baronio da Conceicao Monte
MASCARENHAS	27-Apr-25	4-May-25	Tertuliano Wenceslau Salvador Mascarenhas	Pedro Sebastiao Teofilo Mascarenh
MONTEIRO	24-Apr-25	5-May-25	Malito de Bossuet Monteiro	Joao Cosme Damiao Bossuet Mont
VALADARES	23-Jun-25	30-Jun-25	Ana Severina Valadares	Roque Mariano Lourenco Valadares
ARAUJO	30-Jun-25	7-Jul-25	Paulo Manuel Joaquim Araujo	Joaquim Mariano Hilario dos Milagr
ARAUJO	21-Dec-25	28-Dec-25	Miguel Salvador dos Milagres Araujo	Milagres Santana Gaudino Araujo
MASCARENHAS	23-Apr-26	30-Apr-26	Georgina Leticia Noronha Mascarenhas	Joao Crisostomo Santana Mascare
ARAUJO	14-Jun-26	10-Aug-26	Antonio Sebastiao Filipe Vilson de Araujo	Joaquim Mariano de Araujo
MONTEIRO	3-Sep-26	9-Sep-26	Maria Doroteia Gracias Monteiro	Cesar Baronio da Conceicao Monte
MONTEIRO	5-Nov-26	14-Nov-26	Jose Alberto Assiz Antonio Monteiro	Flaviano Policarpo Aleluia Antonio M
ARAUJO	6-Nov-26	18-Nov-26	Maria Emilia Araujo	Joao Caetano Sebastiao Eduardo A
MASCARENHAS	30-May-26	22-Nov-26	Maria Leticia Quadros e Mascarenhas	Constancio Roque Mascarenhas
MASCARENHAS	17-Nov-26	29-Nov-26	Angela Clementina Mascarenhas	Espirito Santo Antonio Francisco M
MASCARENHAS	5-Jul-27	17-Jul-27	Sebastiao Constancio Antonio Mascarenhas	Antonio Vicente Caetano Mascaren
ARAUJO	28-Jul-27	4-Aug-27	Narciso de Rosario Araujo	Caetano Piedade Floriano Araujo
MASCARENHAS	9-Jan-28	16-Jan-28	Aires Sebastiao Orlando Mascarenhas	Pedro Sebastiao Teofilo Mascarenh
MONTEIRO	18-Jan-28	25-Jan-28	Jovita Pedro Joao Jose Monteiro	Joao Micael Nascimento Monteiro
MASCARENHAS	1-Apr-28	9-Apr-28	Lourdes Honorata Noronha Mascarenhas	Joao Crisostomo Santana Mascare
MONTEIRO	20-May-28	27-May-28	Constancio Mariano Monteiro	Santana Manuel do Rosario Montei
ARAUJO	2-Apr-28	3-Jun-28	Constancio Roque Francisco Araujo	Milagres Santana Gaudino Araujo
MONTEIRO	28-Jun-28	28-Jun-28	Imelda Da Piedade Gracias Monteiro	Cesar Baronio da Conceicao Monte
MONTEIRO	29-Jun-28	6-Oct-28	Dulcina Monteiro	Roque Sebastiao Adriano Monteiro
MASCARENHAS	19-Apr-29	26-Apr-29	Maria Ofelia Olga Mascarenhas	Salvador do Rosario Erasmo Masca
MONTEIRO	15-Jul-29	22-Jul-29	Henriques Jose Antonio Monteiro	Flaviano Policarpo Aleluia Antonio M
MASCARENHAS	7-Aug-29	8-Sep-29	Teresinha Lina Mascarenhas	Pedro Sebastiao Teofilo Mascarenh
MONTEIRO	23-Sep-29	26-Sep-29	*Pe. Jose Lino Gracias Monteiro*	Cesar Baronio da Conceicao Monte
MONTEIRO	12-Apr-30	25-Apr-30	Maria Lila Filomena Teresinha Monteiro	Joao Cosme Damiao Bossuet Mont
MONTEIRO	28-Apr-30	5-May-30	Jose Feliciano Monteiro	Roque Sebastiao Adriano Monteiro
ARAUJO	12-May-30	17-May-30	Antonio Hipolito Araujo	Caetano Piedade Floriano Araujo
MONTEIRO	30-Aug-30	8-Sep-30	Maria Especiosa Rosa Monteiro	Sebastiao Manuel dos Remedios M
VALADARES	22-Aug-30	1-Dec-30	Elsa Maria Amelia Assuncao Valadares	Vicente Valadares
ARAUJO	17-Jan-31	5-Feb-31	Rui Antonio Craveiro Araujo	Joaquim Bernardo Lourenco Araujo
MONTEIRO	3-Oct-31	6-Oct-31	Teresa de Menino Jesus Gracias Monteiro	Cesar Baronio da Conceicao Monte
MONTEIRO	21-Nov-31	6-Dec-31	Maria Ermelinda Berta Monteiro	Sebastiao Manuel dos Remedios M
MONTEIRO	31-Dec-31	7-Jan-32	Maria Francisca Carolina Ofelia Monteiro	Joao Micael Nascimento Monteiro
ARAUJO	19-Jan-32	2-Feb-32	Maria Quiteria Joana Araujo	Milagres Santana Gaudino Araujo
ARAUJO	5-Nov-32	12-Nov-32	Maria Luisa Quiteria Etelvina Teresa Araujo	Joaquim Sebastiao Aleixo Piedade
MONTEIRO	23-Dec-32	5-Jan-33	Joaquim Victor Isidoro Monteiro	Filipe Salvador Nascimento Aleluia
ARAUJO	13-Jan-33	20-Jan-33	Clifton Sebastiao Felix Araujo	Joaquim Bernardo Lourenco Araujo
MASCARENHAS	8-Mar-33	15-Mar-33	Maria Ida Teresa Socorro Mascarenhas	Espirito Santo Antonio Francisco M
DE SA	16-May-33	20-Jun-33	Jose Ubaldo Antonio Matias de Sa	Jose Torcato de Sa
VALADARES	17-Jun-33	9-Sep-33	Francisco Xavier Valadares	Vicente Valadares
MONTEIRO	5-Sep-33	12-Sep-33	Maria Jose Augustilia dos Remedios Monteiro	Sebastiao Manuel dos Remedios M
MASCARENHAS	13-Mar-34	20-Mar-34	Aires Jose Caetano Mascarenhas	Pedro Sebastiao Teofilo Mascarenh
DE SA	6-Jun-34	13-Jun-34	Jose Luis Jeronimo de Sa	Jose Torcato de Sa
MASCARENHAS	29-Jan-35	5-Feb-35	Teresinha Socorro Mascarenhas	Espirito Santo Antonio Francisco M

DE SA	8-Jul-36	15-Jul-36	Jose Carmo Sebastiao da Piedade de Sa	Jose Torcato de Sa
VALADARES	15-Aug-36	30-Aug-36	Joaquim Mariano Assumcao Valadares	Abailardo Agostinho Espirito Santo
VALADARES	19-Apr-36	28-Jan-37	Alice de Santa Filomena Valadares	Vicente Valadares
MONTEIRO	18-Dec-44	10-Feb-45	Filomena Lourdes de Edna Monteiro	Nicolau Emilio Monteiro
ARAUJO	17-Feb-46	24-Feb-46	Maria Jacinta Fatima Alexandrina Araujo	Joao Sebastiao Expectacao de Jes
MONTEIRO	3-Sep-46	29-Sep-46	Joaquim Jose Heliodoro Monteiro	Sedonio Pais Monteiro
MONTEIRO	4-Dec-46	22-Dec-46	Amilcar Carlos Jose Manuel Monteiro	Julio Cesar Francisco Monteiro
ARAUJO	7-Mar-47	15-Apr-47	Maria Aura Angela Olivia Araujo	Bernardo Justino Araujo
ARAUJO	7-Jun-47	14-Jun-47	Joaquim Hilario Antonio Milagres Araujo	Joao Sebastiao Expectacao de Jes
MONTEIRO	22-Aug-47	3-Sep-47	Maria Eliza Yvette Monteiro	Sedonio Pais Monteiro
MONTEIRO	20-Sep-47	29-Sep-47	Margarida Priti Cordeiro Monteiro	Francisco Agostinho Gracias Monte
ARAUJO	13-Feb-48	22-Feb-48	Caetano Agnelo dos Milagres Araujo	Carlos Antonio Francisco dos Milag
MONTEIRO	30-Apr-48	16-May-48	Maria do Ceu Elvira Monteiro	Julio Cesar Francisco Monteiro
MONTEIRO	19-Aug-48	18-Sep-48	Pulqueria Odete Vijaia Monteiro	Nicolau Eduardo Monteiro
MONTEIRO	9-Nov-48	20-Nov-48	Fernando Dias Monteiro	Jose do Carmo Humberto Monteiro
ARAUJO	26-Apr-49	3-May-49	Maria Florinda Cleta Araujo	Joao Sebastiao Expectacao de Jes
MONTEIRO	23-Jun-49	3-Jul-49	Maria do Carmo Propercia Monteiro	Julio Cesar Francisco Monteiro
MONTEIRO	14-Jul-49	24-Jul-49	Boaventura das Merces Monteiro	Joao Baptista das Merces Monteiro
MONTEIRO	5-Sep-49	17-Sep-49	Oscar Joel Monteiro	Sedonio Pais Monteiro
ARAUJO	16-May-50	27-May-50	Manuel Eduardo Olavo D'Araujo	Exaltacao Nicolau Mariano D'Arauj

Table S6. **Electoral rolls for (convert) KAMAT and PAI lineages in Kudtari and Lotli towns (2013)**

KUDTARI:

Vangor	Original	Current	Number
1	KAMAT	MENEZES	173
1	KAMAT	BORGES	44
3	KAMAT	VAS	198
5	KAMAT	DINIZ	34
8	KAMAT	PIMENTA	17
9	KAMAT	ESTIBEIRO	54
12	KAMAT	BARRETO	155
14	KAMAT	VEIGA	18
15	KAMAT	PAES	79
16	KAMAT	VIEGAS	32
		Total electorate:	9475

LOTLI:

Vangor	Original	Current	Number
2	PAI	DA COSTA	2
5	PAI	VALADARES	0
7	PAI	DE SA	0
8	PAI	MASCARENHAS	50
10	PAI	COLACO	0
12	PAI	ARAUJO	46
15	PAI	MONTEIRO	54
		Total electorate:	4587

Source: http://ceogoa.nic.in
(downloaded 10/1/2013)

Table S7. **TMRCA branch-point histogram table.** Pairwise TMRCA computations were performed as described in Methods. Each pairwise value was computed using the modal reference of vangor 8 (Table 1) against each R1a1 member of the communities listed. The TMRCAs were grouped at 600 year intervals between 1500 BCE and 1500 CE. Results are expressed as the percentage of the total sample for each time period.

	GSB-K	Khatri	Zabuli	Pashtun	Gandhara	Gulf	S. Asia	Bactria	Tajik	Saraswat
**n**	**9**	**7**	**23**	**15**	**29**	**42**	**19**	**12**	**11**	**13**
**[illegible]1500 BCE**	0.0	0.0	13.0	**20.0**	**20.7**	14.3	**26.3**	0.0	**36.4**	**53.8**
**[illegible]00-1500 BCE**	0.0	0.0	13.0	**33.3**	13.8	**31.0**	**26.3**	**50.0**	**54.5**	**23.1**
**[illegible]00-900 BCE**	0.0	**28.6**	**47.8**	**26.7**	**62.1**	**52.4**	**42.1**	**50.0**	9.1	7.7
**[illegible]00BCE-300CE**	0.0	**28.6**	**26.1**	**20.0**	3.4	2.4	5.3	0.0	0.0	7.7
**[illegible]00-900 CE**	**33.3**	**42.9**	0.0	0.0	0.0	0.0	0.0	0.0	0.0	7.7
**[illegible]00-1500 CE**	**33.3**	0.0	0.0	0.0	0.0	0.0	0.0	0.0	0.0	0.0
**[illegible]500-present**	**33.3**	0.0	0.0	0.0	0.0	0.0	0.0	0.0	0.0	0.0

Key: Pre-Vedic (<1500 BCE); Vedic (1500-900 BCE); Classical (900-300 BCE); Early Historical (300 BCE-300 CE); Brahmin Migration (300-900 CE); Late Medieval (900-1500 CE); Western Colonial (>1500 BCE).

Table S8. **657A incidence table.** R1a1 individuals from each cohort were examined for 657A type (R1a1 Y-HG individuals carrying the STR profile: DYS456<16, DYS458>15, GATAH4>12).

	**Group or Region**	**ID**	**Regions / Communities**	**Reference**	**R1a1 (n)**	**L657**	**Pct 657A**
1	LPK	GSB	GSB, Goa (India)	This work	9	Yes	**66.7**
2	Khatri	KHT	Punjab (India)	This work	7	Yes	**57.1**
3	Zabulistan/Arachosia	ZAB	Helmand, Ghazni, Afghanistan	ref 1	29	n.a.	**31.0**
4	Pashtun	PST	Afghanistan	ref 2	25	n.a.	**8.0**
5	Gandhara	GDH	Khyber Pass Region, Afghanistan	ref 1	30	n.a.	**10.0**
6	Balkh/Bactria	BCT	Kunduz, Mazar e Sharif, Afghanistan	ref 1	12	n.a.	**41.7**
7	Gulf States	GLF	Saudi Arabia, Kuwait, Bahrain, Qatar, UAE	Public db	40	Yes	**2.5**
8	Sub-continent	SCT	Pakistan, India (except Konkan)	Public db	22	Yes	**0.0**
9	Saraswat Brahmins	PSB	India (Punjab, HP)	Ref 3	45	Yes	n/a
10	Tajik	TJK	Afghanistan	ref 2	17	n.a.	**17.6**
11	West Transcaucasia	WTC	Abkhaz, Circassian, Shapsug	ref 4	27	n.a.	**0.0**
12	Nakh group	NKH	Chechen, Ingush	ref 4	18	n.a.	**0.0**
13	East Transcaucasia	ETC	Avar, Dargins, Lezghins	ref 4	27	n.a.	**55.6**

References:

1. Lacau H, Bukhari A, Gayden T, La Salvia J, Regueiro M, Stojkovic O, Herrera RJ. (2011) Y-STR profiling in two Afghanistan populations. *Legal Med (Tokyo).* 13(2):103-108.
2. Lacau H, Gayden T, Regueiro M, Chennakrishnaiah S, Bukhari A, Underhill PA, Garcia-Bertrand RL, Herrera RJ. (2012) Afghanistan from a Y-chromosome perspective. *Eur J Hum Genet.* 20(10): 1063-1070.
3. Yadav B, Raina A, Dogra TD. (2011) Haplotype diversity of 17 Y-chromosomal STRs in Saraswat Brahmin Community of North India. *Forensic Sci Int Genet*. 5(3): e63-70.
4. Balanovsky O, Dibirova K, Dybo A, Mudrak O, Frolova S, Pocheshkhova E, Haber M, Platt D, Schurr T, Haak W, Kuznetsova M, Radzhabov M, Balaganskaya O, Romanov A, Zakharova T, Soria Hernanz DF, Zalloua P, Koshel S, Ruhlen M, Renfrew C, Wells RS, Tyler-Smith C, Balanovska E; Genographic Consortium. (2011) Parallel evolution of genes and languages in the Caucasus region. *Mol Biol Evol*. 28(10): 2905-2920.

Table S9. **R1a1 subclades by geographic region.** Percent geographic incidence of each major R1a1 clade in the public database (October 2012); TC=Transcaucasia. To avoid contribution bias, the relative percentage for each class is shown.

	R1a1 subclade (n)				
**GION**	**M458 (133)**	**Z280 (185)**	**Z93 L657- (57)**	**Z93+L657+ (27)**	**All R1a1 (2417)**
DITERRANEAN	0.8	2.7	5.3	0.0	1.7
ATOLIA-TC	0.8	2.2	24.6	0.0	1.2
RSIAN GULF	0.0	0.5	19.3	59.3	3.6
ASIA	0.0	0.0	5.3	37.0	1.2
LKAN-BALTIC	78.2	50.8	10.5	0.0	42.0
STEPPES	19.5	38.9	17.5	3.7	22.1
EUROPE	0.8	4.9	17.5	0.0	28.3

Table S10. **Centroid data table**

DB ID#	Country	HG	Clade	LAT	LONG
194691	Afghanistan	J2a4*	J-L26 M67-	34.5	69.2
217812	Armenia	J2a4*	J-L26 M67-	40.2	44.5
166329	Armenia	J2a4*	J-L26 M67-	40.2	44.5
181393	Armenia	J2a4*	J-L26 M67-	40.2	44.5
E4955	Austria	J2a4*	J-L26 M67-	48.2	16.4
N96517	Austria	J2a4*	J-L26 M67-	48.2	16.4
N74818	Belgium	J2a4*	J-L26 M67-	50.8	4.4
N5341	Bulgaria	J2a4*	J-L26 M67-	42.7	23.3
105169	Finland	J2a4*	J-L26 M67-	60.2	24.9
41120	France	J2a4*	J-L26 M67-	48.9	2.4
122074	France	J2a4*	J-L26 M67-	48.9	2.4
N6276	France	J2a4*	J-L26 M67-	48.9	2.4
44036	Germany	J2a4*	J-L26 M67-	52.5	13.4
103233	Germany	J2a4*	J-L26 M67-	52.5	13.4
71974	India	J2a4*	J-L26 M67-	21	78
N3437	India	J2a4*	J-L26 M67-	21	78
N84214	India	J2a4*	J-L26 M67-	21	78
E12430	Iran	J2a4*	J-L26 M67-	35.7	51.4
N68809	Iran	J2a4*	J-L26 M67-	35.7	51.4
237306	Ireland	J2a4*	J-L26 M67-	53.3	6.3
182443	Ireland	J2a4*	J-L26 M67-	53.3	6.3
169514	Italy	J2a4*	J-L26 M67-	41.9	12.5
205145	Italy	J2a4*	J-L26 M67-	41.9	12.5
182122	Italy	J2a4*	J-L26 M67-	41.9	12.5
N100355	Kuwait	J2a4*	J-L26 M67-	29.4	47.9
168722	Levant	J2a4*	J-L26 M67-	33.4	35.5
204156	Levant	J2a4*	J-L26 M67-	33.4	35.5
N74325	Levant	J2a4*	J-L26 M67-	33.4	35.5
180589	Levant	J2a4*	J-L26 M67-	33.4	35.5
186929	Levant	J2a4*	J-L26 M67-	33.4	35.5
212279	Levant	J2a4*	J-L26 M67-	33.4	35.5
E9560	Levant	J2a4*	J-L26 M67-	33.4	35.5
109098	Poland	J2a4*	J-L26 M67-	52.2	21
115615	Qatar	J2a4*	J-L26 M67-	25.3	51.5
207796	Saudi Arabia	J2a4*	J-L26 M67-	24.6	46.7
149554	Saudi Arabia	J2a4*	J-L26 M67-	24.6	46.7
M7436	Saudi Arabia	J2a4*	J-L26 M67-	24.6	46.7
182069	Spain	J2a4*	J-L26 M67-	40.4	-3.7
139704	Turkey	J2a4*	J-L26 M67-	39.9	32.9
160878	Turkey	J2a4*	J-L26 M67-	39.9	32.9
184386	Turkey	J2a4*	J-L26 M67-	39.9	32.9
216103	France	J2a4*	J-L26 M67-	48.9	2.4
202853	Portugal	J2a4*	J-L26 M67-	38.8	-9.1
229980	Qatar	J2a4*	J-L26 M67-	25.3	51.5
M7239	Qatar	J2a4*	J-L26 M67-	25.3	51.5
216669	Qatar	J2a4*	J-L26 M67-	25.3	51.5
M7808	Saudi Arabia	J2a4*	J-L26 M67-	24.6	46.7
E7054	Slovenia	J2a4*	J-L26 M67-	46.1	14.5
112392	UK	J2a4*	J-L26 M67-	51.5	0.1
205250	UAE	J2a4*	J-L26 M67-	24.5	54.4

N37321	UK	J2a4*	J-L26 M67-	51.5	0.1
M6992	Algeria	J2a4a	J-M47	36.7	3.2
101499	Armenia	J2a4a	J-M47	40.2	44.5
M6329	Iran	J2a4a	J-M47	35.7	51.4
M6737	Iran	J2a4a	J-M47	35.7	51.4
96761	Italy	J2a4a	J-M47	41.9	12.5
161273	Kuwait	J2a4a	J-M47	29.4	47.9
191840	Kuwait	J2a4a	J-M47	29.4	47.9
229690	Kuwait	J2a4a	J-M47	29.4	47.9
M6426	Kuwait	J2a4a	J-M47	29.4	47.9
M7429	Kuwait	J2a4a	J-M47	29.4	47.9
193791	Levant	J2a4a	J-M47	33.4	35.5
79848	Luxembourg	J2a4a	J-M47	49.6	6.1
236171	Qatar	J2a4a	J-M47	25.3	51.5
M6715	Qatar	J2a4a	J-M47	25.3	51.5
149703	Saudi Arabia	J2a4a	J-M47	24.6	46.7
165654	Saudi Arabia	J2a4a	J-M47	24.6	46.7
169860	Saudi Arabia	J2a4a	J-M47	24.6	46.7
169882	Saudi Arabia	J2a4a	J-M47	24.6	46.7
198885	Saudi Arabia	J2a4a	J-M47	24.6	46.7
232941	Saudi Arabia	J2a4a	J-M47	24.6	46.7
233703	Saudi Arabia	J2a4a	J-M47	24.6	46.7
234783	Saudi Arabia	J2a4a	J-M47	24.6	46.7
M6642	Saudi Arabia	J2a4a	J-M47	24.6	46.7
M7065	Saudi Arabia	J2a4a	J-M47	24.6	46.7
M7380	Saudi Arabia?	J2a4a	J-M47	24.6	46.7
M3626	UAE	J2a4a	J-M47	24.5	54.4
M3701	UAE	J2a4a	J-M47	24.5	54.4
M4272	UAE	J2a4a	J-M47	24.5	54.4
M6903	UAE	J2a4a	J-M47	24.5	54.4
N57035	UK	J2a4a	J-M47	51.5	0.1
174248	Armenia	J2a4b	J-M67	40.2	44.5
174250	Armenia	J2a4b	J-M67	40.2	44.5
N64462	Armenia	J2a4b	J-M67	40.2	44.5
230305	Chechnya	J2a4b	J-M67	43.3	45.6
216529	Georgia	J2a4b	J-M67	41.7	44.8
31852	Germany	J2a4b	J-M67	52.5	13.4
36603	Germany	J2a4b	J-M67	52.5	13.4
29156	Hungary	J2a4b	J-M67	47.4	19.2
N11949	Iran	J2a4b	J-M67	35.7	51.4
N30461	Iran	J2a4b	J-M67	35.7	51.4
70513	Iraq	J2a4b	J-M67	33.3	44.4
31879	Ireland	J2a4b	J-M67	53.3	6.3
57210	Ireland	J2a4b	J-M67	53.3	6.3
144646	Ireland	J2a4b	J-M67	53.3	6.3
N53046	Ireland	J2a4b	J-M67	53.3	6.3
18790	Italy	J2a4b	J-M67	41.9	12.5
72837	Italy	J2a4b	J-M67	41.9	12.5
86636	Italy	J2a4b	J-M67	41.9	12.5
96663	Italy	J2a4b	J-M67	41.9	12.5
E1878	Italy	J2a4b	J-M67	41.9	12.5
N16888	Italy	J2a4b	J-M67	41.9	12.5
N60728	Italy	J2a4b	J-M67	41.9	12.5
N68160	Italy	J2a4b	J-M67	41.9	12.5

N70674	Italy	J2a4b	J-M67	41.9	12.5
N89155	Italy	J2a4b	J-M67	41.9	12.5
149533	Levant	J2a4b	J-M67	33.4	35.5
152050	Levant	J2a4b	J-M67	33.4	35.5
176688	Levant	J2a4b	J-M67	33.4	35.5
N22510	Levant	J2a4b	J-M67	33.4	35.5
103307	Netherlands	J2a4b	J-M67	52.4	4.9
21636	Poland	J2a4b	J-M67	52.2	21
70515	Poland	J2a4b	J-M67	52.2	21
129384	Portugal	J2a4b	J-M67	38.8	-9.1
75164	Romania	J2a4b	J-M67	44.4	26.1
230560	Russia	J2a4b	J-M67	55.7	37.6
198821	Syria	J2a4b	J-M67	33.5	36.3
164196	Turkey	J2a4b	J-M67	39.9	32.9
169448	Turkey	J2a4b	J-M67	39.9	32.9
N15937	Turkey	J2a4b	J-M67	39.9	32.9
N59703	Turkey	J2a4b	J-M67	39.9	32.9
96049	UK	J2a4b	J-M67	51.5	0.1
123323	UK	J2a4b	J-M67	51.5	0.1
71893	Ukraine	J2a4b	J-M67	50.4	30.5
149200	Armenia	J2a4b1	J-M92	40.2	44.5
207705	Armenia	J2a4b1	J-M92	40.2	44.5
N12720	Azerbaijan	J2a4b1	J-M92	40.4	49.8
69509	Belarus	J2a4b1	J-M92	53.9	27.6
147048	Germany	J2a4b1	J-M92	52.5	13.4
N11704	Germany	J2a4b1	J-M92	52.5	13.4
200837	Germany	J2a4b1	J-M92	52.5	13.4
120539	Germany	J2a4b1	J-M92	52.5	13.4
28730	Germany	J2a4b1	J-M92	52.5	13.4
70140	Germany	J2a4b1	J-M92	52.5	13.4
147721	Germany	J2a4b1	J-M92	52.5	13.4
45403	Hungary	J2a4b1	J-M92	47.4	19.2
50911	Hungary	J2a4b1	J-M92	47.4	19.2
N93774	Hungary	J2a4b1	J-M92	47.4	19.2
228035	Hungary	J2a4b1	J-M92	47.4	19.2
118655	Hungary	J2a4b1	J-M92	47.4	19.2
48340	Iran	J2a4b1	J-M92	35.7	51.4
107714	Italy	J2a4b1	J-M92	41.9	12.5
118717	Italy	J2a4b1	J-M92	41.9	12.5
123370	Italy	J2a4b1	J-M92	41.9	12.5
E14637	Italy	J2a4b1	J-M92	41.9	12.5
65112	Italy	J2a4b1	J-M92	41.9	12.5
162181	Italy	J2a4b1	J-M92	41.9	12.5
343	Lithuania	J2a4b1	J-M92	54.7	25.3
164237	Lithuania	J2a4b1	J-M92	54.7	25.3
132673	Luxembourg	J2a4b1	J-M92	49.6	6.1
39654	Netherlands	J2a4b1	J-M92	52.4	4.9
43553	Poland	J2a4b1	J-M92	52.2	21
100995	Poland	J2a4b1	J-M92	52.2	21
108915	Poland	J2a4b1	J-M92	52.2	21
209229	Poland	J2a4b1	J-M92	52.2	21
52473	Portugal	J2a4b1	J-M92	38.8	-9.1
88790	Portugal	J2a4b1	J-M92	38.8	-9.1
76212	Romania	J2a4b1	J-M92	44.4	26.1

145757	Romania	J2a4b1	J-M92	44.4	26.1
174944	Romania	J2a4b1	J-M92	44.4	26.1
171079	Romania	J2a4b1	J-M92	44.4	26.1
112842	Romania	J2a4b1	J-M92	44.4	26.1
174705	Russia	J2a4b1	J-M92	55.7	37.6
N15777	Russia	J2a4b1	J-M92	55.7	37.6
157250	Russia	J2a4b1	J-M92	55.7	37.6
112303	Russia	J2a4b1	J-M92	55.7	37.6
M7226	Saudi Arabia	J2a4b1	J-M92	24.6	46.7
M5017	Saudi Arabia	J2a4b1	J-M92	24.6	46.7
N93603	Serbia	J2a4b1	J-M92	44.8	20.4
112883	Spain	J2a4b1	J-M92	40.4	-3.7
N3597	Spain	J2a4b1	J-M92	40.4	-3.7
91003	Spain	J2a4b1	J-M92	40.4	-3.7
164217	Turkey	J2a4b1	J-M92	39.9	32.9
166324	Turkey	J2a4b1	J-M92	39.9	32.9
N57415	Turkey	J2a4b1	J-M92	39.9	32.9
N75792	Turkey	J2a4b1	J-M92	39.9	32.9
N90067	Turkey	J2a4b1	J-M92	39.9	32.9
N19693	UK	J2a4b1	J-M92	51.5	0.1
106553	UK	J2a4b1	J-M92	51.5	0.1
163	Ukraine	J2a4b1	J-M92	50.4	30.5
5984	Ukraine	J2a4b1	J-M92	50.4	30.5
119606	Ukraine	J2a4b1	J-M92	50.4	30.5
119546	Ukraine	J2a4b1	J-M92	50.4	30.5
N69190	Ukraine	J2a4b1	J-M92	50.4	30.5
20447	Belgium	L1a	L-M27+	50.8	4.4
46274	Germany	L1a	L-M27+	52.5	13.4
131191	Germany	L1a	L-M27+	52.5	13.4
N76271	India	L1a	L-M27+	21	78
69078	India	L1a	L-M27+	21	78
89330	India	L1a	L-M27+	21	78
102209	India	L1a	L-M27+	21	78
133264	India	L1a	L-M27+	21	78
N13642	India	L1a	L-M27+	21	78
N15319	India	L1a	L-M27+	21	78
N3831	India	L1a	L-M27+	21	78
N39694	India	L1a	L-M27+	21	78
N43666	India	L1a	L-M27+	21	78
N74752	India	L1a	L-M27+	21	78
N82752	India	L1a	L-M27+	21	78
N98608	India	L1a	L-M27+	21	78
N9890	India	L1a	L-M27+	21	78
76304	India	L1a	L-M27+	21	78
N10580	India	L1a	L-M27+	21	78
N88841	India	L1a	L-M27+	21	78
N92407	Iran	L1a	L-M27+	35.7	51.4
190284	Iraq	L1a	L-M27+	33.3	44.4
E6065	Italy	L1a	L-M27+	41.9	12.5
204204	Kuwait	L1a	L-M27+	29.4	47.9
M7213	Kuwait	L1a	L-M27+	29.4	47.9
N26601	Levant	L1a	L-M27+	33.4	35.5
M6412	Levant	L1a	L-M27+	33.4	35.5
186143	Malaysia	L1a	L-M27+	3.1	101.7

N12495	Pakistan	L1a	L-M27+	33.7	73.2
N5937	Pakistan	L1a	L-M27+	33.7	73.2
N63576	Pakistan	L1a	L-M27+	33.7	73.2
54993	Saudi Arabia	L1a	L-M27+	24.6	46.7
224572	Saudi Arabia	L1a	L-M27+	24.6	46.7
180230	Spain	L1a	L-M27+	40.4	-3.7
222595	Syria	L1a	L-M27+	33.5	36.3
M6704	UAE	L1a	L-M27+	24.5	54.4
M6396	UAE	L1a	L-M27+	24.5	54.4
222106	UAE	L1a	L-M27+	24.5	54.4
M6852	UAE	L1a	L-M27+	24.5	54.4
189917	Armenia	L1b*	M295+ M317+	40.2	44.5
155684	Armenia	L1b*	M295+ M317+	40.2	44.5
181374	Bulgaria	L1b*	M295+ M317+	42.7	23.3
172987	Georgia	L1b*	M295+ M317+	41.7	44.8
N94640	Greece	L1b*	M295+ M317+	37.9	23.7
95658	Greece	L1b*	M295+ M317+	37.9	23.7
N30297	Greece	L1b*	M295+ M317+	37.9	23.7
36064	Russia	L1b*	M295+ M317+	55.7	37.6
N29672	Russia	L1b*	M295+ M317+	55.7	37.6
155681	Turkey	L1b*	M295+ M317+	39.9	32.9
N93829	Turkey	L1b*	M295+ M317+	39.9	32.9
208461	Turkey	L1b*	M295+ M317+	39.9	32.9
N25240	Turkey	L1b*	M295+ M317+	39.9	32.9
183217	Turkey	L1b*	M295+ M317+	39.9	32.9
N105310	Turkey	L1b*	M295+ M317+	39.9	32.9
97848	Turkey	L1b*	M295+ M317+	39.9	32.9
166328	Armenia	L1b1*	L-L656+ L349-	40.2	44.5
E10323	Austria	L1b1*	L-L656+ L349-	48.2	16.4
2700	Cyprus	L1b1*	L-L656+ L349-	35.1	33.4
N36293	Hungary	L1b1*	L-L656+ L349-	47.4	19.2
188710	Iraq	L1b1*	L-L656+ L349-	33.3	44.4
131738	Ireland	L1b1*	L-L656+ L349-	53.3	6.3
214163	Italy	L1b1*	L-L656+ L349-	41.9	12.5
199893	Italy	L1b1*	L-L656+ L349-	41.9	12.5
M7148	Kuwait	L1b1*	L-L656+ L349-	29.4	47.9
192340	Lebanon	L1b1*	L-L656+ L349-	33.4	35.5
43426	Lebanon	L1b1*	L-L656+ L349-	33.4	35.5
E9533	Lebanon	L1b1*	L-L656+ L349-	33.4	35.5
192812	Portugal	L1b1*	L-L656+ L349-	38.8	-9.1
177996	UK	L1b1*	L-L656+ L349-	51.5	0.1
189701	Armenia	L1b1a	L-M349	40.2	44.5
N65296	Austria	L1b1a	L-M349	48.2	16.4
N23180	Belarus	L1b1a	L-M349	53.9	27.6
N35596	Belgium	L1b1a	L-M349	50.8	4.4
N61542	Belgium	L1b1a	L-M349	50.8	4.4
1929	Egypt	L1b1a	L-M349	30.1	31.2
E14514	France	L1b1a	L-M349	48.9	2.4
E9935	Germany	L1b1a	L-M349	52.5	13.4
42998	Germany	L1b1a	L-M349	52.5	13.4
E6106	Germany	L1b1a	L-M349	52.5	13.4
199082	Germany	L1b1a	L-M349	52.5	13.4
204455	Germany	L1b1a	L-M349	52.5	13.4
87087	Hungary	L1b1a	L-M349	47.4	19.2

N13722	Italy	L1b1a	L-M349	41.9	12.5
174541	Italy	L1b1a	L-M349	41.9	12.5
206245	Italy	L1b1a	L-M349	41.9	12.5
N31383	Italy	L1b1a	L-M349	41.9	12.5
47636	Italy	L1b1a	L-M349	41.9	12.5
160419	Italy	L1b1a	L-M349	41.9	12.5
N40167	Levant	L1b1a	L-M349	33.4	35.5
N87113	Levant	L1b1a	L-M349	33.4	35.5
220233	Levant	L1b1a	L-M349	33.4	35.5
50919	Levant	L1b1a	L-M349	33.4	35.5
N16892	Poland	L1b1a	L-M349	52.2	21
N36807	Portugal	L1b1a	L-M349	38.8	-9.1
N14889	Portugal	L1b1a	L-M349	38.8	-9.1
150532	Romania	L1b1a	L-M349	44.4	26.1
133667	Spain	L1b1a	L-M349	40.4	-3.7
45035	Switzerland	L1b1a	L-M349	46.9	7.4
N36437	Switzerland	L1b1a	L-M349	46.9	7.4
N22678	Switzerland	L1b1a	L-M349	46.9	7.4
133397	Switzerland	L1b1a	L-M349	46.9	7.4
N34974	Switzerland	L1b1a	L-M349	46.9	7.4
117926	Switzerland	L1b1a	L-M349	46.9	7.4
84040	Switzerland	L1b1a	L-M349	46.9	7.4
158227	Switzerland	L1b1a	L-M349	46.9	7.4
119235	Turkey	L1b1a	L-M349	39.9	32.9
94500	Turkey	L1b1a	L-M349	39.9	32.9
207728	Turkey	L1b1a	L-M349	39.9	32.9
10566	UK	L1b1a	L-M349	51.5	0.1
126095	UK	L1b1a	L-M349	51.5	0.1
N13579	Afghanistan	L1c	L-M357	34.5	69.2
47957	Afghanistan	L1c	L-M357	34.5	69.2
117209	Afghanistan	L1c	L-M357	34.5	69.2
150710	Afghanistan	L1c	L-M357	34.5	69.2
N15249	Afghanistan	L1c	L-M357	34.5	69.2
N67050	Afghanistan	L1c	L-M357	34.5	69.2
228196	Armenia	L1c	L-M357	40.2	44.5
39112	India	L1c	L-M357	21	78
130229	India	L1c	L-M357	21	78
134699	India	L1c	L-M357	21	78
159251	India	L1c	L-M357	21	78
171543	India	L1c	L-M357	21	78
83541	India	L1c	L-M357	21	78
165503	India	L1c	L-M357	21	78
N16084	India	L1c	L-M357	21	78
N90087	India	L1c	L-M357	21	78
112920	India	L1c	L-M357	21	78
N91548	India	L1c	L-M357	21	78
193345	Iraq	L1c	L-M357	33.3	44.4
94149	Italy	L1c	L-M357	41.9	12.5
M7326	Kuwait	L1c	L-M357	29.4	47.9
M7428	Kuwait	L1c	L-M357	29.4	47.9
M7548	Kuwait	L1c	L-M357	29.4	47.9
1318	Pakistan	L1c	L-M357	33.7	73.2
57970	Pakistan	L1c	L-M357	33.7	73.2
69056	Pakistan	L1c	L-M357	33.7	73.2

69067	Pakistan	L1c	L-M357	33.7	73.2
110998	Pakistan	L1c	L-M357	33.7	73.2
N54263	Pakistan	L1c	L-M357	33.7	73.2
N74967	Pakistan	L1c	L-M357	33.7	73.2
M6759	Saudi Arabia	L1c	L-M357	24.6	46.7
M7661	Saudi Arabia	L1c	L-M357	24.6	46.7
176674	Armenia	Q1a2	Q-M25	40.2	44.5
41920	Germany	Q1a2	Q-M25	52.5	13.4
79694	Hungary	Q1a2	Q-M25	47.4	19.2
155314	Hungary	Q1a2	Q-M25	47.4	19.2
155304	Hungary	Q1a2	Q-M25	47.4	19.2
155308	Hungary	Q1a2	Q-M25	47.4	19.2
109759	Hungary	Q1a2	Q-M25	47.4	19.2
79135	Hungary	Q1a2	Q-M25	47.4	19.2
79133	Hungary	Q1a2	Q-M25	47.4	19.2
216493	India	Q1a2	Q-M25	21	78
N47526	Iran	Q1a2	Q-M25	35.7	51.4
53601	Ireland	Q1a2	Q-M25	53.3	6.3
N20234	Italy	Q1a2	Q-M25	41.9	12.5
178386	Mongolia	Q1a2	Q-M25	47.9	106.9
N94588	Poland	Q1a2	Q-M25	52.2	21
180029	Poland	Q1a2	Q-M25	52.2	21
167321	Russia	Q1a2	Q-M25	55.7	37.6
39998	Russia	Q1a2	Q-M25	55.7	37.6
199034	Russia	Q1a2	Q-M25	55.7	37.6
N71504	Russia	Q1a2	Q-M25	55.7	37.6
176187	Saudi Arabia	Q1a2	Q-M25	24.6	46.7
88698	Afghanistan	Q1a3*	M346+ M3- L213-	34.5	69.2
218769	Armenia	Q1a3*	M346+ M3- L213-	40.2	44.5
N84508	Bosnia	Q1a3*	M346+ M3- L213-	43.9	18.4
19564	Germany	Q1a3*	M346+ M3- L213-	52.5	13.4
N88758	Germany	Q1a3*	M346+ M3- L213-	52.5	13.4
160059	Hungary	Q1a3*	M346+ M3- L213-	47.4	19.2
47960	India	Q1a3*	M346+ M3- L213-	21	78
152470	India	Q1a3*	M346+ M3- L213-	21	78
N20284	India	Q1a3*	M346+ M3- L213-	21	78
N45070	India	Q1a3*	M346+ M3- L213-	21	78
N57946	India	Q1a3*	M346+ M3- L213-	21	78
N61141	India	Q1a3*	M346+ M3- L213-	21	78
N64267	India	Q1a3*	M346+ M3- L213-	21	78
N68722	India	Q1a3*	M346+ M3- L213-	21	78
N73100	India	Q1a3*	M346+ M3- L213-	21	78
139352	Ireland	Q1a3*	M346+ M3- L213-	53.3	6.3
234338	Italy	Q1a3*	M346+ M3- L213-	41.9	12.5
170259	Lithuania	Q1a3*	M346+ M3- L213-	54.7	25.3
185943	Norway	Q1a3*	M346+ M3- L213-	59.9	10.7
N31676	Norway	Q1a3*	M346+ M3- L213-	59.9	10.7
N65568	Pakistan	Q1a3*	M346+ M3- L213-	33.7	73.2
128283	Poland	Q1a3*	M346+ M3- L213-	52.2	21
166847	Portugal	Q1a3*	M346+ M3- L213-	38.8	-9.1
213436	Russia	Q1a3*	M346+ M3- L213-	55.7	37.6
160290	Saudi Arabia	Q1a3*	M346+ M3- L213-	24.6	46.7
29279	Sweden	Q1a3*	M346+ M3- L213-	59.4	18.1
E79WK	Sweden	Q1a3*	M346+ M3- L213-	59.4	18.1

N92580	Sweden	Q1a3*	M346+ M3- L213-	59.4	18.1
190477	Turkey	Q1a3*	M346+ M3- L213-	39.9	32.9
194161	Turkey	Q1a3*	M346+ M3- L213-	39.9	32.9
N62232	Turkey	Q1a3*	M346+ M3- L213-	39.9	32.9
144820	UAE	Q1a3*	M346+ M3- L213-	24.5	54.4
188317	UAE	Q1a3*	M346+ M3- L213-	24.5	54.4
M4285	UAE	Q1a3*	M346+ M3- L213-	24.5	54.4
48619	UK	Q1a3*	M346+ M3- L213-	51.5	0.1
113515	UK	Q1a3*	M346+ M3- L213-	51.5	0.1
147201	UK	Q1a3*	M346+ M3- L213-	51.5	0.1
N24383	UK	Q1a3*	M346+ M3- L213-	51.5	0.1
N32407	UK	Q1a3*	M346+ M3- L213-	51.5	0.1
N6045	UK	Q1a3*	M346+ M3- L213-	51.5	0.1
162168	Germany	Q1a3a*	Q-M346+ L213+ L804+ M3-	52.5	13.4
28136	Ireland	Q1a3a*	Q-M346+ L213+ L804+ M3-	53.3	6.3
30599	Ireland	Q1a3a*	Q-M346+ L213+ L804+ M3-	53.3	6.3
185943	Norway	Q1a3a*	Q-M346+ L213+ L804+ M3-	59.9	10.7
N31676	Norway	Q1a3a*	Q-M346+ L213+ L804+ M3-	59.9	10.7
58079	Norway	Q1a3a*	Q-M346+ L213+ L804+ M3-	59.9	10.7
65198	Norway	Q1a3a*	Q-M346+ L213+ L804+ M3-	59.9	10.7
200099	Norway	Q1a3a*	Q-M346+ L213+ L804+ M3-	59.9	10.7
N50723	Sweden	Q1a3a*	Q-M346+ L213+ L804+ M3-	59.4	18.1
N3263	Sweden	Q1a3a*	Q-M346+ L213+ L804+ M3-	59.4	18.1
101297	Sweden	Q1a3a*	Q-M346+ L213+ L804+ M3-	59.4	18.1
N23068	Sweden	Q1a3a*	Q-M346+ L213+ L804+ M3-	59.4	18.1
N38159	Sweden	Q1a3a*	Q-M346+ L213+ L804+ M3-	59.4	18.1
N92580	Sweden	Q1a3a*	Q-M346+ L213+ L804+ M3-	59.4	18.1
29279	Sweden	Q1a3a*	Q-M346+ L213+ L804+ M3-	59.4	18.1
48315	UK	Q1a3a*	Q-M346+ L213+ L804+ M3-	51.5	0.1
147201	UK	Q1a3a*	Q-M346+ L213+ L804+ M3-	51.5	0.1
N5430	UK	Q1a3a*	Q-M346+ L213+ L804+ M3-	51.5	0.1
N101906	UK	Q1a3a*	Q-M346+ L213+ L804+ M3-	51.5	0.1
113515	UK	Q1a3a*	Q-M346+ L213+ L804+ M3-	51.5	0.1
228656	UK	Q1a3a*	Q-M346+ L213+ L804+ M3-	51.5	0.1
N40362	UK	Q1a3a*	Q-M346+ L213+ L804+ M3-	51.5	0.1
34900	Belarus	Q1b1a	Q-L245	53.9	27.6
20102	Belarus	Q1b1a	Q-L245	53.9	27.6
N15254	Bosnia	Q1b1a	Q-L245	43.9	18.4
3791	Estonia	Q1b1a	Q-L245	59.4	24.7
142386	Germany	Q1b1a	Q-L245	52.5	13.4
195885	Germany	Q1b1a	Q-L245	52.5	13.4
82698	Germany	Q1b1a	Q-L245	52.5	13.4
93226	Germany	Q1b1a	Q-L245	52.5	13.4
N36804	Germany	Q1b1a	Q-L245	52.5	13.4
50021	Hungary	Q1b1a	Q-L245	47.4	19.2
173631	Iran	Q1b1a	Q-L245	35.7	51.4
204938	Iraq	Q1b1a	Q-L245	33.3	44.4
192774	Iraq	Q1b1a	Q-L245	33.3	44.4
197506	Iraq	Q1b1a	Q-L245	33.3	44.4
172691	Ireland	Q1b1a	Q-L245	53.3	6.3
237760	Italy	Q1b1a	Q-L245	41.9	12.5
51935	Latvia	Q1b1a	Q-L245	56.9	24.1
138817	Latvia	Q1b1a	Q-L245	56.9	24.1
N23549	Latvia	Q1b1a	Q-L245	56.9	24.1

80706	Lithuania	Q1b1a	Q-L245	54.7	25.3
115493	Lithuania	Q1b1a	Q-L245	54.7	25.3
1315	Lithuania	Q1b1a	Q-L245	54.7	25.3
184532	Lithuania	Q1b1a	Q-L245	54.7	25.3
N88858	Lithuania	Q1b1a	Q-L245	54.7	25.3
165402	Netherlands	Q1b1a	Q-L245	52.4	4.9
148637	Poland	Q1b1a	Q-L245	52.2	21
172625	Poland	Q1b1a	Q-L245	52.2	21
N60780	Poland	Q1b1a	Q-L245	52.2	21
229377	Poland	Q1b1a	Q-L245	52.2	21
200010	Poland	Q1b1a	Q-L245	52.2	21
N94601	Russia	Q1b1a	Q-L245	55.7	37.6
N102874	Saudi Arabia	Q1b1a	Q-L245	24.6	46.7
N63269	Switzerland	Q1b1a	Q-L245	46.9	7.4
115454	Switzerland	Q1b1a	Q-L245	46.9	7.4
76487	Syria	Q1b1a	Q-L245	33.5	36.3
M7181	Turkey	Q1b1a	Q-L245	39.9	32.9
173902	Turkey	Q1b1a	Q-L245	39.9	32.9
178717	Turkey	Q1b1a	Q-L245	39.9	32.9
45830	Ukraine	Q1b1a	Q-L245	50.4	30.5
196332	Ukraine	Q1b1a	Q-L245	50.4	30.5
117351	Ukraine	Q1b1a	Q-L245	50.4	30.5
45731	Ukraine	Q1b1a	Q-L245	50.4	30.5
N77266	Armenia	R1a1a1*	Z93+ L342.2- L657-	40.2	44.5
202574	Armenia	R1a1a1*	Z93+ L342.2- L657-	40.2	44.5
208164	Italy	R1a1a1*	Z93+ L342.2- L657-	41.9	12.5
217611	Kazakhstan	R1a1a1*	Z93+ L342.2- L657-	51.2	71.4
188931	Kuwait	R1a1a1*	Z93+ L342.2- L657-	29.4	47.9
201431	Kuwait	R1a1a1*	Z93+ L342.2- L657-	29.4	47.9
M6469	Oman	R1a1a1*	Z93+ L342.2- L657-	23.6	58.5
M6630	Oman	R1a1a1*	Z93+ L342.2- L657-	23.6	58.5
48908	Poland	R1a1a1*	Z93+ L342.2- L657-	52.2	21
221419	Russia	R1a1a1*	Z93+ L342.2- L657-	55.7	37.6
189816	Russia	R1a1a1*	Z93+ L342.2- L657-	55.7	37.6
M7858	Saudi Arabia	R1a1a1*	Z93+ L342.2- L657-	24.6	46.7
204324	Saudi Arabia	R1a1a1*	Z93+ L342.2- L657-	24.6	46.7
234858	Saudi Arabia	R1a1a1*	Z93+ L342.2- L657-	24.6	46.7
M6712	Saudi Arabia	R1a1a1*	Z93+ L342.2- L657-	24.6	46.7
M7171	Saudi Arabia	R1a1a1*	Z93+ L342.2- L657-	24.6	46.7
166189	Tunisia	R1a1a1*	Z93+ L342.2- L657-	36.8	10.2
166190	Tunisia	R1a1a1*	Z93+ L342.2- L657-	36.8	10.2
209438	Turkey	R1a1a1*	Z93+ L342.2- L657-	39.9	32.9
222776	Turkey	R1a1a1*	Z93+ L342.2- L657-	39.9	32.9
212823	Turkey	R1a1a1*	Z93+ L342.2- L657-	39.9	32.9
42050	Turkey	R1a1a1*	Z93+ L342.2- L657-	39.9	32.9
182325	UAE	R1a1a1*	Z93+ L342.2- L657-	24.5	54.4
M6507	UAE	R1a1a1*	Z93+ L342.2- L657-	24.5	54.4
167830	UK	R1a1a1*	Z93+ L342.2- L657-	51.5	0.1
226562	UK	R1a1a1*	Z93+ L342.2- L657-	51.5	0.1
127747	UK	R1a1a1*	Z93+ L342.2- L657-	51.5	0.1
N10407	UK	R1a1a1*	Z93+ L342.2- L657-	51.5	0.1
N49878	Afghanistan	R1a1a1h1	L342.2+ L657-	34.5	69.2
192072	Armenia	R1a1a1h1	L342.2+ L657-	40.2	44.5
B1000	Armenia	R1a1a1h1	L342.2+ L657-	40.2	44.5

M6890	Egypt	R1a1a1h1	L342.2+ L657-	30.1	31.2
216686	Georgia	R1a1a1h1	L342.2+ L657-	41.7	44.8
13091	Germany	R1a1a1h1	L342.2+ L657-	52.5	13.4
E11612	Germany	R1a1a1h1	L342.2+ L657-	52.5	13.4
164030	Hungary	R1a1a1h1	L342.2+ L657-	47.4	19.2
197670	India	R1a1a1h1	L342.2+ L657-	21	78
N101746	India	R1a1a1h1	L342.2+ L657-	21	78
N3798	India	R1a1a1h1	L342.2+ L657-	21	78
N77532	India	R1a1a1h1	L342.2+ L657-	21	78
160543	Iraq	R1a1a1h1	L342.2+ L657-	33.3	44.4
H1483	Iraq	R1a1a1h1	L342.2+ L657-	33.3	44.4
214566	Kazakhstan	R1a1a1h1	L342.2+ L657-	51.2	71.4
164862	Kuwait	R1a1a1h1	L342.2+ L657-	29.4	47.9
M6458	Kuwait	R1a1a1h1	L342.2+ L657-	29.4	47.9
M7013	Kuwait	R1a1a1h1	L342.2+ L657-	29.4	47.9
M7432	Kuwait	R1a1a1h1	L342.2+ L657-	29.4	47.9
111287	Kyrgyzstan	R1a1a1h1	L342.2+ L657-	42.9	74.6
116213	Levant	R1a1a1h1	L342.2+ L657-	33.4	35.5
177949	Lithuania	R1a1a1h1	L342.2+ L657-	54.7	25.3
M6469	Oman	R1a1a1h1	L342.2+ L657-	23.6	58.5
181089	Pakistan	R1a1a1h1	L342.2+ L657-	33.7	73.2
160271	Qatar	R1a1a1h1	L342.2+ L657-	25.3	51.5
184336	Qatar	R1a1a1h1	L342.2+ L657-	25.3	51.5
M6285	Qatar	R1a1a1h1	L342.2+ L657-	25.3	51.5
79138	Romania	R1a1a1h1	L342.2+ L657-	44.4	26.1
N97469	Romania	R1a1a1h1	L342.2+ L657-	44.4	26.1
202698	Russia	R1a1a1h1	L342.2+ L657-	55.7	37.6
210272	Russia	R1a1a1h1	L342.2+ L657-	55.7	37.6
M6183	Saudi Arabia	R1a1a1h1	L342.2+ L657-	24.6	46.7
M6698	Saudi Arabia	R1a1a1h1	L342.2+ L657-	24.6	46.7
M6851	Saudi Arabia	R1a1a1h1	L342.2+ L657-	24.6	46.7
M6853	Saudi Arabia	R1a1a1h1	L342.2+ L657-	24.6	46.7
M6982	Saudi Arabia	R1a1a1h1	L342.2+ L657-	24.6	46.7
157103	Saudi Arabia	R1a1a1h1	L342.2+ L657-	24.6	46.7
161602	Saudi Arabia	R1a1a1h1	L342.2+ L657-	24.6	46.7
162855	Saudi Arabia	R1a1a1h1	L342.2+ L657-	24.6	46.7
172811	Saudi Arabia	R1a1a1h1	L342.2+ L657-	24.6	46.7
178906	Saudi Arabia	R1a1a1h1	L342.2+ L657-	24.6	46.7
178907	Saudi Arabia	R1a1a1h1	L342.2+ L657-	24.6	46.7
207608	Saudi Arabia	R1a1a1h1	L342.2+ L657-	24.6	46.7
216270	Saudi Arabia	R1a1a1h1	L342.2+ L657-	24.6	46.7
M6280	Saudi Arabia	R1a1a1h1	L342.2+ L657-	24.6	46.7
M6895	Saudi Arabia	R1a1a1h1	L342.2+ L657-	24.6	46.7
M7199	Saudi Arabia	R1a1a1h1	L342.2+ L657-	24.6	46.7
M6699	*Saudi Arabia*	R1a1a1h1	L342.2+ L657-	24.6	46.7
2914	Syria	R1a1a1h1	L342.2+ L657-	33.5	36.3
158657	Turkey	R1a1a1h1	L342.2+ L657-	39.9	32.9
179005	Turkey	R1a1a1h1	L342.2+ L657-	39.9	32.9
227190	Turkey	R1a1a1h1	L342.2+ L657-	39.9	32.9
167393	UAE	R1a1a1h1	L342.2+ L657-	24.5	54.4
196269	UAE	R1a1a1h1	L342.2+ L657-	24.5	54.4
214554	UAE	R1a1a1h1	L342.2+ L657-	24.5	54.4
M6132	UAE	R1a1a1h1	L342.2+ L657-	24.5	54.4
M6966	UAE	R1a1a1h1	L342.2+ L657-	24.5	54.4

M7001	UAE	R1a1a1h1	L342.2+ L657-	24.5	54.4
B1139	UK	R1a1a1h1	L342.2+ L657-	51.5	0.1
209969	Ukraine	R1a1a1h1	L342.2+ L657-	50.4	30.5
N30333	Ukraine	R1a1a1h1	L342.2+ L657-	50.4	30.5
M7176	Bahrain	R1a1a1h1a	L342.2+ L657+	26.2	50.6
NA20846	India	R1a1a1h1a	L342.2+ L657+	21	78
N78906	India	R1a1a1h1a	L342.2+ L657+	21	78
N12617	India	R1a1a1h1a	L342.2+ L657+	21	78
N76161	India	R1a1a1h1a	L342.2+ L657+	21	78
N2358	India	R1a1a1h1a	L342.2+ L657+	21	78
163483	India	R1a1a1h1a	L342.2+ L657+	21	78
N22414	India	R1a1a1h1a	L342.2+ L657+	21	78
U2321	India	R1a1a1h1a	L342.2+ L657+	21	78
N6841	India	R1a1a1h1a	L342.2+ L657+	21	78
M6736	Iran	R1a1a1h1a	L342.2+ L657+	35.7	51.4
112208	Kazakhstan	R1a1a1h1a	L342.2+ L657+	51.2	71.4
234103	Kuwait	R1a1a1h1a	L342.2+ L657+	29.4	47.9
216619	Pakistan	R1a1a1h1a	L342.2+ L657+	33.7	73.2
N102178	Pakistan	R1a1a1h1a	L342.2+ L657+	33.7	73.2
U2810	Pakistan	R1a1a1h1a	L342.2+ L657+	33.7	73.2
M6308	Qatar	R1a1a1h1a	L342.2+ L657+	25.3	51.5
M7041	Qatar	R1a1a1h1a	L342.2+ L657+	25.3	51.5
158432	Saudi Arabia	R1a1a1h1a	L342.2+ L657+	24.6	46.7
188795	Saudi Arabia	R1a1a1h1a	L342.2+ L657+	24.6	46.7
189737	Saudi Arabia	R1a1a1h1a	L342.2+ L657+	24.6	46.7
189739	Saudi Arabia	R1a1a1h1a	L342.2+ L657+	24.6	46.7
190263	Saudi Arabia	R1a1a1h1a	L342.2+ L657+	24.6	46.7
195320	Saudi Arabia	R1a1a1h1a	L342.2+ L657+	24.6	46.7
240464	Saudi Arabia	R1a1a1h1a	L342.2+ L657+	24.6	46.7
M6740	Saudi Arabia	R1a1a1h1a	L342.2+ L657+	24.6	46.7
M7290	Saudi Arabia	R1a1a1h1a	L342.2+ L657+	24.6	46.7
M7417	Saudi Arabia	R1a1a1h1a	L342.2+ L657+	24.6	46.7
M6303	Saudi Arabia	R1a1a1h1a	L342.2+ L657+	24.6	46.7
74750	Turkey	R1a1a1h1a	L342.2+ L657+	39.9	32.9
M6646	UAE	R1a1a1h1a	L342.2+ L657+	24.5	54.4
M6986	UAE	R1a1a1h1a	L342.2+ L657+	24.5	54.4
M7443	UAE	R1a1a1h1a	L342.2+ L657+	24.5	54.4
E12439	Albania	R1b1a2a1a	R-L150+	41.3	19.8
128958	Armenia	R1b1a2a1a	R-L150+	40.2	44.5
176676	Armenia	R1b1a2a1a	R-L150+	40.2	44.5
207694	Armenia	R1b1a2a1a	R-L150+	40.2	44.5
164210	Armenia	R1b1a2a1a	R-L150+	40.2	44.5
152974	Armenia	R1b1a2a1a	R-L150+	40.2	44.5
195750	Armenia	R1b1a2a1a	R-L150+	40.2	44.5
149197	Armenia	R1b1a2a1a	R-L150+	40.2	44.5
152975	Armenia	R1b1a2a1a	R-L150+	40.2	44.5
149196	Armenia	R1b1a2a1a	R-L150+	40.2	44.5
152977	Armenia	R1b1a2a1a	R-L150+	40.2	44.5
189660	Bulgaria	R1b1a2a1a	R-L150+	42.7	23.3
118282	Croatia	R1b1a2a1a	R-L150+	45.8	16
54168	Czech Republic	R1b1a2a1a	R-L150+	50.1	14.5
N70876	Denmark	R1b1a2a1a	R-L150+	55.7	12.6
102551	France	R1b1a2a1a	R-L150+	48.9	2.4
8U3YQ	Germany	R1b1a2a1a	R-L150+	52.5	13.4

140135	Germany	R1b1a2a1a	R-L150+	52.5	13.4
161457	Germany	R1b1a2a1a	R-L150+	52.5	13.4
7PJJJ	Germany	R1b1a2a1a	R-L150+	52.5	13.4
91989	Germany	R1b1a2a1a	R-L150+	52.5	13.4
87265	Greece	R1b1a2a1a	R-L150+	37.9	23.7
162445	Greece	R1b1a2a1a	R-L150+	37.9	23.7
191402	Greece	R1b1a2a1a	R-L150+	37.9	23.7
121597	Hungary	R1b1a2a1a	R-L150+	47.4	19.2
N83705	Iraq	R1b1a2a1a	R-L150+	33.3	44.4
148406	Ireland	R1b1a2a1a	R-L150+	53.3	6.3
178343	Ireland	R1b1a2a1a	R-L150+	53.3	6.3
5811	Ireland	R1b1a2a1a	R-L150+	53.3	6.3
209781	Ireland	R1b1a2a1a	R-L150+	53.3	6.3
212712	Italy	R1b1a2a1a	R-L150+	41.9	12.5
30885	Italy	R1b1a2a1a	R-L150+	41.9	12.5
E5276	Italy	R1b1a2a1a	R-L150+	41.9	12.5
95875	Italy	R1b1a2a1a	R-L150+	41.9	12.5
223828	Kazakhstan	R1b1a2a1a	R-L150+	51.2	71.4
N97723	Levant	R1b1a2a1a	R-L150+	33.4	35.5
131176	Levant	R1b1a2a1a	R-L150+	33.4	35.5
66841	Lithuania	R1b1a2a1a	R-L150+	54.7	25.3
159189	Netherlands	R1b1a2a1a	R-L150+	52.4	4.9
202070	Pakistan	R1b1a2a1a	R-L150+	33.7	73.2
92187	Poland	R1b1a2a1a	R-L150+	52.2	21
N36978	Poland	R1b1a2a1a	R-L150+	52.2	21
9-21793	Poland	R1b1a2a1a	R-L150+	52.2	21
N68231	Poland	R1b1a2a1a	R-L150+	52.2	21
202362	Portugal	R1b1a2a1a	R-L150+	38.8	-9.1
198311	Russia	R1b1a2a1a	R-L150+	55.7	37.6
159888	Russia	R1b1a2a1a	R-L150+	55.7	37.6
152880	Russia	R1b1a2a1a	R-L150+	55.7	37.6
N51668	Spain	R1b1a2a1a	R-L150+	40.4	-3.7
6UQDH	Spain	R1b1a2a1a	R-L150+	40.4	-3.7
177152	Switzerland	R1b1a2a1a	R-L150+	46.9	7.4
E5182	Switzerland	R1b1a2a1a	R-L150+	46.9	7.4
70052	Syria	R1b1a2a1a	R-L150+	33.5	36.3
162059	Turkey	R1b1a2a1a	R-L150+	39.9	32.9
164229	Turkey	R1b1a2a1a	R-L150+	39.9	32.9
N93606	Turkey	R1b1a2a1a	R-L150+	39.9	32.9
185690	Turkey	R1b1a2a1a	R-L150+	39.9	32.9
172019	Turkey	R1b1a2a1a	R-L150+	39.9	32.9
185783	Turkey	R1b1a2a1a	R-L150+	39.9	32.9
M6266	UAE	R1b1a2a1a	R-L150+	24.5	54.4
234946	UK	R1b1a2a1a	R-L150+	51.5	0.1
B1388	UK	R1b1a2a1a	R-L150+	51.5	0.1
72584	UK	R1b1a2a1a	R-L150+	51.5	0.1
35045	UK	R1b1a2a1a	R-L150+	51.5	0.1
26483	UK	R1b1a2a1a	R-L150+	51.5	0.1
196157	UK	R1b1a2a1a	R-L150+	51.5	0.1
3-10470	UK	R1b1a2a1a	R-L150+	51.5	0.1
34372	UK	R1b1a2a1a	R-L150+	51.5	0.1
82745	UK	R1b1a2a1a	R-L150+	51.5	0.1
16910	UK	R1b1a2a1a	R-L150+	51.5	0.1
194313	UK	R1b1a2a1a	R-L150+	51.5	0.1

206335	UK	R1b1a2a1a	R-L150+	51.5	0.1
95535	UK	R1b1a2a1a	R-L150+	51.5	0.1
43759	UK	R1b1a2a1a	R-L150+	51.5	0.1
235098	Ukraine	R1b1a2a1a	R-L150+	50.4	30.5
190448	Ukraine	R1b1a2a1a	R-L150+	50.4	30.5
155694	Armenia	J2a*	J-M410+ L26- M340- P279-	40.2	44.5
194776	Armenia	J2a*	J-M410+ L26- M340- P279-	40.2	44.5
168300	Austria	J2a*	J-M410+ L26- M340- P279-	48.2	16.4
198791	Belarus	J2a*	J-M410+ L26- M340- P279-	53.9	27.6
32523	France	J2a*	J-M410+ L26- M340- P279-	48.9	2.4
N86999	France	J2a*	J-M410+ L26- M340- P279-	48.9	2.4
E10625	Georgia	J2a*	J-M410+ L26- M340- P279-	41.7	44.8
226181	Germany	J2a*	J-M410+ L26- M340- P279-	52.5	13.4
128474	Germany	J2a*	J-M410+ L26- M340- P279-	52.5	13.4
107132	Germany	J2a*	J-M410+ L26- M340- P279-	52.5	13.4
E8073	Italy	J2a*	J-M410+ L26- M340- P279-	41.9	12.5
143591	Italy	J2a*	J-M410+ L26- M340- P279-	41.9	12.5
96954	Italy	J2a*	J-M410+ L26- M340- P279-	41.9	12.5
95743	Latvia	J2a*	J-M410+ L26- M340- P279-	56.9	24.1
163162	Lebanon	J2a*	J-M410+ L26- M340- P279-	33.4	35.5
M6058	Libya	J2a*	J-M410+ L26- M340- P279-	32.9	13.2
135080	Lithuania	J2a*	J-M410+ L26- M340- P279-	54.7	25.3
N14134	Lithuania	J2a*	J-M410+ L26- M340- P279-	54.7	25.3
155891	Ossetia	J2a*	J-M410+ L26- M340- P279-	41.7	44.8
E4882	Poland	J2a*	J-M410+ L26- M340- P279-	52.2	21
210028	Poland	J2a*	J-M410+ L26- M340- P279-	52.2	21
N40835	Russia	J2a*	J-M410+ L26- M340- P279-	55.7	37.6
119738	Russia	J2a*	J-M410+ L26- M340- P279-	55.7	37.6
127744	Russia	J2a*	J-M410+ L26- M340- P279-	55.7	37.6
216541	Saudi Arabia	J2a*	J-M410+ L26- M340- P279-	24.6	46.7
233412	Spain	J2a*	J-M410+ L26- M340- P279-	40.4	-3.7
196834	Ukraine	J2a*	J-M410+ L26- M340- P279-	50.4	30.5
N79407	Ukraine	J2a*	J-M410+ L26- M340- P279-	50.4	30.5
79325	Czech Republic	R1a-L260	R-M458+ L260+	50.1	14.5
12044	Czech Republic	R1a-L260	R-M458+ L260+	50.1	14.5
E9693	Czech Republic	R1a-L260	R-M458+ L260+	50.1	14.5
26769	Czech Republic	R1a-L260	R-M458+ L260+	50.1	14.5
E6057	Germany	R1a-L260	R-M458+ L260+	52.5	13.4
162431	Germany	R1a-L260	R-M458+ L260+	52.5	13.4
118487	Hungary	R1a-L260	R-M458+ L260+	47.4	19.2
100432	Poland	R1a-L260	R-M458+ L260+	52.2	21
E11471	Poland	R1a-L260	R-M458+ L260+	52.2	21
N93969	Poland	R1a-L260	R-M458+ L260+	52.2	21
194010	Poland	R1a-L260	R-M458+ L260+	52.2	21
116518	Poland	R1a-L260	R-M458+ L260+	52.2	21
125922	Poland	R1a-L260	R-M458+ L260+	52.2	21
N30169	Poland	R1a-L260	R-M458+ L260+	52.2	21
85265	Poland	R1a-L260	R-M458+ L260+	52.2	21
4623	Poland	R1a-L260	R-M458+ L260+	52.2	21
22620	Poland	R1a-L260	R-M458+ L260+	52.2	21
N3662	Poland	R1a-L260	R-M458+ L260+	52.2	21
11992	Poland	R1a-L260	R-M458+ L260+	52.2	21
46336	Poland	R1a-L260	R-M458+ L260+	52.2	21
N18946	Poland	R1a-L260	R-M458+ L260+	52.2	21

172283	Poland	R1a-L260	R-M458+ L260+	52.2	21
179688	Poland	R1a-L260	R-M458+ L260+	52.2	21
209544	Poland	R1a-L260	R-M458+ L260+	52.2	21
94796	Poland	R1a-L260	R-M458+ L260+	52.2	21
86790	Poland	R1a-L260	R-M458+ L260+	52.2	21
N13715	Poland	R1a-L260	R-M458+ L260+	52.2	21
152662	Russia	R1a-L260	R-M458+ L260+	55.7	37.6
31553	Russia	R1a-L260	R-M458+ L260+	55.7	37.6
185639	Slovakia	R1a-L260	R-M458+ L260+	48.2	17.1
67728	Slovakia	R1a-L260	R-M458+ L260+	48.2	17.1
31257	Ukraine	R1a-L260	R-M458+ L260+	50.4	30.5
137778	Germany	R1a-L365+	R-Z280+ L365+	52.5	13.4
71501	Germany	R1a-L365+	R-Z280+ L365+	52.5	13.4
N7393	Germany	R1a-L365+	R-Z280+ L365+	52.5	13.4
88309	Germany	R1a-L365+	R-Z280+ L365+	52.5	13.4
N74814	Poland	R1a-L365+	R-Z280+ L365+	52.2	21
165792	Poland	R1a-L365+	R-Z280+ L365+	52.2	21
133745	Poland	R1a-L365+	R-Z280+ L365+	52.2	21
57685	Poland	R1a-L365+	R-Z280+ L365+	52.2	21
E10118	Poland	R1a-L365+	R-Z280+ L365+	52.2	21
E9666	Poland	R1a-L365+	R-Z280+ L365+	52.2	21
172020	Poland	R1a-L365+	R-Z280+ L365+	52.2	21
23292	Poland	R1a-L365+	R-Z280+ L365+	52.2	21
N17352	Poland	R1a-L365+	R-Z280+ L365+	52.2	21
N2335	Poland	R1a-L365+	R-Z280+ L365+	52.2	21
23291	Poland	R1a-L365+	R-Z280+ L365+	52.2	21
12624	Poland	R1a-L365+	R-Z280+ L365+	52.2	21
N30766	Ukraine	R1a-L365+	R-Z280+ L365+	50.4	30.5
7295	Ukraine	R1a-L365+	R-Z280+ L365+	50.4	30.5
N49600	Germany	R1a1-Z283*	R-Z283+ Z280- M458- Z284-	52.5	13.4
160159	Germany	R1a1-Z283*	R-Z283+ Z280- M458- Z284-	52.5	13.4
227082	Germany	R1a1-Z283*	R-Z283+ Z280- M458- Z284-	52.5	13.4
201431	Kuwait	R1a1-Z283*	R-Z283+ Z280- M458- Z284-	29.4	47.9
132838	Poland	R1a1-Z283*	R-Z283+ Z280- M458- Z284-	52.2	21
44591	Poland	R1a1-Z283*	R-Z283+ Z280- M458- Z284-	52.2	21
M7258	Saudi Arabia	R1a1-Z283*	R-Z283+ Z280- M458- Z284-	24.6	46.7
232376	Saudi Arabia	R1a1-Z283*	R-Z283+ Z280- M458- Z284-	24.6	46.7
231554	Saudi Arabia	R1a1-Z283*	R-Z283+ Z280- M458- Z284-	24.6	46.7
131765	Switzerland	R1a1-Z283*	R-Z283+ Z280- M458- Z284-	46.9	7.4
214352	Turkey	R1a1-Z283*	R-Z283+ Z280- M458- Z284-	39.9	32.9
182305	Turkey	R1a1-Z283*	R-Z283+ Z280- M458- Z284-	39.9	32.9
N50360	UK	R1a1-Z283*	R-Z283+ Z280- M458- Z284-	51.5	0.1
198114	UK	R1a1-Z283*	R-Z283+ Z280- M458- Z284-	51.5	0.1
18130	UK	R1a1-Z283*	R-Z283+ Z280- M458- Z284-	51.5	0.1
166219	UK	R1a1-Z283*	R-Z283+ Z280- M458- Z284-	51.5	0.1
187138	Ukraine	R1a1-Z283*	R-Z283+ Z280- M458- Z284-	50.4	30.5
107ASM12	Afghanistan	Q1b*Q1b1*	Q-L275+ L245-	34.5	69.2
N58043	India	Q1b*Q1b1*	Q-L275+ L245-	21	78
N19260	India	Q1b*Q1b1*	Q-L275+ L245-	21	78
037ASM10	India	Q1b*Q1b1*	Q-L275+ L245-	21	78
067ASM10	India	Q1b*Q1b1*	Q-L275+ L245-	21	78
N78873	India	Q1b*Q1b1*	Q-L275+ L245-	21	78
N39405	India	Q1b*Q1b1*	Q-L275+ L245-	21	78
193005	Kazakhstan	Q1b*Q1b1*	Q-L275+ L245-	51.2	71.4

210883	Kazakhstan	Q1b*Q1b1*	Q-L275+ L245-	51.2	71.4
224482	Pakistan	Q1b*Q1b1*	Q-L275+ L245-	33.7	73.2
N29052	Pakistan	Q1b*Q1b1*	Q-L275+ L245-	33.7	73.2
N14041	Pakistan	Q1b*Q1b1*	Q-L275+ L245-	33.7	73.2
53682	Poland	Q1b*Q1b1*	Q-L275+ L245-	52.2	21
13254	Portugal	Q1b*Q1b1*	Q-L275+ L245-	38.8	-9.1
187028	Germany	Q1b*Q1b1*	Q-L275+ L245-	52.5	13.4
181179	Russia	Q1b*Q1b1*	Q-L275+ L245-	55.7	37.6
182771	Russia	Q1b*Q1b1*	Q-L275+ L245-	55.7	37.6
182773	Russia	Q1b*Q1b1*	Q-L275+ L245-	55.7	37.6
26360	Uzbekhistan	Q1b*Q1b1*	Q-L275+ L245-	41.3	69.2
174248	Armenia	J2a4b	J-M67+ L210-	40.2	44.5
174250	Armenia	J2a4b	J-M67+ L210-	40.2	44.5
176688	Armenia	J2a4b	J-M67+ L210-	40.2	44.5
216529	Georgia	J2a4b	J-M67+ L210-	41.7	44.8
31852	Germany	J2a4b	J-M67+ L210-	52.5	13.4
139708	Germany	J2a4b	J-M67+ L210-	52.5	13.4
168536	Germany	J2a4b	J-M67+ L210-	52.5	13.4
70513	Iraq	J2a4b	J-M67+ L210-	33.3	44.4
B1300	Ireland	J2a4b	J-M67+ L210-	53.3	6.3
149533	Israel	J2a4b	J-M67+ L210-	33.4	35.5
18790	Italy	J2a4b	J-M67+ L210-	41.9	12.5
N89155	Italy	J2a4b	J-M67+ L210-	41.9	12.5
103307	Netherlands	J2a4b	J-M67+ L210-	52.4	4.9
159331	Portugal	J2a4b	J-M67+ L210-	38.8	-9.1
230305	Russia	J2a4b	J-M67+ L210-	55.7	37.6
N3620	Sudan	J2a4b	J-M67+ L210-	15.6	32.5
198821	Syria	J2a4b	J-M67+ L210-	33.5	36.3
164196	Turkey	J2a4b	J-M67+ L210-	39.9	32.9
182262	UK	J2a4b	J-M67+ L210-	51.5	0.1
N38534	UK	J2a4b	J-M67+ L210-	51.5	0.1
N93584	UK	J2a4b	J-M67+ L210-	51.5	0.1
166319	Armenia	R1b1*	R-P25+ P297- M335- V88-	40.2	44.5
181545	Armenia	R1b1*	R-P25+ P297- M335- V88-	40.2	44.5
165317	Bahrain	R1b1*	R-P25+ P297- M335- V88-	26.2	50.6
N16605	Belarus	R1b1*	R-P25+ P297- M335- V88-	53.9	27.6
216826	Georgia	R1b1*	R-P25+ P297- M335- V88-	41.7	44.8
112962	Germany	R1b1*	R-P25+ P297- M335- V88-	52.5	13.4
97835	Hungary	R1b1*	R-P25+ P297- M335- V88-	47.4	19.2
N93357	India	R1b1*	R-P25+ P297- M335- V88-	21	78
N39156	Iraq	R1b1*	R-P25+ P297- M335- V88-	33.3	44.4
N83832	Italy	R1b1*	R-P25+ P297- M335- V88-	41.9	12.5
34520	Lithuania	R1b1*	R-P25+ P297- M335- V88-	54.7	25.3
N4337	Poland	R1b1*	R-P25+ P297- M335- V88-	52.2	21
N2813	Poland	R1b1*	R-P25+ P297- M335- V88-	52.2	21
110387	Russia	R1b1*	R-P25+ P297- M335- V88-	55.7	37.6
170336	Saudi Arabia	R1b1	R-P25+ P297- M335- V88-	24.6	46.7
160040	Saudi Arabia	R1b1	R-P25+ P297- M335- V88-	24.6	46.7
48742	Spain	R1b1*	R-P25+ P297- M335- V88-	40.4	-3.7
N26020	Spain	R1b1*	R-P25+ P297- M335- V88-	40.4	-3.7
161210	Spain	R1b1*	R-P25+ P297- M335- V88-	40.4	-3.7
149191	Turkey	R1b1*	R-P25+ P297- M335- V88-	39.9	32.9
2372	Turkey	R1b1*	R-P25+ P297- M335- V88-	39.9	32.9
150748	UK	R1b1*	R-P25+ P297- M335- V88-	51.5	0.1

57176	Ukraine	R1b1*	R-P25+ P297- M335- V88-	50.4	30.5
262	Ukraine	R1b1*	R-P25+ P297- M335- V88-	50.4	30.5
189402	Uzbekhistan	R1b1*	R-P25+ P297- M335- V88-	41.3	69.2
134423	Armenia	R1b1a2*	R-M269+ L23-	40.2	44.5
101029	Belarus	R1b1a2*	R-M269+ L23-	53.9	27.6
163904	Belarus	R1b1a2*	R-M269+ L23-	53.9	27.6
N60445	France	R1b1a2*	R-M269+ L23-	48.9	2.4
N4522	Germany	R1b1a2*	R-M269+ L23-	52.5	13.4
QXGKN	Italy	R1b1a2*	R-M269+ L23-	41.9	12.5
5VU5V	Italy	R1b1a2*	R-M269+ L23-	41.9	12.5
46835	Italy	R1b1a2*	R-M269+ L23-	41.9	12.5
170870	Poland	R1b1a2*	R-M269+ L23-	52.2	21
169223	Syria	R1b1a2*	R-M269+ L23-	33.5	36.3
131645	Turkey	R1b1a2*	R-M269+ L23-	39.9	32.9
174259	Turkey	R1b1a2*	R-M269+ L23-	39.9	32.9
2146	Ukraine	R1b1a2*	R-M269+ L23-	50.4	30.5
158476	Ukraine	R1b1a2*	R-M269+ L23-	50.4	30.5
126775	Ukraine	R1b1a2*	R-M269+ L23-	50.4	30.5
177512	UK	R1b1a2*	R-M269+ L23-	51.5	0.1
113425	Levant	R1b1a2*	R-M269+ L23-	52.2	21
195750	Armenia	R1b1a2a1*	R-L150+ L51-	40.2	44.5
149196	Armenia	R1b1a2a1*	R-L150+ L51-	40.2	44.5
184381	Armenia	R1b1a2a1*	R-L150+ L51-	40.2	44.5
54168	Czech Republic	R1b1a2a1*	R-L150+ L51-	50.1	14.5
N76689	Egypt	R1b1a2a1*	R-L150+ L51-	30.1	31.2
102551	France	R1b1a2a1*	R-L150+ L51-	48.9	2.4
7PJJJ	Germany	R1b1a2a1*	R-L150+ L51-	52.5	13.4
121597	Hungary	R1b1a2a1*	R-L150+ L51-	47.4	19.2
5811	Ireland	R1b1a2a1*	R-L150+ L51-	53.3	6.3
30885	Italy	R1b1a2a1*	R-L150+ L51-	41.9	12.5
E5276	Italy	R1b1a2a1*	R-L150+ L51-	41.9	12.5
95875	Italy	R1b1a2a1*	R-L150+ L51-	41.9	12.5
223828	Kazakhstan	R1b1a2a1*	R-L150+ L51-	51.2	71.4
131176	Lebanon	R1b1a2a1*	R-L150+ L51-	33.4	35.5
N97723	Lebanon	R1b1a2a1*	R-L150+ L51-	33.4	35.5
66841	Lithuania	R1b1a2a1*	R-L150+ L51-	54.7	25.3
N68231	Poland	R1b1a2a1*	R-L150+ L51-	52.2	21
166563	Poland	R1b1a2a1*	R-L150+ L51-	52.2	21
92187	Poland	R1b1a2a1*	R-L150+ L51-	52.2	21
N36978	Poland	R1b1a2a1*	R-L150+ L51-	52.2	21
9-21793	Poland	R1b1a2a1*	R-L150+ L51-	52.2	21
N3622	Spain	R1b1a2a1*	R-L150+ L51-	40.4	-3.7
162059	Turkey	R1b1a2a1*	R-L150+ L51-	39.9	32.9
185783	Turkey	R1b1a2a1*	R-L150+ L51-	39.9	32.9
164229	Turkey	R1b1a2a1*	R-L150+ L51-	39.9	32.9
34372	UK	R1b1a2a1*	R-L150+ L51-	51.5	0.1
82745	UK	R1b1a2a1*	R-L150+ L51-	51.5	0.1
26483	UK	R1b1a2a1*	R-L150+ L51-	51.5	0.1
194313	UK	R1b1a2a1*	R-L150+ L51-	51.5	0.1
95535	UK	R1b1a2a1*	R-L150+ L51-	51.5	0.1
16910	UK	R1b1a2a1*	R-L150+ L51-	51.5	0.1
64409	UK	R1b1a2a1*	R-L150+ L51-	51.5	0.1
W63WR	Croatia	R1b1a2a1a*	R-L51+ L11-	45.8	16
87-5845	Germany	R1b1a2a1a*	R-L51+ L11-	52.5	13.4

134706	Ireland	R1b1a2a1a*	R-L51+ L11-	53.3	6.3
N5273	Italy	R1b1a2a1a*	R-L51+ L11-	41.9	12.5
143613	Netherlands	R1b1a2a1a*	R-L51+ L11-	52.4	4.9
80001	Poland	R1b1a2a1a*	R-L51+ L11-	52.2	21
80593	Poland	R1b1a2a1a*	R-L51+ L11-	52.2	21
E2689	Spain	R1b1a2a1a*	R-L51+ L11-	40.4	-3.7
46468	Turkey	R1b1a2a1a*	R-L51+ L11-	39.9	32.9
132478	UK	R1b1a2a1a*	R-L51+ L11-	51.5	0.1
49624	UK	R1b1a2a1a*	R-L51+ L11-	51.5	0.1
111988	UK	R1b1a2a1a*	R-L51+ L11-	51.5	0.1
97317	UK	R1b1a2a1a*	R-L51+ L11-	51.5	0.1
41519	UK	R1b1a2a1a*	R-L51+ L11-	51.5	0.1
TAC7A	Armenia	R1b1a2a1a1*	R-L11+ U106-	40.2	44.5
UAWPH	France	R1b1a2a1a1*	R-L11+ U106-	48.9	2.4
110855	France	R1b1a2a1a1*	R-L11+ U106-	48.9	2.4
N58749	France	R1b1a2a1a1*	R-L11+ U106-	48.9	2.4
58927	France	R1b1a2a1a1*	R-L11+ U106-	48.9	2.4
66573	France	R1b1a2a1a1*	R-L11+ U106-	48.9	2.4
67632	France	R1b1a2a1a1*	R-L11+ U106-	48.9	2.4
82681	France	R1b1a2a1a1*	R-L11+ U106-	48.9	2.4
99994	France	R1b1a2a1a1*	R-L11+ U106-	48.9	2.4
86866	France	R1b1a2a1a1*	R-L11+ U106-	48.9	2.4
N36241	France	R1b1a2a1a1*	R-L11+ U106-	48.9	2.4
104128	Germany	R1b1a2a1a1*	R-L11+ U106-	52.5	13.4
N2197	Germany	R1b1a2a1a1*	R-L11+ U106-	52.5	13.4
35677	Germany	R1b1a2a1a1*	R-L11+ U106-	52.5	13.4
52652	Germany	R1b1a2a1a1*	R-L11+ U106-	52.5	13.4
P4XCW	Germany	R1b1a2a1a1*	R-L11+ U106-	52.5	13.4
N23935	Germany	R1b1a2a1a1*	R-L11+ U106-	52.5	13.4
N42297	Germany	R1b1a2a1a1*	R-L11+ U106-	52.5	13.4
N21533	Germany	R1b1a2a1a1*	R-L11+ U106-	52.5	13.4
N45541	Germany	R1b1a2a1a1*	R-L11+ U106-	52.5	13.4
N56105	Ireland	R1b1a2a1a1*	R-L11+ U106-	53.3	6.3
47703	Ireland	R1b1a2a1a1*	R-L11+ U106-	53.3	6.3
23171	Ireland	R1b1a2a1a1*	R-L11+ U106-	53.3	6.3
7565	Ireland	R1b1a2a1a1*	R-L11+ U106-	53.3	6.3
81633	Ireland	R1b1a2a1a1*	R-L11+ U106-	53.3	6.3
N41701	Ireland	R1b1a2a1a1*	R-L11+ U106-	53.3	6.3
N21843	Ireland	R1b1a2a1a1*	R-L11+ U106-	53.3	6.3
42273	Ireland	R1b1a2a1a1*	R-L11+ U106-	53.3	6.3
51491	Ireland	R1b1a2a1a1*	R-L11+ U106-	53.3	6.3
101204	Ireland	R1b1a2a1a1*	R-L11+ U106-	53.3	6.3
72665	Ireland	R1b1a2a1a1*	R-L11+ U106-	53.3	6.3
84782	Ireland	R1b1a2a1a1*	R-L11+ U106-	53.3	6.3
4PDJ4	Ireland	R1b1a2a1a1*	R-L11+ U106-	53.3	6.3
63879	Ireland	R1b1a2a1a1*	R-L11+ U106-	53.3	6.3
N8583	Ireland	R1b1a2a1a1*	R-L11+ U106-	53.3	6.3
21482	Ireland	R1b1a2a1a1*	R-L11+ U106-	53.3	6.3
27818	Ireland	R1b1a2a1a1*	R-L11+ U106-	53.3	6.3
47113	Ireland	R1b1a2a1a1*	R-L11+ U106-	53.3	6.3
78183	Ireland	R1b1a2a1a1*	R-L11+ U106-	53.3	6.3
45013	Ireland	R1b1a2a1a1*	R-L11+ U106-	53.3	6.3
27539	Ireland	R1b1a2a1a1*	R-L11+ U106-	53.3	6.3
B38TQ	Ireland	R1b1a2a1a1*	R-L11+ U106-	53.3	6.3

2831	Lithuania	R1b1a2a1a1*	R-L11+ U106-	54.7	25.3
N28819	Netherlands	R1b1a2a1a1*	R-L11+ U106-	52.4	4.9
N16822	Netherlands	R1b1a2a1a1*	R-L11+ U106-	52.4	4.9
6324	Netherlands	R1b1a2a1a1*	R-L11+ U106-	52.4	4.9
85208	Netherlands	R1b1a2a1a1*	R-L11+ U106-	52.4	4.9
4UGEK	Poland	R1b1a2a1a1*	R-L11+ U106-	52.2	21
AKBS5	Poland	R1b1a2a1a1*	R-L11+ U106-	52.2	21
9UNCU	Portugal	R1b1a2a1a1*	R-L11+ U106-	38.8	-9.1
N57279	Portugal	R1b1a2a1a1*	R-L11+ U106-	38.8	-9.1
76021	Spain	R1b1a2a1a1*	R-L11+ U106-	40.4	-3.7
78029	Spain	R1b1a2a1a1*	R-L11+ U106-	40.4	-3.7
N44991	Spain	R1b1a2a1a1*	R-L11+ U106-	40.4	-3.7
46334	Spain	R1b1a2a1a1*	R-L11+ U106-	40.4	-3.7
N7RUW	Switzerland	R1b1a2a1a1*	R-L11+ U106-	46.9	7.4
30635	UK	R1b1a2a1a1*	R-L11+ U106-	51.5	0.1
37068	UK	R1b1a2a1a1*	R-L11+ U106-	51.5	0.1
102517	UK	R1b1a2a1a1*	R-L11+ U106-	51.5	0.1
51794	UK	R1b1a2a1a1*	R-L11+ U106-	51.5	0.1
N48878	UK	R1b1a2a1a1*	R-L11+ U106-	51.5	0.1
26778	UK	R1b1a2a1a1*	R-L11+ U106-	51.5	0.1
50278	UK	R1b1a2a1a1*	R-L11+ U106-	51.5	0.1
84267	UK	R1b1a2a1a1*	R-L11+ U106-	51.5	0.1
4W2GA	UK	R1b1a2a1a1*	R-L11+ U106-	51.5	0.1
S3THJ	UK	R1b1a2a1a1*	R-L11+ U106-	51.5	0.1
N11370	UK	R1b1a2a1a1*	R-L11+ U106-	51.5	0.1
YNWV9	UK	R1b1a2a1a1*	R-L11+ U106-	51.5	0.1
CYQ4N	UK	R1b1a2a1a1*	R-L11+ U106-	51.5	0.1
58796	UK	R1b1a2a1a1*	R-L11+ U106-	51.5	0.1
N1848	UK	R1b1a2a1a1*	R-L11+ U106-	51.5	0.1
15490	UK	R1b1a2a1a1*	R-L11+ U106-	51.5	0.1
31137	UK	R1b1a2a1a1*	R-L11+ U106-	51.5	0.1
16918	UK	R1b1a2a1a1*	R-L11+ U106-	51.5	0.1
65860	UK	R1b1a2a1a1*	R-L11+ U106-	51.5	0.1
66478	UK	R1b1a2a1a1*	R-L11+ U106-	51.5	0.1
88684	UK	R1b1a2a1a1*	R-L11+ U106-	51.5	0.1
3CR7S	UK	R1b1a2a1a1*	R-L11+ U106-	51.5	0.1
49637	UK	R1b1a2a1a1*	R-L11+ U106-	51.5	0.1
N18664	UK	R1b1a2a1a1*	R-L11+ U106-	51.5	0.1
BYFNX	UK	R1b1a2a1a1*	R-L11+ U106-	51.5	0.1
7467	UK	R1b1a2a1a1*	R-L11+ U106-	51.5	0.1
28920	UK	R1b1a2a1a1*	R-L11+ U106-	51.5	0.1
38087	UK	R1b1a2a1a1*	R-L11+ U106-	51.5	0.1
79343	UK	R1b1a2a1a1*	R-L11+ U106-	51.5	0.1
N23712	UK	R1b1a2a1a1*	R-L11+ U106-	51.5	0.1
38806	UK	R1b1a2a1a1*	R-L11+ U106-	51.5	0.1
87278	UK	R1b1a2a1a1*	R-L11+ U106-	51.5	0.1
31131	UK	R1b1a2a1a1*	R-L11+ U106-	51.5	0.1
KZ8MZ	UK	R1b1a2a1a1*	R-L11+ U106-	51.5	0.1
54945	UK	R1b1a2a1a1*	R-L11+ U106-	51.5	0.1
N19218	UK	R1b1a2a1a1*	R-L11+ U106-	51.5	0.1
36308	UK	R1b1a2a1a1*	R-L11+ U106-	51.5	0.1
N8721	UK	R1b1a2a1a1*	R-L11+ U106-	51.5	0.1
N1851	UK	R1b1a2a1a1*	R-L11+ U106-	51.5	0.1
WSUYD	UK	R1b1a2a1a1*	R-L11+ U106-	51.5	0.1

41613	UK	R1b1a2a1a1*	R-L11+ U106-	51.5	0.1
72MZ2	UK	R1b1a2a1a1*	R-L11+ U106-	51.5	0.1
N22147	UK	R1b1a2a1a1*	R-L11+ U106-	51.5	0.1
7AQZG	UK	R1b1a2a1a1*	R-L11+ U106-	51.5	0.1
73824	UK	R1b1a2a1a1*	R-L11+ U106-	51.5	0.1
60472	UK	R1b1a2a1a1*	R-L11+ U106-	51.5	0.1
N3790	UK	R1b1a2a1a1*	R-L11+ U106-	51.5	0.1
38223	UK	R1b1a2a1a1*	R-L11+ U106-	51.5	0.1
59275	UK	R1b1a2a1a1*	R-L11+ U106-	51.5	0.1
39244	UK	R1b1a2a1a1*	R-L11+ U106-	51.5	0.1
47418	UK	R1b1a2a1a1*	R-L11+ U106-	51.5	0.1
2340	UK	R1b1a2a1a1*	R-L11+ U106-	51.5	0.1
N12360	UK	R1b1a2a1a1*	R-L11+ U106-	51.5	0.1
102770	UK	R1b1a2a1a1*	R-L11+ U106-	51.5	0.1
101748	UK	R1b1a2a1a1*	R-L11+ U106-	51.5	0.1
26660	UK	R1b1a2a1a1*	R-L11+ U106-	51.5	0.1
7133	UK	R1b1a2a1a1*	R-L11+ U106-	51.5	0.1
N17878	UK	R1b1a2a1a1*	R-L11+ U106-	51.5	0.1
MFECP	UK	R1b1a2a1a1*	R-L11+ U106-	51.5	0.1
90970	UK	R1b1a2a1a1*	R-L11+ U106-	51.5	0.1
32191	UK	R1b1a2a1a1*	R-L11+ U106-	51.5	0.1
WZDQY	UK	R1b1a2a1a1*	R-L11+ U106-	51.5	0.1
N22536	UK	R1b1a2a1a1*	R-L11+ U106-	51.5	0.1
65194	UK	R1b1a2a1a1*	R-L11+ U106-	51.5	0.1
77772	UK	R1b1a2a1a1*	R-L11+ U106-	51.5	0.1
N3GH8	UK	R1b1a2a1a1*	R-L11+ U106-	51.5	0.1
M4H58	UK	R1b1a2a1a1*	R-L11+ U106-	51.5	0.1
4H9SS	UK	R1b1a2a1a1*	R-L11+ U106-	51.5	0.1
N8983	UK	R1b1a2a1a1*	R-L11+ U106-	51.5	0.1
21572	UK	R1b1a2a1a1*	R-L11+ U106-	51.5	0.1
37094	UK	R1b1a2a1a1*	R-L11+ U106-	51.5	0.1
86680	UK	R1b1a2a1a1*	R-L11+ U106-	51.5	0.1
86930	UK	R1b1a2a1a1*	R-L11+ U106-	51.5	0.1
N3983	UK	R1b1a2a1a1*	R-L11+ U106-	51.5	0.1
2132	UK	R1b1a2a1a1*	R-L11+ U106-	51.5	0.1
82157	UK	R1b1a2a1a1*	R-L11+ U106-	51.5	0.1
12300	UK	R1b1a2a1a1*	R-L11+ U106-	51.5	0.1
MXCHE	UK	R1b1a2a1a1*	R-L11+ U106-	51.5	0.1
96841	UK	R1b1a2a1a1*	R-L11+ U106-	51.5	0.1
13621	UK	R1b1a2a1a1*	R-L11+ U106-	51.5	0.1
19441	UK	R1b1a2a1a1*	R-L11+ U106-	51.5	0.1
8551	UK	R1b1a2a1a1*	R-L11+ U106-	51.5	0.1
24162	UK	R1b1a2a1a1*	R-L11+ U106-	51.5	0.1
N28007	UK	R1b1a2a1a1*	R-L11+ U106-	51.5	0.1
5789	UK	R1b1a2a1a1*	R-L11+ U106-	51.5	0.1
CSRF9	UK	R1b1a2a1a1*	R-L11+ U106-	51.5	0.1
112244	UK	R1b1a2a1a1*	R-L11+ U106-	51.5	0.1
63623	UK	R1b1a2a1a1*	R-L11+ U106-	51.5	0.1
9544	UK	R1b1a2a1a1*	R-L11+ U106-	51.5	0.1
62584	UK	R1b1a2a1a1*	R-L11+ U106-	51.5	0.1
ADCAU	UK	R1b1a2a1a1*	R-L11+ U106-	51.5	0.1
77763	UK	R1b1a2a1a1*	R-L11+ U106-	51.5	0.1
28079	UK	R1b1a2a1a1*	R-L11+ U106-	51.5	0.1
48674	UK	R1b1a2a1a1*	R-L11+ U106-	51.5	0.1

N16295	UK	R1b1a2a1a1*	R-L11+ U106-	51.5	0.1
16153	UK	R1b1a2a1a1*	R-L11+ U106-	51.5	0.1
N40675	UK	R1b1a2a1a1*	R-L11+ U106-	51.5	0.1
30333	UK	R1b1a2a1a1*	R-L11+ U106-	51.5	0.1
36483	UK	R1b1a2a1a1*	R-L11+ U106-	51.5	0.1
75621	UK	R1b1a2a1a1*	R-L11+ U106-	51.5	0.1
SXR68	UK	R1b1a2a1a1*	R-L11+ U106-	51.5	0.1
63671	UK	R1b1a2a1a1*	R-L11+ U106-	51.5	0.1
65048	UK	R1b1a2a1a1*	R-L11+ U106-	51.5	0.1
N40156	UK	R1b1a2a1a1*	R-L11+ U106-	51.5	0.1
3Y65B	UK	R1b1a2a1a1*	R-L11+ U106-	51.5	0.1
152974	Armenia	R1b1a2a1b	R-L23+ L584+	40.2	44.5
164200	Armenia	R1b1a2a1b	R-L23+ L584+	40.2	44.5
182984	Armenia	R1b1a2a1b	R-L23+ L584+	40.2	44.5
195789	Armenia	R1b1a2a1b	R-L23+ L584+	40.2	44.5
7PJJJ	Germany	R1b1a2a1b	R-L23+ L584+	52.5	13.4
183824	Iran	R1b1a2a1b	R-L23+ L584+	35.7	51.4
184027	Iraq	R1b1a2a1b	R-L23+ L584+	33.3	44.4
205749	Iraq	R1b1a2a1b	R-L23+ L584+	33.3	44.4
92187	Poland	R1b1a2a1b	R-L23+ L584+	52.2	21
70052	Syria	R1b1a2a1b	R-L23+ L584+	33.5	36.3
90492	Turkey	R1b1a2a1b	R-L23+ L584+	39.9	32.9
162122	Turkey	R1b1a2a1b	R-L23+ L584+	39.9	32.9
162122	Turkey	R1b1a2a1b	R-L23+ L584+	39.9	32.9
166322	Turkey	R1b1a2a1b	R-L23+ L584+	39.9	32.9
166323	Turkey	R1b1a2a1b	R-L23+ L584+	39.9	32.9
178932	Turkey	R1b1a2a1b	R-L23+ L584+	39.9	32.9
187550	Turkey	R1b1a2a1b	R-L23+ L584+	39.9	32.9
191405	Turkey	R1b1a2a1b	R-L23+ L584+	39.9	32.9
83298	Armenia	R2a*	R-M124+ L295- L294-	40.2	44.5
152979	Armenia	R2a*	R-M124+ L295- L294-	40.2	44.5
34064	Belarus	R2a*	R-M124+ L295- L294-	53.9	27.6
202994	Belarus	R2a*	R-M124+ L295- L294-	53.9	27.6
N50322	Belarus	R2a*	R-M124+ L295- L294-	53.9	27.6
N76442	Belarus	R2a*	R-M124+ L295- L294-	53.9	27.6
167022	Germany	R2a*	R-M124+ L295- L294-	52.5	13.4
204954	Germany	R2a*	R-M124+ L295- L294-	52.5	13.4
N15680	Hungary	R2a*	R-M124+ L295- L294-	47.4	19.2
47959	India	R2a*	R-M124+ L295- L294-	21	78
57968	India	R2a*	R-M124+ L295- L294-	21	78
79350	India	R2a*	R-M124+ L295- L294-	21	78
114482	India	R2a*	R-M124+ L295- L294-	21	78
173192	India	R2a*	R-M124+ L295- L294-	21	78
196275	India	R2a*	R-M124+ L295- L294-	21	78
N13831	India	R2a*	R-M124+ L295- L294-	21	78
N15633	India	R2a*	R-M124+ L295- L294-	21	78
N48849	India	R2a*	R-M124+ L295- L294-	21	78
N68553	India	R2a*	R-M124+ L295- L294-	21	78
N98791	India	R2a*	R-M124+ L295- L294-	21	78
166326	Iran	R2a*	R-M124+ L295- L294-	35.7	51.4
M5075	Iran	R2a*	R-M124+ L295- L294-	35.7	51.4
M6716	Iran	R2a*	R-M124+ L295- L294-	35.7	51.4
74611	Iran	R2a*	R-M124+ L295- L294-	35.7	51.4
N40042	Iran	R2a*	R-M124+ L295- L294-	35.7	51.4

166906	Kazakhstan	R2a*	R-M124+ L295- L294-	51.2	71.4
130681	Kuwait	R2a*	R-M124+ L295- L294-	29.4	47.9
M6364	Kuwait	R2a*	R-M124+ L295- L294-	29.4	47.9
M7063	Kuwait	R2a*	R-M124+ L295- L294-	29.4	47.9
7568	Latvia	R2a*	R-M124+ L295- L294-	56.9	24.1
N66759	Latvia	R2a*	R-M124+ L295- L294-	56.9	24.1
N36207	Levant	R2a*	R-M124+ L295- L294-	33.4	35.5
N47925	Levant	R2a*	R-M124+ L295- L294-	33.4	35.5
65829	Lithuania	R2a*	R-M124+ L295- L294-	54.7	25.3
114900	Lithuania	R2a*	R-M124+ L295- L294-	54.7	25.3
133728	Lithuania	R2a*	R-M124+ L295- L294-	54.7	25.3
139187	Lithuania	R2a*	R-M124+ L295- L294-	54.7	25.3
N89017	Pakistan	R2a*	R-M124+ L295- L294-	33.7	73.2
190496	Qatar	R2a*	R-M124+ L295- L294-	25.3	51.5
M7361	Qatar	R2a*	R-M124+ L295- L294-	25.3	51.5
76213	Russia	R2a*	R-M124+ L295- L294-	55.7	37.6
77686	Russia	R2a*	R-M124+ L295- L294-	55.7	37.6
152883	Russia	R2a*	R-M124+ L295- L294-	55.7	37.6
168753	Russia	R2a*	R-M124+ L295- L294-	55.7	37.6
N71654	Russia	R2a*	R-M124+ L295- L294-	55.7	37.6
226085	Saudi Arabia	R2a*	R-M124+ L295- L294-	24.6	46.7
M7355	Saudi Arabia	R2a*	R-M124+ L295- L294-	24.6	46.7
177636	Slovakia	R2a*	R-M124+ L295- L294-	48.2	17.1
204790	Sweden	R2a*	R-M124+ L295- L294-	59.4	18.1
N72507	Switzerland	R2a*	R-M124+ L295- L294-	46.9	7.4
69172	Tunisia	R2a*	R-M124+ L295- L294-	36.8	10.2
174252	Turkey	R2a*	R-M124+ L295- L294-	39.9	32.9
179132	Turkey	R2a*	R-M124+ L295- L294-	39.9	32.9
M6609	UAE	R2a*	R-M124+ L295- L294-	24.5	54.4
175576	UK	R2a*	R-M124+ L295- L294-	51.5	0.1
FXGT6	Ukraine	R2a*	R-M124+ L295- L294-	50.4	30.5
80336	Ukraine	R2a*	R-M124+ L295- L294-	50.4	30.5
173869	Ukraine	R2a*	R-M124+ L295- L294-	50.4	30.5
N13866	Ukraine	R2a*	R-M124+ L295- L294-	50.4	30.5
N32154	Ukraine	R2a*	R-M124+ L295- L294-	50.4	30.5
N34756	Ukraine	R2a*	R-M124+ L295- L294-	50.4	30.5
N47925	Ukraine	R2a*	R-M124+ L295- L294-	50.4	30.5
N65466	Ukraine	R2a*	R-M124+ L295- L294-	50.4	30.5
210978	Yemen	R2a*	R-M124+ L295- L294-	15.3	44.2
41130	Yemen	R2a*	R-M124+ L295- L294-	15.3	44.2
35521	India	R2a1a	R-M124+ L295+ L294-	21	78
70778	India	R2a1a	R-M124+ L295+ L294-	21	78
218123	India	R2a1a	R-M124+ L295+ L294-	21	78
E5825	India	R2a1a	R-M124+ L295+ L294-	21	78
N101460	India	R2a1a	R-M124+ L295+ L294-	21	78
N102097	India	R2a1a	R-M124+ L295+ L294-	21	78
N13638	India	R2a1a	R-M124+ L295+ L294-	21	78
N15633	India	R2a1a	R-M124+ L295+ L294-	21	78
N54928	India	R2a1a	R-M124+ L295+ L294-	21	78
N56907	India	R2a1a	R-M124+ L295+ L294-	21	78
N73992	India	R2a1a	R-M124+ L295+ L294-	21	78
N74077	India	R2a1a	R-M124+ L295+ L294-	21	78
N77183	India	R2a1a	R-M124+ L295+ L294-	21	78
N99821	India	R2a1a	R-M124+ L295+ L294-	21	78

N81759	India	R2a1a	R-M124+ L295+ L294-	21	78
213291	India	R2a1a	R-M124+ L295+ L294-	21	78
E11827	India	R2a1a	R-M124+ L295+ L294-	21	78
N100325	India	R2a1a	R-M124+ L295+ L294-	21	78
N13647	India	R2a1a	R-M124+ L295+ L294-	21	78
N20326	India	R2a1a	R-M124+ L295+ L294-	21	78
N20744	India	R2a1a	R-M124+ L295+ L294-	21	78
N37067	India	R2a1a	R-M124+ L295+ L294-	21	78
N38706	India	R2a1a	R-M124+ L295+ L294-	21	78
N55174	India	R2a1a	R-M124+ L295+ L294-	21	78
N55951	India	R2a1a	R-M124+ L295+ L294-	21	78
N58062	India	R2a1a	R-M124+ L295+ L294-	21	78
N74520	India	R2a1a	R-M124+ L295+ L294-	21	78
N91873	India	R2a1a	R-M124+ L295+ L294-	21	78
N96545	India	R2a1a	R-M124+ L295+ L294-	21	78
234700	Iraq	R2a1a	R-M124+ L295+ L294-	33.3	44.4
20461	Italy	R2a1a	R-M124+ L295+ L294-	41.9	12.5
69059	Pakistan	R2a1a	R-M124+ L295+ L294-	33.7	73.2
202218	Qatar	R2a1a	R-M124+ L295+ L294-	25.3	51.5
175653	Saudi Arabia	R2a1a	R-M124+ L295+ L294-	24.6	46.7
235768	Saudi Arabia	R2a1a	R-M124+ L295+ L294-	24.6	46.7
239090	Saudi Arabia	R2a1a	R-M124+ L295+ L294-	24.6	46.7
M5040	Saudi Arabia	R2a1a	R-M124+ L295+ L294-	24.6	46.7
168983	Syria	R2a1a	R-M124+ L295+ L294-	33.5	36.3
M5098	UAE	R2a1a	R-M124+ L295+ L294-	24.5	54.4
114870	UK	R2a1a	R-M124+ L295+ L294-	51.5	0.1

Table S11. **Fellow traveller haplogroups:** Transcaucasia to India. Marker incidence above the 2% threshold was plotted for each population sample.

**Community / Region**	***n***	**REFERENCE**
Abkhaz	*58*	1.9.2.
Afro-Iranian	*12*	1.9.3.
Agharia	*10*	1.9.6
Assyrian (Azarbaijan)	*39*	1.9.3.
Avars	*115*	1.9.2.
Azeri	*63*	1.9.3.
Bactria	*23*	1.9.5
Balochi	*25*	1.9.6
Begram	*19*	1.9.5
Brahui	*25*	1.9.6
Burusho	*20*	1.9.6
Chechens (Chechnya)	*118*	1.9.2.
Chechens (Dagestan)	*100*	1.9.2.
Chechens (Ingushetia)	*112*	1.9.2.
Circassians	*142*	1.9.2.
Dargins	*101*	1.9.2.
Fars Persian	*44*	1.9.3.
Gheshmi	*49*	1.9.3.
Gilan Gilak	*64*	1.9.3.
Golestan Turkmen	*68*	1.9.3.
Hazara Afghan	*60*	1.9.4
Hormozgan Bandari	*131*	1.9.3.
Ingush	*143*	1.9.2.
Iraq	*203*	1.9.1.
Isfahan Persian	*11*	1.9.3.
Iyengar	*30*	1.9.6
Iyer	*29*	1.9.6
Jalalabad-Laghman	*40*	1.9.5
Jordan	*146*	1.9.1.
Kaitak	*33*	1.9.2.
Kalash	*20*	1.9.6
Khatri	*29*	This work
Khorasan Persian	*59*	1.9.3.
Khuzestan Arab	*57*	1.9.3.
Konkanastha	*25*	1.9.6
Kubachi	*65*	1.9.2.
Kurdestan Kurd	*59*	1.9.3.
Lezghins	*80*	1.9.2.
Lorestan Lur	*50*	1.9.3.

Makrani	*20*	1.9.6
Maratha	*20*	1.9.6
Mazandarani	*72*	1.9.3.
Nuristan-Kunar	*15*	1.9.5
Oman	*121*	1.9.1.
Ossets-Digor	*127*	1.9.2.
Ossets-Iron	*230*	1.9.2.
Paktia	*13*	1.9.5
Pallan	*29*	1.9.6
Pashtun Afghan	*49*	1.9.4
Pathan	*21*	1.9.6
Qandahar-Herat	*19*	1.9.5
Qatar	*72*	1.9.1.
Rajput	*29*	1.9.6
Saraswat	*35*	This work
Saudi Arabia	*157*	1.9.1.
Shapsug	*100*	1.9.2.
Sindhi	*29*	1.9.6
Sistan Baluch	*24*	1.9.3.
Tajik Afghan	*56*	1.9.4
Tehran Armenian	*34*	1.9.3.
Tehran Assyrian	*9*	1.9.3.
Tehran Zoroastrian	*13*	1.9.3.
UAE	*164*	1.9.1.
UP Brahmins	*14*	1.9.6
Vanniyar	*25*	1.9.6
Yazd Persian	*46*	1.9.3.
Yazd Zoroastrian	*34*	1.9.3.
Yemen	*62*	1.9.1.

References:

1.9.1. Abu-Amero et al
1.9.2. Balanovsky O, Dibirova K, Dybo A, Mudrak O, Frolova S, Pocheshkhova E, Haber M, Platt D, Schurr T, Haak W, Kuznetsova M, Radzhabov M, Balaganskaya O, Romanov A, Zakharova T, Soria Hernanz DF, Zalloua P, Koshel S, Ruhlen M, Renfrew C, Wells RS, Tyler-Smith C, Balanovska E; Genographic Consortium. (2011) Parallel evolution of genes and languages in the Caucasus region. *Mol Biol Evol*. 28(10): 2905-2920.
1.9.3. Grugni V, Battaglia V, Hooshiar Kashani B, Parolo S, Al-Zahery N, Achilli A, Olivieri A, Gandini F, Houshmand M, Sanati MH, Torroni A, Semino O. (2012) Ancient migratory events in the Middle East: new clues from the Y-chromosome variation of modern Iranians. *PLoS One*. 7(7): e41252.
1.9.4. Haber M, Platt DE, Ashrafian Bonab M, Youhanna SC, Soria-Hernanz DF, Martínez-Cruz B, Douaihy B, Ghassibe-Sabbagh M, Rafatpanah H, Ghanbari M, Whale J, Balanovsky O, Wells RS, Comas D, Tyler-Smith C, Zalloua PA; Genographic Consortium. (2012) Afghanistan's ethnic groups share a Y-chromosomal heritage structured by historical events. *PLoS One*. 7(3): e34288.
1.9.5. Lacau H, Gayden T, Regueiro M, Chennakrishnaiah S, Bukhari A, Underhill PA, Garcia-Bertrand RL, Herrera RJ. (2012) Afghanistan from a Y-chromosome perspective. *Eur J Hum Genet.* 20(10): 1063-1070.
1.9.6. Sengupta S, Zhivotovsky LA, King R, Mehdi SQ, Edmonds CA, Chow CE, Lin AA, Mitra M, Sil SK, Ramesh A, Usha Rani MV, Thakur CM, Cavalli-Sforza LL, Majumder PP, Underhill PA. (2006) Polarity and temporality of high-

resolution y-chromosome distributions in India identify both indigenous and exogenous expansions and reveal minor genetic influence of Central Asian pastoralists. *Am J Hum Genet.* 78(2): 202-221.

File S12. **STR drift: Transcaucasia to India**. Mean-minus-mode values were plotted for 5 STRs selected from a small common subset of STRs analyzed across all regions. The other STRs were omitted either because there was no sigificant variance in repeat number or because the STR locus was bidirectionally hypervariable (see Methods). TC=Transcaucasia; AF=Afghanistan; IE=India (Eastern Region).

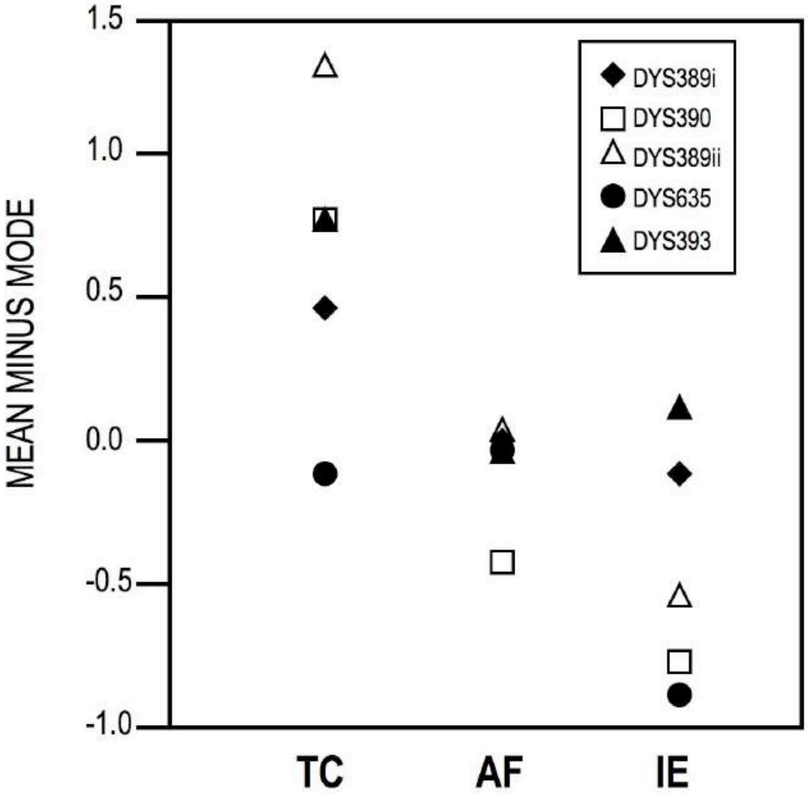

Table S13. **WP-BRW findspots**

Town	State	N	E	Layer	Arch Rev#	Date
Lothal	Gujarat	22.52	72.25	pre-Period II	*	2000-1700
Desalpur	Gujarat	23.2	69.44	I-B	*	2000-1700
Surkotada	Gujarat	23.12	70.83	I-B/C	*	2000-1700
Shikarpur	Gujarat	23.27	70.68	post-Harappan	1987-1988	2000-1700
Bagasra	Gujarat	21.29	70.57	post-Harappan	1999-2000	2000-1700
Rojdi	Gujarat	22.25	70.69	Period C?	*	2000-1700
Nagwada	Gujarat	23.33	71.68	post-Harappan	1985-1986	2000-1700
Rangpur	Gujarat	22.43	71.92	I-C-III; IIB	*	2000-1700
Ojiyana	Rajasthan	25.88	74.35	Period I	1999-2000	1900-1600
Ahar	Rajasthan	24.58	73.72	I-A	*	1900-1600
Balathal	Rajasthan	24.72	73.98	Chalcolithic	1993-1994	1900-1600
Gilund	Rajasthan	25.02	74.25		*	1900-1600
Kotra	MP	22.95	76.05	Period IA	1988-1989	1600-1200
Chichali	MP	22.13	75.40	Period I	1997-1998	1600-1200
Dangawada	MP	23.05	76.10	Period II	**	1600-1200
Maheshwar	MP	22.19	75.59		*	1600-1200
Eran	MP	24.10	78.16	Period IA	1987-1988	1600-1200
Navdatoli	MP	22.18	75.60	Phase I	*	1600-1200
Awra	MP	24.12	75.10		*	1600-1200
Prakash	Maharashtra	21.50	74.35	Period I	***	1600-1200
Imlidih Khurd	UP	26.55	83.21	Period II	1991-1992	1200-1000
Noh	Rajasthan	27.22	77.50		*	1200-1000
Kheradih	UP	25.76	84.15	Period I	1981-1982	1200-1000
Narhan	UP	26.76	83.37	Period I	1984-1985	1200-1000
Siswania	UP	26.75	82.77	Period I	1995-1996	1200-1000
Bhunadih	UP	25.76	84.15		1993-1994	1200-1000
Adam	Maharashtra	21.30	79.72	Period II	1989-1990	1200-1000
Chirand	Bihar	25.75	84.83	PdII	*	1200-1000
Dihar	W. Bengal	23.12	87.35	Period I	1993-1994	1200-1000
Hallur	Karnataka	14.33	75.62		*	1200-1000
Hemmige	Karnataka	12.47	76.08	Pd II	*	1200-1000
Maski	Karnataka	15.97	76.65		*	1200-1000
Sanganakallu	Karnataka	15.15	76.93	megalithic	*	1200-1000
Tekkalakotta	Karnataka	15.49	76.87	Phase II	*	1200-1000

Indian Archeological Review Issue Date

by radiocarbon dating (calibrated BCE)

*Ghosh A (1991) Encyclopedia of Indian Archaeology. Brill Academic Publishers, Leiden, Netherlands.

**Madhya Pradesh Archeological Directory

***Archeological Survey of India

Table S14. **Copper Hoard findspots.** Locations shown in bold type are the locations where WP-BRW finds have also been reported.

ID	Location
1	Aguibani
2	**Ahar**
3	Akhuldoba
4	Amroha
5	Andhari
6	Bagor
7	Bahadarabad
8	Baharia
9	Balpur
10	Bamanghati
11	Bandua
12	Bargaon
13	Bardangua
14	Bareilly
15	Barrajpur
16	Bartola
17	Bhagada
18	Bhaktabundh
19	Bhiwani
20	Bisauli
21	Bithur
22	Brahmagiri
23	**Chandoli**
24	Chansar
25	Chatla
26	**Chirand**
27	Dadari
28	Daimabad
29	Dargama
30	Debakia
31	Deoti
32	Dhaka
33	Dimiria
34	Ekalsimgha
35	Elana
36	Eran
37	Etawah
38	Fathgarh
39	Gandhauli
40	Ganeshwar
41	Ghangharia
42	Hallur
43	Hami
44	Hansi
45	Hardi

46	Harra Chowra Darh
47	Inamgaon
48	Indilapur
49	Jabalpur
50	**Jajmau**
51	Jamboni
52	Jokha
53	Jorwe
54	Kallur
55	Kamalpur
56	Kamdara
57	Kankasa
58	Karharbari
59	Katmandu Valley
60	Kayatha
61	Kesli
62	Kesna
63	Khera Manpur
64	Kindhaulia
65	Kiratpur
66	**Kausambi**
67	Kota
68	Kulgara
69	Kulhade-ka-Johade
70	Kurada
71	Kushaya
72	Lal Qila
73	Langnaj
74	**Lothal**
75	Ludurapada
76	Madnapur
77	Mahisadal
78	Mahuadanr
79	Mainpuri
80	Mallah
81	Maski
82	Mathura
83	Mitathal
84	Moongalaar
86	Nagar
87	Nakrahiya
88	Nandlalpura
89	Nankom
90	Narnaod
91	Narsimhapur
92	Nasirpur
93	Navdatoli
94	Nevasa
95	Niorai

96 Pandu Rajar Dhibi
97 Pariar
98 Parihati
99 Pauli
100 Perua
101 Piklihal
102 Pind
103 Pondi
104 Prakash
105 Rajpur Parsu
106 Ramapuram
107 Ramjipura
108 Rangpur
109 Resgavaon
110 Rewari
111 Sabania
112 Saguni
113 Saipai Lichchwal
114 Sanchan Kot
115 Sandhay
116 **Sanghol**
117 Sankarjang
118 Sarthauli
119 Sastevadi
120 Shahabad
121 Shavinipatti
122 Sheorajpur
123 Somnath
124 **Sonepur**
125 Tamajuri
126 Taradih
127 Tekkalakota
128 Terdal
129 Viratgarh

REFERENCES:

1. Yule P (1985) *Metalwork of the Bronze Age in India*. Oscar Beck, Munich.

2. Yule P (1992) The Copper Hoards Of The Indian Subcontinent. *Jahrbuch des Römisch-Germanischen Zentralmuseums Mainz* 36: 193–275.

Table S15. **Channel-spouted bowl findspots.**

Location	State	N	E	Period	References
Nagarjunakonda	AP	16.57	79.31		Ghosh
Rangpur	Gujarat	22.43	71.92		Ghosh
Lothal	Gujarat	22.52	72.25		Ghosh
Surkotada	Gujarat	23.63	70.84		Ghosh
Piklihal	Karnataka	15.96	76.45	IA-IB	Sankalia, Ghosh
Hemmige	Karnataka	12.36	76.08	II	Ghosh
T. Narsipur	Karnataka	12.21	76.91	I-II	Sankalia, Ghosh
Sanganakallu	Karnataka	15.18	76.97		Ghosh
Brahmagiri	Karnataka	14.00	76.50	IB	Ghosh
Chandoli	Maharashtra	19.17	73.97	Layer 2	Ghosh
Daimabad	Maharashtra	19.52	74.70		Sankalia
Nevasa	Maharashtra	19.57	74.90		Sankalia
Navdatoli (Maheshwar)	MP	22.18	75.60	III-IV	Sankalia, Ghosh
Eran	MP	24.08	78.17	I	Sankalia
Panihar (Gupteshwar)	MP	26.09	78.01	II	Ghosh
Ahar	Rajasthan	24.58	73.72		Ghosh
Gilund	Rajasthan	25.02	74.25		Sankalia
Khurdi-Parbatsar	Rajasthan	26.89	74.77		Sankalia, Ghosh

REFERENCES:

1.Ghosh A (1991) Encyclopedia of Indian Archaeology. Brill Academic Publishers, Leiden, Netherlands.

2. Sankalia HD (1963) New Light on the Indo-Iranian or Western Asiatic Relations between 1700 B.C.-1200 B.C. *Artibus Asiae* 26(3-4): 312-332.

Table S16. **Votive tanks findspots**

Location	State	N	E
Ahar (Aghata)	Rajasthan	27.70	75.63
Ahichchatra	UP	28.37	79.12
Arikamedu	TN	11.92	79.83
Ayodhya	UP	26.80	82.20
Bairat	Rajasthan	27.45	76.20
Bateswar	??	26.93	78.70
Besnagar (Vidisha)	MP	23.53	77.82
Bhita (Allahabad)	UP	25.45	81.85
Bhokardan	Maharashtra	20.27	75.77
Chandoli (Sangli)	Maharashtra	16.87	74.57
Chirand	Bihar	25.75	84.80
Devnimori	Gujarat	23.67	73.35
Hastinapur	UP	29.15	78.05
Jajmau (Yayatimau)	UP	26.47	80.35
Kaundinyapura	Maharashtra	22.92	78.10
Kausambi	UP	25.33	81.38
Kolhapur (Brahmapuri)	Maharashtra	16.70	74.23
Maheshwar (Mahishmati)	MP	22.18	75.60
Masaon	UP	25.17	82.42
Mathura	UP	27.52	77.23
Nagari (Madhyamika)	Rajasthan	24.90	74.63
Nagarjunakonda	TN	16.52	79.23
Nala Sopara	Maharashtra	19.42	72.86
Navdatoli	MP	22.18	75.60
Nevasa	Maharashtra	19.57	74.90
Noh (Bharatpur)	Rajasthan	27.22	77.48
Paunar (Pravarapura)	Maharashtra	20.79	78.68
Purana Qila	Haryana	28.63	77.20
Rairh (Tonk District)	Rajasthan	26.33	76.17
Rajghat (Gorakhpur)	UP	26.76	83.37
Rajgir (Rajagriha)	Bihar	25.02	85.50
Rangamahal	Rajasthan	29.35	73.95
Sambhar (Sakambhari)	Rajasthan	26.92	75.20
Sanghol	Punjab	30.78	76.38
Shamlaji	Gujarat	23.69	73.39
Sonepur	Bihar	25.70	85.18
Sonkh	UP	27.50	77.47
Taxila (Sirkap)	Punjab	33.75	72.79
Ter (Tagara)	Maharashtra	18.32	76.21
Ujjain	MP	23.19	75.78

* 18/40 (45%) of findspots are associated with Jaina Tirthas or religious centers; by comparison, only 6/81 (7.5%) of other contemporary archeological sites on the Indian sub-continent are associated with Jaina Tirthas or centers. The relative co-incidence of Buddhist centers is 6/40 (15%) and 12/81 (14.9%) respectively.

Table S17. **Toponym table.** God/epic hero (word-stem) toponyms were scored for each of the 20 largest states, corrected for relative incidence.

	**RELATIVE INCIDENCE OF GOD TOPONYMS** (BY MAJOR STATE, RELATIVE TO NATIONAL AVERAGE)						
	**MITRA VARUNA**	**OTHER VEDIC**	**GANESH**	**SHIVA**	**LAXMI NARAYAN**	**RAMAYANA**	**(MAHA) BALI**
	(n=73)	*(n=49)*	*(n=56)*	*(n=414)*	*(n=263)*	*(n=216)*	*(n=217)*
Andhra Pradesh				**1.32**			
Assam							**14.64**
Bihar					**1.89**	**2.04**	
Chattisgarh					**1.12**	**1.69**	
Gujarat	**4.49**	**1.77**	**3.89**	**1.52**	**1.40**		
Haryana					**1.53**		**1.86**
Himachal Pradesh							**2.75**
Jammu & Kashmir							
Jharkhand					**2.01**	**1.97**	**2.98**
Karnataka				**1.76**			
Kerala							
Madhya Pradesh		**1.58**	**1.54**			**1.70**	
Maharashtra			**2.22**	**2.22**			
Orissa	**1.15**				**1.69**	**1.36**	**6.16**
Punjab							**1.84**
Rajasthan	**2.07**		**3.45**			**1.93**	
Tamilnadu	**1.20**						
Uttar Pradesh	**1.30**		**1.68**			**1.39**	
Uttarakhand							
West Bengal					**1.40**	**2.06**	**1.7**

Table S18. **Harappan seal styles**. Tabulation of seals with animal motifs (available online October 2012).

		RELATIVE INCIDENCE OF ANIMAL MOTIFS					
FINDSPOT *(REGION)*	***n***	***UNICORN***	***CAPRID***	***BOVID***	***OTHER****	***HYBRIDS***	***UNCLASSIF***
SOTHI SISWAL	**26**	**38.5**	**7.7**	**7.7**	**34.6**	**7.7**	**3.8**
HARAPPA	**33**	**75.8**	**0.0**	**6.1**	**12.1**	**0.0**	**6.1**
MOHENJODARO	**113**	**70.8**	**2.7**	**4.4**	**17.7**	**2.7**	**1.8**
SOUTHERN SINDH	**20**	**42.1**	**31.6**	**10.5**	**10.5**	**5.3**	**0.0**
SORATH	**13**	**46.2**	**7.7**	**15.4**	**0.0**	**30.8**	**0.0**
PCT. SUN-CIRCLE**		**3.9**	**100.0**	**0.0**	**0.0**	**90.0**	**n.d.**

*elephant, tiger, rhino, sambhar
**co-occurrence of dot-circle sun symbol

Table S19. **Tribal coins: Northwest region.** Percent occurrence of symbols on tribal coins of NW region of subcontinent during the interregnum between Mauryan and Gupta empires (200 BCE-300 CE). WEST= Gandhara, W. Punjab, 4 tribes, 8 coin series; EAST= Himachal Pradesh region, 4 tribes, 10 coin series; SOUTH= Haryana, Rajasthan, Malwa, 8 tribes, 27 coin series; south region inscriptions are in Brahmi only, but in other regions inscriptions are in both Brahmi and Kharoshti; **auspicious symbols (ashtamangalas) in the Svetambara Jain tradition but not in the Buddhist or Hindu traditions; #associated with kings whose names are in the Shaiva tradition — always on the Brahmi side of coins.

SYMBOL	**WEST**	**EAST**	**SOUTH**
---Ujjain symbol	0.0	0.0	**21.7**
---Tree-in-Railing	0.0	10.0	**82.6**
---Indradhvaja	0.0	**40.0**	0.0
---Swastika**	25.0	**70.0**	0.0
---Srivatsa**	25.0	**40.0**	0.0
---Trisula #	**62.5**	30.0	0.0

Reference: Handa D. “Tribal Coins of Ancient India” Aryan Books, New Delhi, 2007.

Table S20. **Coins of the Indo-Persian borderlands.** Early historical period.

King (Indo-Greek)	Date	Location	OBVERSE SYMBOLS	REVERSE SYMBOLS
Pantaleon	190-180 BCE	Arachosii	Female deity, flower	Lion
Antimachos I	185-170 BCE	Arachosii	Bust of king	Poseidon w/ trident
Apollodotos I	180-160 BCE	Gandareii	Elephant	Humped Bull
Apollodotos I	180-160 BCE	Gandareii	Elephant, sun, kur-star/river	Humped bull, taurine
Apollodotos I	180-160 BCE	Gandareii	Elephant	Humped Bull
Apollodotos I	180-160 BCE	Gandareii	Apollo, bow, arrow	tripod
Demetrios II	175-140 BCE	Arachosii	King	Athena, spear, shield
Antimachos II Nikephoros	160-155 BCE	Gandareii	Nike, palm, fillet	King on horse
Eucratides I	170-145 BCE	Gandareii	King (horn bull helmet)	Dioscuri (mounted), palm, spear
Eucratides I	170-145 BCE	Gandareii	King (crested helmet)	Dioscuri, palms & pilei
Eucratides I	170-145 BCE	Gandareii	King (crested helmet)	Dioscuri (mounted), palm, spear
Eucratides I	170-145 BCE	Gandareii	King (crested helmet)	Dioscuri (mounted), palm & spear
Eucratides II	170-145 BCE	Gandareii	King	Apollo, bow, arrow
Heliocles I	145-130 BCE	Bactria	King	Zeus, thunderbolt & scepter
Heliocles I (posthumous)	145-130 BCE	Bactria	King	Horse
Menander I Soter	155-130 BCE	Gandareii-E	Athena (helmet)	Owl
Menander I Soter	155-130 BCE	Gandareii-E	King, spear	Athena, thunderbolt & shield
Menander I Soter	155-130 BCE	Gandareii-E	King, spear	Athena, thunderbolt & shield
Menander I Soter	155-130 BCE	Gandareii-E	King, spear	Athena Alkidemos, shield
Menander I Soter	155-130 BCE	Gandareii-E	King, spear	Athena Alkidemos, shield
Menander I Soter	155-130 BCE	Gandareii-E	King, spear	Athena, thunderbolt & shield
Menander I Soter	155-130 BCE	Gandareii-E	King (cr. Helmet), spear	Athena, thunderbolt & shield
Menander I Soter	155-130 BCE	Gandareii-E	King (cr. Helmet), spear	Athena, thunderbolt & shield
Menander I Soter	155-130 BCE	Gandareii-E	Athena (helmet)	Winged Nike, wreath, palm
Zoilos I	130-20 BCE	Arachosii	King	Heracles, crowned, club, lion skin
Zoilos I	130-20 BCE	Arachosii	King	Heracles, crowned, club, lion skin
Zoilos I	130-20 BCE	Arachosii	Heracles w/ lions skin	Club, bow, wreath
Strato I	120-110 BCE	W. Punjab	King	Athena, thunderbolt & shield
Lysias	120-110 BCE	Arachosii	King (helmet) spear, elephant	Heracles, crowned, club, lion skin
Lysias	120-110 BCE	Arachosii	King (helmet) spear, elephant	Heracles, crowned, club, lion skin
Antialcidas	110-100 BCE	Arachosii	King	Zeus, scepter & palm, tiny Nike
Antialcidas	110-100 BCE	Arachosii	King (helmet)	Zeus, scepter & palm, tiny Nike
Antialcidas	110-100 BCE	Arachosii	King (helmet)	Zeus, scepter & palm, tiny Nike
Antialcidas	110-100 BCE	Arachosii	Zeus, thunderbolt	Dioscuri, palm, pilei
Heliocles II	110-100	Punjab - W	King	Zeus, scepter & thunderbolt
Philoxenos	100-95 BCE	Gandareii	King	King (helmet) on horse
Philoxenos	100-95 BCE	Gandareii	King (helmet)	King (helmet) on horse
Amyntas	95-90 BCE	Gandareii	King (helmet) spear	Zeus, scepter & palm, tiny Athena
Archebios	90-70 BCE	Punjab -W	King	Zeus, scepter & thunderbolt
Archebios	90-70 BCE	Punjab -W	King (helmet)	Zeus, scepter & thunderbolt
Hermaios & Calliope	90-70 BCE	Begram	Kings	King (helmet) on horse
Hermaios	90-70 BCE	Begram	King	Zeus, scepter
Hermaios	90-70 BCE	Begram	King (helmet)	Zeus, scepter
Hermaios	90-70 BCE	Begram	King (helmet)	Zeus, scepter
Hermaios (posthumous)	90-70 BCE	Begram	King	Zeus, scepter
Hermaios (posthumous)	90-70 BCE	Begram	King	Zeus, scepter
Hermaios (posthumous)	90-70 BCE	Begram	King	Zeus, scepter

Hermaios (posthumous)	90-70 BCE	Begram	King	Zeus, scepter
Hermaios (posthumous)	90-70 BCE	Begram	King	Zeus, scepter
Hermaios (posthumous)	90-70 BCE	Begram	King	Zeus, scepter
Hermaios (posthumous)	90-70 BCE	Begram	King	Zeus, scepter
Hermaios (posthumous)	90-70 BCE	Begram	King	Zeus, scepter
Apollodotus II	75-70 BCE	Punjab	King	Athena, shield, thunderbolt
Apollodotus II	75-70 BCE	Punjab	King	Athena, shield, thunderbolt
Apollodotus II	75-70 BCE	Punjab	Apollo, quiver, arrow, GK	Tripod
Zoilos II	55-35 BCE	Punjab	King	Athena, shield, thunderbolt
Strato II	25-10 BCE	Punjab	King	Athena, shield, thunderbolt

**King (Indo-Scythian)**	**Date**	**Location**	**Series OBV**	**Series REV**
Maues	85-60 BCE	Gandhara	Elephant, bull	Caduceus
Maues	85-60 BCE	Gandhara	Zeus, thunderbolt, trident	Goddess, vines
Maues	85-60 BCE	Gandhara	Elephant	King, sword
Vonones / Spalahores	75-65 BCE	unknown	King on horse, spear	Zeus, scepter, thunderbolt
Vonones / Spalagadames	75-50 BCE	unknown	King on horse, spear	Zeus, scepter, thunderbolt
Spalirises / Spalagadames	60-50 BCE	unknown	King on horse	Heracles, club
Spalirises / Spalagadames	60-50 BCE	unknown	King, battle axe, bow	Zeus radiate, gesture
Azes I	60-20 BCE	Gandhara	King on horse, spear	Zeus, scepter, thunderbolt
Azes I	60-20 BCE	Gandhara	King on horse, spear	Zeus, scepter, thunderbolt
Azes I	60-20 BCE	Gandhara	King on horse, spear	Zeus, scepter, thunderbolt
Azes I	60-20 BCE	Gandhara	King on horse, spear	Goddess, palm, lamp
Azes I	60-20 BCE	Gandhara	King on horse, spear	Athena, shield, thunderbolt
Azes I	60-20 BCE	Gandhara	King on horse, spear	Humped bull
Azes I	60-20 BCE	Gandhara	Poseidon, trident	Goddess, vines
Azilises	70-60 BCE	Gandhara	King on horse, spear	Goddess, palm, lamp
Azilises	70-60 BCE	Gandhara	King on horse, spear	Goddess, palm, lamp
Azilises	70-60 BCE	Gandhara	King on horse, spear	Athena, shield, thunderbolt
Azes II	60-20 BCE	Gandhara	King on horse, whip	Poseidon, trident
Azes II	60-20 BCE	Gandhara	King on horse, whip	Zeus, w. Nike, scepter
Azes II	60-20 BCE	Gandhara	King on horse, whip	Zeus, w. Nike, scepter
Azes II	60-20 BCE	Gandhara	King on horse, whip	Zeus, w. Nike, scepter
Azes II	60-20 BCE	Gandhara	King on horse, whip	Zeus, w. Nike, scepter
Azes II	60-20 BCE	Gandhara	King on horse, whip	Athena, spear, shield
Azes II	60-20 BCE	Gandhara	King on horse, whip	Athena, spear, shield
Azes II	60-20 BCE	Gandhara	King on horse, whip	Athena, spear, shield
Azes II	60-20 BCE	Gandhara	King on horse, whip	Athena, spear, shield
Azes II	60-20 BCE	Gandhara	King on horse, whip	Athena, spear, shield
Azes II	60-20 BCE	Gandhara	goddess, cornucopia	Hermes, caduceus
Azes II	60-20 BCE	Gandhara	Humped bull	Lion
Azes II	60-20 BCE	Gandhara	Humped bull	Lion
Azes II (Malakand)	60-20 BCE	Gandhara	King on horse, whip	Zeus, w. Nike, scepter
Azes II (Malakand)	60-20 BCE	Gandhara	King on horse, whip	Athena, spear, shield
Azes II / Aspavarma (Malakand)	15-45 CE	Bajaur	King on horse, whip	Athena, spear, shield

**King (Indo-Parthian)**	**Date**	**Location**	**Series OBV**	**Series REV**
Gondophares	20 BCE -early CE	Arachosia-Gandh	King on horse, Nike, wreath	Shiva, trident, palm
Gondophares	20 BCE -early CE	Arachosia-Gandh	King on horse, gesture	Shiva, trident, palm

Gondophares	20 BCE -early CE	Arachosia-Gandh	King on horse, gesture	Athena, spear, shield
Gondophares	20 BCE -early CE	Arachosia-Gandh	King on horse, gesture	Zeus, gesture, scepter
Gondophares	20 BCE -early CE	Arachosia-Gandh	King on horse, gesture	Athena, spear, shield
Abdagases	50-65 CE	Arachosia	King on horse, gesture	Zeus, gesture, scepter
Abdagases	50-65 CE	Arachosia	King on horse, gesture	Zeus, gesture, scepter
Abdagases	50-65 CE	Arachosia	King on horse, gesture	Zeus, w. Nike, scepter
Abdagases	50-65 CE	Arachosia	King on horse, gesture	Zeus, gesture, scepter

Source: Smithsonian Collection.

Tally of above series:

	Series	*Heracles/Balarama-Lion*	*Trident (Shiva)**	*Humped Bull*
**Arachosii**	**22**	**7**	**2**	**0**
**Gandareii**	**52**	**0**	**0**	**6**
**Punjab**	**9**	**1**	**0**	**0**
**Begram**	**12**	**0**	**0**	**0**
**Bactria**	**2**	**0**	**0**	**0**
**Other**	**6**	**1**	**0**	**0**
**Total**	**103**	**9**	**2**	**6**

** not Poseidon*

Table S21. **Word-stem incidence in ancient texts.** The oldest books of the Rigveda (books 2-7), Atharvaveda, Ramayana (books 2-6) and four Puranas (Vayu, Vishnu, Matsya, Linga) were used for scoring the incidence of god/hero word stems.

		WORD STEM INCIDENCE PER 1000 WORDS			
		**Rigveda (Books 2-7)**	**Atharvaveda**	**Ramayana (Books 2-6)**	**Puranas (4)**
		Est. 1200 BCE	*Est. 900 BCE*	*Est. 400 BCE*	*Est. 600 CE*
Group ^	n	121668	160488	307509	211488
MITRAVARUNA	2124	3.90 ± 0.63*	1.67 ± 0.38	0.03 ± 0.06	0.32 ± 0.27
RAMAYANA	4234	0.01 ± 0.01	0.01 ± 0.02	4.11 ± 0.03**	0.07 ± 0.02
PURANIC	1517	0.22 ± 0.31	0.23 ± 0.32	0.06 ± 0.09	4.91 ± 1.49

^ Mitravaruna group: Mitra, Varuna, Surya, Sarasvati; Ramayana group: Rama, Sita, Raghu, Lakshman; Puranic group: Shiva, Vishnu;

* p<0.05 ** p<0.01 (vs other groups);

Table S22. **Harappan symbols on seals**

	**RELATIVE GEOGRAPHICAL INCIDENCE**					
Icon	***FISH***	***SUN***	***FIRE***	***TRIDENT***	***PIPAL***	***CROWN***
Wells symbol	*112-152*	*342, 354, 389*	*390-411*	*262*	*253-261*	*263, 265*
n	**812**	**133**	**120**	**116**	**42**	**122**
Significance	Fertility	Sun	Fire	Ishkur	Goddess	Horned god
Sothi-Siswal	**0.77**	**1.02**	**0.91**	**0.47**	**0.65**	**0.00**
Harappa	**0.93**	**0.70**	**0.80**	**0.66**	**1.02**	**1.84**
Mohenjodaro	**1.05**	**1.10**	**0.99**	**1.09**	**0.95**	**0.73**
S. Sindh	**0.97**	**1.63**	**1.13**	**2.58**	**2.59**	**0.00**
Sorath	**1.00**	**1.19**	**2.31**	**1.19**	**0.47**	**0.49**

Total number of signs tabulated =7,121 (Wells, 1998);

Table S23. Tripitaka census table

Place	*Modern Names*	*N*	*E*	*Page**
Agni	Karasahr	42.01	86.55	21
Ahicchattra	Ramnagar, Bareilly	28.37	79.12	135
Anandapura	Vadnagar	23.79	72.64	343
Andarab	Baghlan, Bactria	35.60	68.68	359
Andhra	Pedavegi	16.77	81.10	313
Atali	Atali	22.01	72.86	341
Audumbatira	Kaccheswara	23.70	68.53	348
Avanda	Bahmana (Mansura)	25.88	68.77	351
Ayamukha	Ghazipur	25.58	83.57	155
Ayodhya	Faizabad	26.80	82.20	152
Badakshan	Badakshan (mid)	38.00	71.00	362
Baghlan	Baghlan	36.13	68.70	34
Baktra	Mazari Sharif	36.77	66.87	34
Baluka	Aksu	41.17	80.25	25
Bamiana	Bamyan	34.82	67.82	37
Baranasi	Varanasi	25.28	82.96	195
Bharukacchapa	Bharuch	21.70	72.97	337
Bukhara	Bukhara	39.77	64.43	30
Campa	Bhagalpur	25.25	87.00	296
Cinabhukti	Chiniot	31.72	72.97	118
Cola	Thanjavur	10.77	79.13	318
Darada	Gilgit	35.92	74.28	92
Dhanakataka	Dharanikota	16.58	80.31	315
Dharmasthiti	Fayzabad? Khamdadh	37.12	70.58	363
Dravida	Kanchipuram	12.82	79.71	319
Feihan	Ferghana	40.38	71.79	28
Gandhara	Peshawar	34.02	71.58	70
Govisana	Kashipur, UK	29.22	78.95	134
Gurjara	Bhinmal	25.00	72.25	344
Himatala	Darayim?	36.92	70.38	362
Horismika	Khwarezm	41.38	60.37	31
Huoh	Kunduz	36.73	68.87	360
Jaguda	Hexina (Ghazni)	33.55	68.42	357
Jalamdhara	Jalandhar	31.21	75.57	120

Kajangala	Kajangala	25.03	87.84	297
Kalinga	Bhubaneshwar	20.27	85.84	306
Kamarupa	Guwahati	26.19	91.73	299
Kanyakubja	Kannauj	27.07	79.92	139
Kapilavastu	Lumbini	27.47	83.28	173
Kapisi	Kabul	34.53	69.17	39
Kapitha	Sankasya	27.52	82.05	136
Karnasuvarna	Karnasubarna	24.02	88.18	303
Kasanna	Shahrisabz	39.05	66.83	31
Kasmira	Srinagar	34.08	74.78	100
Kausambi	Kausambi	25.34	81.39	159
Khulm	Kholm	36.68	67.68	34
Khutalan	Khatlon	37.83	69.00	33
Kongoda	Ganjam	19.38	85.07	306
Konkanapura	Banavasi	14.53	75.02	334
Kosala (south)	Raipur	21.14	81.38	307
Kuci	Kucha	41.65	84.90	22
Kuluta	Naggar, Kullu	32.12	77.17	120
Kusinagara	Kushinagar	26.74	83.89	185
Lampa	Bagram	34.97	69.28	47
Langala	Sthuliswara	21.88	71.73	349
Magadha	Pataliputra	25.61	85.14	221
Maharashtra	Paithan on Godavari	19.48	75.38	335
Maheshvarapura	Mahishmati	22.11	75.35	345
Malakuta	Kanyakumari	8.08	77.54	321
Malava (S. Lata)	Kheda (Kaira)	22.75	72.68	338
Mathura	Mathura	27.49	77.67	122
Matipura	Mandawar	29.50	78.13	128
Mimohe	Panjakent	39.50	67.62	30
Mulasthanapura	Multan	30.19	71.47	347
Mungan	Takhar Province	36.70	69.80	361
Nagarahara	Jalalabad	34.43	70.44	65
Nujkend	Shymkent	42.32	69.59	28
Parnotsa	Poonch	33.77	74.10	110
Parvata	Faisalabad	31.42	73.07	347
Patasila	Hyderabad	25.37	68.37	350
Prayaga	Allahabad	25.45	81.85	156

Pundravardhana	Mahasthangarh	24.96	89.33	298
Puskaravati	Charsadda	34.80	71.43	77
Sahaniyan	Surkhan	37.74	67.52	32
Samarkand	Samarkand	39.65	66.96	29
Samatata	Narsingdi	23.92	90.73	301
Satadru	Ludhiana	30.91	75.85	121
Shash	Tashkent	41.27	69.22	28
Shuman	Dushanbe	38.54	68.78	33
Sravasti	Shravasti	27.52	82.05	165
Srughna	Saharanpur	29.96	77.55	126
Sthaneswara	Thanesar	29.97	76.84	124
Surattha	Surat, Kathiawad	21.17	72.83	343
Sutrushana	Utrushana, Khujand	40.28	69.63	29
Takka	Lahore	31.54	74.34	111
Taksasila	Taxila	33.74	72.78	93
Talaqan	Taloqan	36.72	69.52	36
Tamralipti	Tamluk	22.30	87.92	302
Taras	Taraz (Kazakh)	42.90	71.37	28
Tirmidh	Termez	37.22	67.28	32
Udakhand	Hund	33.89	72.24	80
Udyana	Mingora, Swat	34.78	72.37	83
Ujjaini	Ujjain	23.18	75.78	344
Vaishali	Vaishali	25.99	85.13	209
Valabhi	Vallabhi	21.89	71.88	342
Varnu	Bannu	32.99	70.62	352
Varsa	Varsa	34.00	71.70	78
Vrjisthana	Hubina (Vardak)	34.40	68.40	358
Wakhsh	Vakhsh	37.63	68.83	33

*Tripitaka: Rongxi L. (tr.) (1996) *The Great Tang Dynasty Record of the Western Regions*. Numata Center Buddhist Translation, Berkeley, CA.

Table S24. **Listing of vibhedas of Bharata-varsha** (Skanda Purana, 1st millennium CE). –ka vibhedas are shown in bold type.

SKANDA PURANA	***MODERN NAMES***
Amala	Andhra
Asvamukha	Rae Barelli, UP
Atisindhu	Upper Sindh
**Balaka**	Bahlika
Bhadra	Bhadra, Rajasthan
Cita	*unknown*
Dahala	Chedi, Baghelkhand
Devabhadra	*unknown*
Drada	Darada, Gilgit
Ekabahudesa	*unknown*
Ekapada	near Nepal east
**Gajanaka**	Ghazni
Gauda desa	Bengal
Gujaratta	Gujarat
Hariala	Haryana
Jahahuti	Khajuraho (Jejabhukti)
Jalandhara	Jullundhar
Jangala	Kuru-janagla near Saraswati
Kaccha mandala	Kutch
Kalanjara	Kashmir border
Kamarupa	Assam
Kamboja	Kamboja (Arachosia)
Kamtipura	Mirzapur, UP
Kanyakubja	Kannoj
Karnata	Karnataka
Kasmira	Kashmir
Kaumkana	Konkan-N
**Khurasanaka**	Khorasan
Kosala	Kosala
Laghu-Kaumkana	Konkan-S
Lata	Lata-Gujarat
Lohapura	Lahore
Macipura	Kamcipura
Magadha desa	Magadha
Malava	Malwa
Mevada	Mewar, Rajasthan
Mulasthana	Multan
Naremdu	Varendra
Nepala	Nepal
Nilapura	Kishenganga near Kashmir
Nirvarta mandala	NW Bengal
Oddiyana desa	Swat
Pambipura	*unknown*

Pamdu	Pandya
Pulastya visaya	*unknown*
Rataraja	Ratta-rajya (Rashtrakuta)
**Romaka**	Rumma-desa (Rajasthan)
Samjayu	Sanjan
Saurashtra	Saurashtra
Sayambhara	Sakambhara, Rajasthan
Sibi desa	Sibi/Swat
Sindhu	Sindh
Stri-rajya	north of Kasmir
**Surparaka**	Sopara (Thana)
Tilanga des	Telengana
Tomara	Rajasthan
Vaguri	Baged, Rajasthan
**Vambhanavaka**	Bahmanabad, Sind
Vidarbha	Vidarbha
Virata	Bairat, Jaipur
Yamakoti	*Mythical city (east of Lanka)*
Yavana desa	Sindh

Ciancaglini C. (2012) Outcomes of the Indo-Iranian Suffix *-ka- in Old Persian and Avestan. In: *Dariosh Studies II. Persepolis and its settlements: territorial system and ideology in the achaemenid state*. Basello GP and Rossi AV (eds) p.91-100. Naples.

Table S25. **Gotra: Percent incidence by group in major Brahmin communities.**

Community	Mother Tongue	n	G/B*	Vasishta	Bhargava	Kashyap	Angiras	Vishwamitra	Kutsa	Atri
All Brahmins	all	32182	G	*8.37*	*9.59*	*27.66*	*46.15*	*5.57*	*0.58*	*2.07*
All Brahmins	all	18007	B	*8.55*	*8.90*	*25.08*	*49.06*	*5.91*	*0.59*	*1.91*
GSB	Marathi	249	G	*21.69*	*28.11*	*9.24*	*14.46*	*22.89*	*0.40*	*3.21*
GSB	Marathi	179	B	*23.46*	*18.99*	*10.61*	*14.53*	*29.05*	*0.56*	*2.79*
GSB	Konkani	504	G	*16.07*	*15.28*	*18.45*	*16.07*	*5.95*	*21.83*	*6.35*
GSB	Konkani	285	B	*17.89*	*15.09*	*17.89*	*12.63*	*9.47*	*20.00*	*7.02*
Saraswat	Punjabi	200	G	*15.00*	*19.50*	*10.50*	*52.00*	*1.50*	*0.00*	*1.50*
Saraswat	Punjabi	243	B	*20.58*	*19.75*	*9.47*	*46.91*	*2.06*	*0.00*	*1.23*
Audichya	Gujarati / Kutchi	321	G	*9.35*	*11.21*	*17.76*	*50.47*	*10.28*	*3.43*	*0.93*
Audichya	Gujarati / Kutchi	186	B	*11.29*	*13.98*	*13.98*	*48.92*	*11.29*	*5.38*	*0.54*
Nagar	Gujarati / Kutchi	56	G	*19.64*	*1.79*	*55.36*	*19.64*	*1.79*	*0.00*	*1.79*
Nagar	Gujarati / Kutchi	39	B	*23.08*	*2.56*	*41.03*	*28.21*	*5.13*	*0.00*	*0.00*
Jhijhotiya	Hindi-MP	53	G	*9.43*	*1.89*	*45.28*	*33.96*	*5.66*	*0.00*	*3.77*
Jhijhotiya	Hindi-MP	47	B	*8.51*	*2.13*	*31.91*	*46.81*	*8.51*	*0.00*	*2.13*
Deshastha	Marathi	1157	G	*7.35*	*11.58*	*31.55*	*31.63*	*7.52*	*0.00*	*10.37*
Deshastha	Marathi	663	B	*9.05*	*11.31*	*31.37*	*34.69*	*6.79*	*0.00*	*6.79*
Nyogi 6000	Telegu	136	G	*27.21*	*17.65*	*10.29*	*42.65*	*1.47*	*0.00*	*0.74*
Nyogi 6000	Telegu	40	B	*17.50*	*12.50*	*15.00*	*50.00*	*5.00*	*0.00*	*0.00*
Vaidiki	Telegu	142	G	*27.46*	*14.08*	*21.83*	*34.51*	*2.11*	*0.00*	*0.00*
Vaidiki	Telegu	40	B	*20.00*	*12.50*	*15.00*	*47.50*	*5.00*	*0.00*	*0.00*
Smartha	Kannada	277	G	*14.80*	*9.39*	*31.41*	*35.02*	*8.66*	*0.36*	*0.36*
Smartha	Kannada	109	B	*10.09*	*10.09*	*30.28*	*35.78*	*9.17*	*0.00*	*4.59*
Iyer	Tamil	413	G	*25.42*	*15.98*	*8.47*	*31.96*	*16.22*	*0.48*	*1.45*
Iyer	Tamil	180	B	*20.00*	*13.33*	*6.67*	*35.56*	*20.56*	*0.00*	*3.89*

Iyengar	Tamil	170	G	*18.24*	*24.71*	*7.65*	*31.76*	*10.00*	*0.59*	*7.06*
Iyengar	Tamil	42	B	*21.43*	*21.43*	*11.90*	*33.33*	*11.90*	*0.00*	*0.00*
Daivadnya	Konkani	79	G	*3.80*	*13.92*	*8.86*	*22.78*	*29.11*	*2.53*	*18.99*
Daivadnya	Konkani	56	B	*5.36*	*12.50*	*12.50*	*16.07*	*32.14*	*7.14*	*14.29*
Karhade	Marathi	187	G	*2.67*	*13.90*	*22.46*	*20.86*	*17.11*	*0.53*	*22.46*
Karhade	Marathi	130	B	*1.54*	*20.77*	*18.46*	*26.15*	*13.08*	*0.00*	*20.00*
Konkanastha	Marathi	409	G	*3.42*	*9.05*	*50.12*	*8.56*	*20.54*	*0.00*	*8.31*
Konkanastha	Marathi	259	B	*2.70*	*6.56*	*49.03*	*11.20*	*15.44*	*0.00*	*15.06*
Gaur	Hindi-Delhi	958	G	*8.98*	*11.06*	*12.00*	*45.20*	*20.25*	*0.00*	*2.51*
Gaur	Hindi-Delhi	745	B	*10.34*	*11.95*	*10.07*	*43.62*	*21.34*	*0.00*	*2.68*
Gaur	Rajasthani / Marwari	159	G	*7.55*	*1.26*	*13.21*	*62.89*	*10.69*	*0.00*	*4.40*
Gaur	Rajasthani / Marwari	93	B	*9.68*	*1.08*	*9.68*	*64.52*	*11.83*	*0.00*	*3.23*
Tyagi	Hindi-Delhi	172	G	*3.49*	*22.09*	*11.05*	*42.44*	*13.95*	*0.58*	*6.40*
Tyagi	Hindi-Delhi	75	B	*2.67*	*13.33*	*16.00*	*41.33*	*20.00*	*0.00*	*6.67*
Sanadya	Hindi-Delhi	166	G	*17.47*	*1.20*	*16.27*	*57.83*	*7.23*	*0.00*	*0.00*
Sanadya	Hindi-Delhi	103	B	*22.33*	*0.97*	*16.50*	*55.34*	*4.85*	*0.00*	*0.00*
Sanadya	Hindi-MP	115	G	*20.00*	*2.61*	*26.96*	*44.35*	*6.09*	*0.00*	*0.00*
Sanadya	Hindi-MP	64	B	*23.44*	*1.56*	*17.19*	*53.13*	*4.69*	*0.00*	*0.00*
Kanyakubja	Hindi-UP	845	G	*25.44*	*3.31*	*41.18*	*28.99*	*0.95*	*0.00*	*0.12*
Kanyakubja	Hindi-UP	567	B	*25.57*	*4.06*	*35.63*	*33.86*	*0.88*	*0.00*	*0.00*
Saryuparin	Hindi-UP	815	G	*4.42*	*19.88*	*43.68*	*30.43*	*1.47*	*0.00*	*0.12*
Saryuparin	Hindi-UP	501	B	*8.58*	*14.77*	*45.31*	*29.34*	*1.80*	*0.00*	*0.20*
Bhumihar	Hindi-UP	603	G	*8.13*	*15.59*	*46.60*	*24.88*	*4.81*	*0.00*	*0.00*
Bhumihar	Hindi-UP	290	B	*10.00*	*15.86*	*40.34*	*28.28*	*5.52*	*0.00*	*0.00*
Maithil	Bihari	1122	G	*3.39*	*28.70*	*58.56*	*8.73*	*0.62*	*0.00*	*0.00*
Maithil	Bihari	420	B	*2.86*	*30.95*	*56.67*	*8.81*	*0.71*	*0.00*	*0.00*
All Brahmin	Bengali	1981	G	*0.76*	*1.36*	*67.19*	*29.38*	*1.16*	*0.00*	*0.15*
All Brahmin	Bengali	1313	B	*0.69*	*1.68*	*64.43*	*32.37*	*0.76*	*0.00*	*0.08*

All Brahmin	Oriya	905	G	*7.51*	*0.33*	*30.50*	*51.82*	*4.86*	*1.22*	*3.76*
All Brahmin	Oriya	331	B	*9.06*	*0.91*	*29.61*	*49.55*	*6.04*	*1.51*	*3.32*
All Brahmin	Telegu	628	G	*25.80*	*15.45*	*18.15*	*36.62*	*3.18*	*0.00*	*0.80*
All Brahmin	Telegu	153	B	*21.57*	*12.42*	*15.69*	*45.75*	*4.58*	*0.00*	*0.00*
All Brahmin	Marathi	3314	G	*7.42*	*13.25*	*31.41*	*25.50*	*12.64*	*0.12*	*9.66*
All Brahmin	Marathi	1853	B	*8.15*	*13.01*	*30.98*	*26.82*	*12.30*	*0.05*	*8.69*
All Brahmin	Hindi-MP	1640	G	*14.51*	*11.77*	*33.23*	*36.04*	*3.60*	*0.00*	*0.85*
All Brahmin	Hindi-MP	954	B	*14.78*	*13.10*	*32.29*	*35.01*	*3.35*	*0.00*	*1.47*
All Brahmin	Gujarati / Kutchi	991	G	*10.60*	*7.77*	*23.71*	*43.19*	*12.71*	*0.71*	*1.31*
All Brahmin	Gujarati / Kutchi	580	B	*10.52*	*8.97*	*18.79*	*45.86*	*14.31*	*0.69*	*0.86*
All Brahmin	Kannada	1070	G	*10.37*	*13.93*	*29.16*	*29.53*	*11.68*	*0.47*	*4.86*
All Brahmin	Kannada	416	B	*10.10*	*13.70*	*25.96*	*28.13*	*15.63*	*0.48*	*6.01*
All Brahmin	Rajasthani / Marwari	463	G	*7.56*	*5.83*	*19.22*	*56.16*	*8.21*	*0.00*	*3.02*
All Brahmin	Rajasthani / Marwari	243	B	*11.93*	*4.94*	*15.64*	*56.38*	*9.05*	*0.00*	*2.06*

*G=groom; B=bride; Gotra groups: Vasishta (Vasisht, Parashar, Upamanyu, Kaundinya); Bhargava (Bhargava, Jamadagni, Vatsa, Srivats); Kashyap (Kashyap, Sandilya); Angiras (Bharadwaj, Gautam); Vishwamitra (Vishwamitra, Kausika); Kutsa (Kutsa, Kaunsha); Source: www.Jeevansathi.com (downloaded October 2012).

Table S26. **Tribal gene introgression from NW entry points into South Asia**. M176 (L1), M357 (L3), M241 (J2b2), M410 (J2a), M17 (R1a1) and M124 (R2) SNPs were included. Based on published genetic analyses of various communities, these SNPs together account for >90% of post-neolithic Y-haplogroup introgression from northwest entry points into South Asia [reference C]. M69 was used an indigenous control.

				PERCENT INCIDENCE OF Y-HAPLOGROUP						
**CLUSTER***	**CENSUS****	**SAMPLE (n)**	**TRIBES**	**M176**	**M357**	**M241**	**M410**	**M17**	**M124**	**M69**
INDIGENOUS	>5%	679	16	1.6	0.0	1.1	0.8	7.0	2.3	44.3
OCP-CH	>5%	240	9	4.3	0.0	4.2	0.0	28.4	21.9	30.7
BRW	>5%	465	10	21.9	0.0	22.9	0.0	17.7	3.1	16.0
IRANIC	>5%	87	4	8.3	10.4	8.4	4.7	28.8	14.3	12.3
BORDERLANDS	n.a.	71	3	8.0	4.3	3.2	11.7	34.8	9.6	<5

* Indigenous (MP Gond, UP Gond, Koya, Bagdi, Muria, Kurumba, Halba, Santhal, Koya Dora, Hallaki, Kamar, Kharia, Bhil, Irula, Kuruba, Saharia); OCP-CH (Chamar, Chenchu, Naidu, Madiga, Ao Naga, Satnami, Dhangar, Vaish, Maratha); BRW (Yadava, UP Kol, Meghawal, Lodhi, Vellalar, Kallar, Velama, Ambalakalar, Sourashtran, Mala); Iranic (Rajput, Gurjar, Khatri, Pallan); Borderlands (Baloch, Brahui, Sindhi).
** Indian census of 2010 and 1891. Each sample cluster accounts for >5% of the population in both census numbers, suggesting antiquity of the underlying introgression events.

REFERENCES:

[A] Wells RS, Yuldasheva N, Ruzibakiev R, Underhill PA, Evseeva I, Blue-Smith J, Jin L, Su B, Pitchappan R, Shanmugalakshmi S, Balakrishnan K, Read M, Pearson NM, Zerjal T, Webster MT, Zholoshvili I, Jamarjashvili E, Gambarov S, Nikbin B, Dostiev A, Aknazarov O, Zalloua P, Tsoy I, Kitaev M, Mirrakhimov M, Chariev A, Bodmer WF. (2001) The Eurasian heartland: a continental perspective on Y-chromosome diversity. Proc Natl Acad Sci U S A. 98(18): 10244-10249.

[B] Kivisild T, Rootsi S, Metspalu M, Mastana S, Kaldma K, Parik J, Metspalu E, Adojaan M, Tolk HV, Stepanov V, Gölge M, Usanga E, Papiha SS, Cinnioğlu C, King R, Cavalli-Sforza L, Underhill PA, Villems R. (2003) The genetic heritage of the earliest settlers persists both in Indian tribal and caste populations. Am J Hum Genet. 72(2): 313-332.

[C] Sengupta S, Zhivotovsky LA, King R, Mehdi SQ, Edmonds CA, Chow CE, Lin AA, Mitra M, Sil SK, Ramesh A, Usha Rani MV, Thakur CM, Cavalli-Sforza LL, Majumder PP, Underhill PA. (2006) Polarity and temporality of high-resolution y-chromosome distributions in India identify both indigenous and exogenous expansions and reveal minor genetic influence of Central Asian pastoralists. *Am J Hum Genet.* 78(2): 202-221.

[D] Reich D, Thangaraj K, Patterson N, Price AL, Singh L. (2009) Reconstructing Indian population history. Nature. 461(7263): 489-494.

[E] Sharma S, Rai E, Sharma P, Jena M, Singh S, Darvishi K, Bhat AK, Bhanwer AJ, Tiwari PK, Bamezai RN. (2009) The Indian origin of paternal haplogroup R1a1* substantiates the autochthonous origin of Brahmins and the caste system. J Hum Genet. 54(1): 47-55.

File S27. **Religious traditions in medieval Saurashtra.**

A.

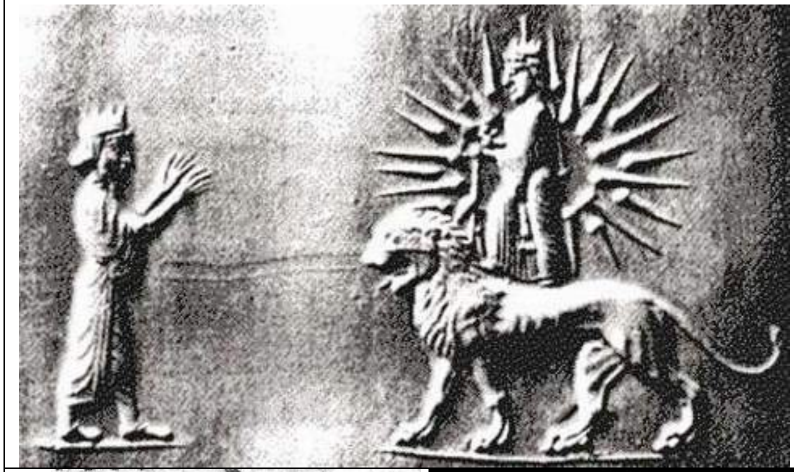

Lion, sun and water symbols. A. Achaemenid emperor Artaxerxes II (4thC BCE) worshipping lion and Mithra (rising sun) and Anahita water deity. B. Left, Achaemenid symbol showing ancient sun icons, eagle wings with Mithra sun disks. Middle, coin of last Seljuk king (12th C CE). Right, 12thC CE Goan Kadamba issue with stylyzed lion and Mithra sun disk. The lion-sun is an enduring Persian royal symbol.

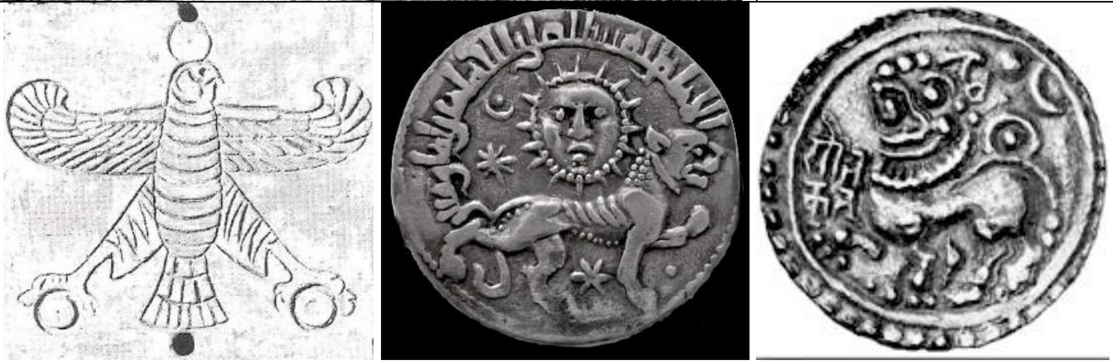

B.

Sindh and Saurashtra have long represented a contiguous cultural domain [main text]. The coastal Sindh-Sorath region during the Harappan period of the 3rd millennium BCE has left us artifacts suggesting proto-Shaivite-Tantric religious beliefs [Supplementary files 2.07, 2.08] perhaps the predecessors of later western grassroots traditions rooted in the worship of various local tantric mother goddesses such as Chamunda and Ambaji — later co-opted into aspects of Durga — and the Pasupata-Kapalika-Nath traditions of early Shaivism. The Pasupata cult began in Somnath on the coast of Saurashtra. The Kapalika and Nath traditions also originated in Gujarat [3A]. In the centuries preceding the Common Era, sponsored by the great Mauryan Emperors Chadragupta and Ashoka, Jainism and Buddhism were grafted upon this substrate from the east. In Saurashtra, Girnar and Vallabhi became important centers, especially for heretical sects of these religions, as attested by historical councils and the testimony of the Chinese traveller Hwang Zhang in the 7th century [3B]. It appears that the western mercantile seaboard extending from Sindh and Gujarat to Cape Comorin remained largely outside of Indo-Gangetic orthodox Brahmanical influence until late medieval times [3C]. Although sun cults, totems and heroes would ultimately be absorbed into the Vaishnava tradition, Surya (Aditya) worship was clearly a distinct and influential tradition in early medieval Gujarat, as the 8th century Shankaracharya five-god Smartha prescription indicates.

From a survey [3D] of religious and totemic symbols on coins issued by independent tribes located in the Indus-Yamuna region during the Maurya-Kushana interregnum (2nd century BCE to 1st century CE) one may distinguish three non-overlapping cultural traditions: towards the easterly side, running north-south from Himachal Pradesh to Rajasthan (Kulutas, Kunindas, Vemakis,

Yaudheyas, Agras, Arjunayanas, Uddehikas, southern Sibis) one finds traditional symbols of Buddhist (Chaitya, Triratna, Tree-in-Railing) and Elephant-Humped Bull (Bull, taurine symbols) traditions. The latter has traditionally been linked to the Kapisa-Pushkalavati corridor i.e. Khyber Pass corridor [3E] and may have anchored the mainstream Shaiva-Oesho traditions patronized by the Kushana emperors and associated with the bull, a lunar symbol [3F].

The third distinct tribal numismatic culture, on the other hand (lion and water/river symbols, probably a Mitra-Varuna tradition, see below) is the most northwesterly and largely located between the Jhelum and Ravi tributaries of the Indus (Gujrat district in the Punjab) and other areas later associated with mid-1st millennium CE Gurjar settlements, including Marwar. Ancient tribes using these symbols on their coinage (Malavas, Pauravas, Audumbaras) are notable for dual attested locations north and south of the Bolan Pass. Generally speaking, these locations are close to entry points from Arachosia to Sindh, either through the Bolan Pass (Sindh-Gujarat) or the Tochi Valley (Sindh-Punjab). A southern branch of Audumbaras, for example is known from Gujarat (Kutch). They were Shaivites, based on the names of their kings and the evidence of Hwang Zhang, a Chinese pilgrim, who visited it in 639 CE [3B]. Malavas are attested both in the Punjab and in Nagaur near Marwar (an early Gurjar kingdom) and from the Saka / Gurjar domains between Brighukacha and Ujjain, which came to be known as Malwa [3H]. The Sarasvata tribe of Arachosia is not known to have issued coinage, but its provenance may be associated with the naming of Sarasvati rivers in the Punjab and Gujarat. At least some of these Iranic tribes may have used the Krta-Malava era beginning in 57-58 BCE (possibly associated with Vonones or Azes I in Arachosia; [3I]). This type of reckoning was later adopted by the Gurjara-Pratihara medieval empire. Meanwhile, by contrast, in Madhyadesa coinage of the same period, lion symbols are rare and the water/river symbol is sparingly used (though at least one example is known from Erikachcha of the Dasarna kingdom) [3P].

In order to understand the significance of the lion-water symbol complex in the northwestern regions closest to Afghanistan just prior to the start of the Common Era one must first consider the political context of the region comprising Arachosia, Sindh and Gujarat. These areas were ruled as satrapies of Achaemenid Persia in the mid-first millennium BCE. After the collapse of the Mauryan Empire in the 2nd century BCE, the tradition of rule by Afghan-Iranic overlords, including Indo-Greek, Indo-Scythian and Indo-Parthian dynasties, became a tradition to continue for another millennium. In the coinage of these dynasties prior to the Common Era, the lion symbol was clearly associated with Iranic Arachosia (Harahvati) and not Indian Gandhara [Supplementary File 2.10]. The emblematic use of Persian fire altars on coinage of Sindh and Gujarat continued until the latter part of the 1st millennium CE, and became a standard feature of coins issued by the Gurjar kingdoms.

For the significance of the lion-water symbols themselves one must therefore search within this Iranic cultural tradition. Illustration above shows examples of Persian sun and water icons beginning with the Achaemenids. As Mary Boyce has argued [3Q], the reign of Artaxerxes II (404-359 BCE) saw great turmoil between royal house and religious orthodoxy in the emperor's attempts to replace Varuna of the Mithra-Varuna tradition with the popular goddess Anahita, the Arachosian (Harahvati) deity of the waters, the Persian equivalent of the India Sarasvati. Western Kshatrapa coinage of the early centuries CE in Gujarat and Malwa employed a standardized set of numismatic symbols: Buddhist chaitya, lunar and sun symbols, and river symbol below. Presumably this set represented the major belief systems in Gujarat and Saurashtra during that period. By the 6th and 7th centuries, after the influx of Hephthalite influences through ruling Maitraka (Saurashtra), Rai (Sindh), Mihirkula (Eran) and Zunbil (Zabulistan) dynasties, northwesterly

populations carried syncretic sun worship and Shaiva traditions, *not* iconographically associated with the lunar bull. Wink has suggested that the Zamindawar (Zamin-i-Dawar) cult of Zun was a Hephthalite fusion of Shiva and Mihira sun worship [3O]. It is this syncretic substrate, along with tantric goddess cults, the heretical Buddhist sects and the Jaina traditions mentioned above, that anchored the grassroots belief systems of medieval Saurashtra. Similar beliefs predominated in Sindh. (For a review of the religious traditions in medieval Sindh, see reference 3J.) Within this catholicity, in addition to the Buddhism and Zun-Shaivism patronized by the Maitraka rulers in Vallabhi in their land grants [3B], one may distinguish three religious traditions of particular relevance to the Brahmins who may have migrated south from Saurashtra to Salcete province in 740 CE:

The Bhagavata Tradition: In the 1st millennium BCE, the Mahabharata places the tribes of Abhira and Sura in adjacent regions of southern Sindh and Saurashtra (Saura=sun worshipper, rashtra=country). Classical geographers refer to Sindh as Abiria [3N]. Vasudeva Krishna, the Vrishni Bhoja hero is revered by the Abhira, of which the Bhojas are a major branch. This "Bhagavata" worship of Vasudeva and his brother Balarama (Heracles, in the Hellenistic equivalent), the earliest known Vaishnavite tradition, is attested in coinage of Arachosia (7 of 22 numismatic series of Arachosia mints versus 0 of 52 series from Gandhara mints; as a control, the signature humped bull of Pushkalavati is found on 6 Gandhara series and none of the Arachosia series) between the 2nd century BCE and the 1st century CE [Supplemental File 2.09]. As the 113 BCE Heliodoros pillar erected by the ambassador from Antialcidas of Arachosia makes clear, Bhagavata was popular in Zabulistan. Dwarka, in Saurashtra, was the legendary home of Vasudeva Krishna and the Vrishni Bhojas.

The Magian Sun Priest Tradition: According to the Bhavishya Purana, Krishna's son Samba brought Iranic Magian sun priests from Zabulistan (Sakadwipa) to Multan and Dwarka, as these were the only priests qualified to serve in sun temples. At the turn of the Common Era, Parthian nobles of the Suren-Pahlav clan based in Seistan controlled Arachosia. The Gondophares (Guduvaraha) dynasty of rulers may have belonged to this clan or, alternatively, to the house of Waraz. Suren and Waraz were the easternmost royal families of Parthia [3K]. The Magian sun priests of this region, Sakadwipa, according to Indian tradition, married Bhoja women and came to be known as Bhojaka. Bhojaka priests serve in both Surya and Jaina temples. Interestingly, Bhoj-wordstem toponyms show a remarkably similar distribution to the Indian provenance of early-common-era Parthian terra cotta votive tanks in the archeological record, including many Jaina Tirthas but not Buddhist monasteries [Supplemental File 2.04]. Magian sun priests accompanied Gurjar and Hephthalite Iranic tribes that entered western India in the 5th and 6th centuries CE. Among these arrivals were the Varaha (wild boar) totem tribes, precursors of the great Chalukya and Gurjara-Pratihara clans of India. These were almost certainly related to the Eastern Parthian-Hephthalite House of Waraz (Varaz=boar) [3L]. Their arrival in the western coastal towns of Saurashtra in the late fifth-early sixth century is memorialized by a unique sun temple architecture found around Dwarka, Porbandar and Somnath-Patan. Similar temple architecture can be seen in the early Chalukya capital of Aihole, Karnataka (6th century CE) and in the great Salt Range Temple complex of the same vintage [Supplemental Files 3.02 and 3.03]. The medieval Garulaka tribe of Saurashtra, which ruled coastal Saurashtra, may have been a Waraz (Varaha) tribe, as some of its kings took the title of Varahadasa. Some Magian sun priests were Brahman-ized in the 7th-8th centuries. Supplemental files 3.02-3.04 provide additional detail.

An Emerging Goudapada Brahmanic Tradition: The arrival of Brahmins in Salcete in 740 CE coincided with the founding of the first Math (monastery) of the emerging Advaita sect known

as Goudapada close to their new settlement at Kushasthali in Salcete. As this Brahmin community came to be known as Goud Saraswat Brahmins, it is likely that the name came from their adherence to this sect. (Other explanations for the name “Goud” have been offered, notably “from the north”, or “from Bengal”, but these explanations do not seem as persuasive.) Goudapada’s Advaita teachings became dominant in the western corridor of the subcontinent after Sankaracharya (late 8th century CE). The GSB were ardent followers of Sankaracharya’s Smartha branch of Vedanta for over 500 years, until the arrival of Madhavacharya, when some GSB converted to Vaishnavism.

If these Goud Brahmins did indeed bring their Advaita philosophy from Saurashtra to Salcete in 740 CE, one may ask about philosophical antecedents. In the earliest Goudapada work independent of the great Sankaracharya, the Gaudapadiya-Karika, the fourth prakarna contains a remarkable philosophical synthesis of the Buddhist Madhyamaka and Yogacara schools, which may well precede the Mahayana’s own syncretism of the two schools in the works of Vimuktisena (6th century CE) and Santaraksita (7th century CE). Even more remarkable, this work was written by a Vedantin, not a Buddhist [3M]. The Maitraka capital, Vallabhi, is the most likely 6th century center of learning to fit the sophistication, intellectual independence and catholicity implied by this work. If the GSB did, in fact live in Saurashtra in the 6th century, it is highly likely that their schooling and employment was in the city of Vallabhi.

REFERENCES

3A. White DG (1996) *The Alchemical Body: Siddha Traditions in Medieval India*. U. Chicago Press, p. 97.
3B. Rongxi L. (tr.) (1996) *The Great Tang Dynasty Record of the Western Regions*. Numata Center Buddhist Translation, Berkeley, CA.
3C. Thapar R. (1978) *Ancient Indian Social History: Some Interpretations*. Orient Blackswan Publications.
3D. Handa D. (2007) *Tribal Coins of Ancient India*. Aryan Books International, New Delhi.
3E. Sagar KC (1992) *Foreign Influence on Ancient India*. Northern Book Centre, New Delhi. p. 98.
3F. Pal P (1986) *Indian Sculpture*. Vol. I. UC Press, Los Angeles. p. 77.
3H. Viyogi N and Ansari MA (2010) *History of the Later Harappans and Silpakara Movement*. Kalpaz Publications, Delhi. p. 231.
3I. Sircar DC (1965) *Indian Epigraphy*. Motilal Banarsidass Publishers, Delhi. p. 257.
3J. Maclean DN (1989) *Religion and Society in Arab Sindh*. EJ Brill, Leiden.
3K. Curtis VS and Stewart S eds. (2007) *The Age of the Parthians*. IB Tauris & Co, London. p. 30.
3L. Spawforth AJS ed. (2007) *The Court and Court Society in Ancient Monarchies*. Cambridge University Press, p.66.
3M. King R (1995) *Early Advaita Vedanta and Buddhism: The Mahayana Context of the Gaudapadiya-Karika*. SUNY Press. p. 44.
3N. Schoff WH. (1912) *The Periplus of the Erythraean Sea: Travel and Trade in the Indian Ocean by a Merchant of the First Century*. Munshiram Manoharlal Publ., 2001.
3O. Wink A. (2002) *Al-Hind: the Making of the Indo-Islamic World*. Brill Academic Publishers.
3P. Bhandare S. (2006) Numismatics and History: The Maurya-Gupta Interlude. In Olivelle P. (ed) *Between the Empires: Society in India 300 BCE to 400 CE*. Oxford University Press, pp. 67-112.
3Q. Boyce M (1982) *A History of Zoroastrianism*, Vol II. EJ Brill, Leiden, p.247.

File S28. **Attested Mihira clan locations (5th-8th centuries CE).**

Attested locations of Iranic Hephthalite family clans and dynasties with affinity for Mihira (Mithra, sun) worship and Waraz (boar; Sanskrit "Varaha") totem. Coinage typically shows Sassanid-style fire altar on reverse side. These rulers typically adopted the name "Aditya" (sun god, Surya) or "Mihir" (Mithra) in their royal titles, a practice significantly less common in other contemporary lineages of the subcontinent. Varaha totemic orientation is revealed in royal crests (e.g. Chalukyas) and royal names such as Varahadasa (devotee of the Boar), used by Garulaka kings. A major influx of Magian sun priests into the subcontinent (later named Maga Brahmins, Bhojakas, Sakaldwipi Brahmins) coincided with the arrival of Mihira clans.

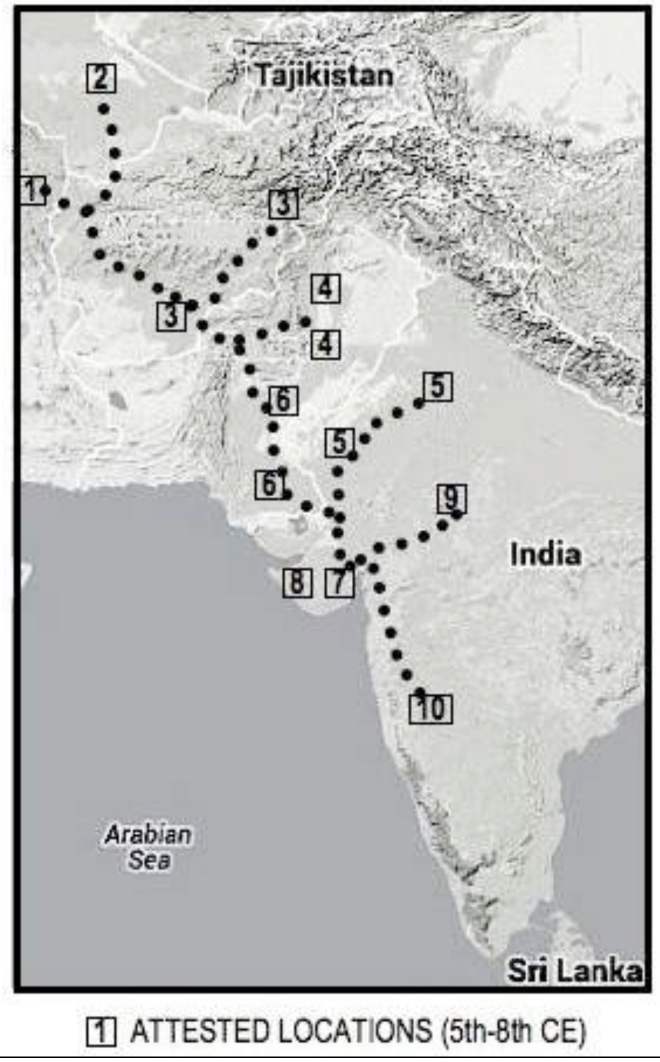

1: Sassanid-era (224-651 CE) feudal aristocracy, the House of Waraz, Eastern Khurasan. (Waraz=Varaz=Varaha=Boar)

2. King Waraz, last bastion of Hephthalite power in Bactria (479-563 CE) defeated by the Turks and Persians in 557 CE.

3. Zabuli-Kabuli Nezak Shahis 565-879 CE.

4. Varaha/Chalukya-style pre-Nagara temples 6th-7th CE. (Supplemental File 3.02). Multan (Mitravana) sun temple.

5. Gurjara-Pratihara (Bhinmal, Kannauj) 550-1036 CE.

6. Pre-Arab Rai kings of Sindh 489-690 CE.

7. Maitrakas of Vallabhi 475-767 CE.

8. Garulakas of western Saurashtra 480-738 CE.

9. Toramana / Mihirakula 480-528 CE.

10. Early western Chalukyas, Aihole, Karnataka 543-753 CE.

Reference: Humbach H (1978) Mithra in India and the Hinduized Magi. In: *Acta Iranica* Duchesne-Guillemin ed., Bibliotheque Pahlavi, Tehran-Liege. pp. 229-254.

Table S29. **Varaha (Chalukya) style Aditya temples.** A unique transitional pre-Nagara style of sun temple associated with 6th-7th century CE tribes associated with wild boar (Varaha, Waraz) worship has been described [reference 1, below].

Location	State	Vintage	N	E
SALT RANGE GROUP:				
Katas	Punjab	6th CE	32.73	72.16
Kafirkot	Punjab	6th-7th CE	34.01	72.45
Bilot	Punjab	7th CE	32.26	71.08
Mari Indus	Punjab	8th CE	32.95	71.56
Kallar Kahar	Punjab	late 8th CE	32.78	72.70
Amb Sharif	Punjab	9th CE	32.51	71.94
SAURASHTRA GROUP:				
Gop, Dwarka	Gujarat	6th CE (early)	22.23	68.97
Bhanasara, Porbandar	Gujarat	625-650 CE	21.62	69.60
Dhank	Gujarat	6th-7th CE	21.77	70.13
Somnath	Gujarat	6th-7th CE	20.89	70.41
Visavada, Junagadh	Gujarat	6th-7th CE	21.38	70.68
Bileswar, Junagadh	Gujarat	late 7th CE	21.68	69.74
Pindara	Gujarat	6th-7th CE	22.25	69.26
Navi-Dhrewad	Gujarat	6th-7th CE	22.14	69.07
Son-Kansari, Ghumli	Gujarat	6th-7th CE	21.77	70.13
Harshadmata, Miyani	Gujarat	6th-7th CE	21.84	69.38
Kin-derkheda, Porbandar	Gujarat	6th-7th CE	21.81	69.56
Khimeswar, Porbandar	Gujarat	6th-7th CE	21.62	69.60
Kadwar, Prabhaj-Patan	Gujarat	late 7th	20.85	70.45
Sutrapada [transitional]	Gujarat	early 8th CE	20.85	70.48
CHALUKYA GROUP:				
Aihole	Karnataka	6th CE	16.02	75.87

REFERENCES:
1. Ghosh A ed. (1961) Indian Archeology 1960-61: A Review. Janpath, New Delhi. p.75.
2. Meister MW (1996) Temples along the Indus. *Expedition* 38(3): 41-54.

Table S30. **Ramnath, Manganath, Siddhanath temples (Gujarat).** The states of Goa and neighboring GSB diaspora states of Maharashtra and Karnataka are italicized. The Manganath temple at Kushasthali, Goa is now located at Priol, Goa under the name Mangeshi. At the time of Portuguese conquest in the 16th century, records show this deity's name was Manganath. The medieval use of the –nath suffix to indicate Shiva (instead of the more conventional –eshwar) is largely limited to Gujarat.

Location of Ramnath Temples in India

STATE	*TOWN*	*N*	*E*
**GUJARAT**	Bilkha	21.44	70.61
**GUJARAT**	Jetpur, Rajkot	21.76	70.62
**GUJARAT**	Gondal, Rajkot	21.98	70.74
**GUJARAT**	Sihor, Bhavnagar	21.71	71.96
**GUJARAT**	Rampara, Junagadh	20.96	70.96
**GUJARAT**	Petlad, Anand	22.49	72.78
**GUJARAT**	Jhagadia, Bhalod	21.71	73.15
**GUJARAT**	Surat	21.19	72.83
**GUJARAT**	Bhuj	23.27	69.67
**GUJARAT**	Vallabhipur	21.89	71.88
**GUJARAT**	Ahmedabad, Manek Chowk	23.03	72.58
**GUJARAT**	Khambaliya	22.21	69.65
**GUJARAT**	Borsad	22.42	72.90
**GUJARAT**	Makarpura, Vadodara	22.30	73.20
MAHARASHTRA	Karanaja, Badona	20.58	77.65
MAHARASHTRA	Atpadi, Sangli	17.26	74.57
GOA	Ramnathim, Ponda	15.39	73.98
KARNATAKA	Majali, Karwar	14.90	74.10
KARNATAKA	Hankon, Karwar	14.89	74.19
KARNATAKA	Madgeri, Kumta	14.43	74.41
KARNATAKA	Asnoti, Karwar	14.87	74.13
KARNATAKA	Bhaire, Karwar	14.99	74.25
KARNATAKA	Ulga, Karwar	14.88	74.24
KARNATAKA	Karkal, Udipi	13.20	74.98
**CHHATTISGARH**	Kanker	20.27	81.49
**ODISHA**	Boudh	20.82	84.32
**ODISHA**	Satpada, Balugaon	19.73	85.65
**UTTAR PRADESH**	Varanasi	25.68	82.96
**TAMILNADU**	Rameshwaram	9.29	79.32

Location of Manganath Temples in India

STATE	*TOWN*	*N*	*E*
**GUJARAT**	Mangnath Pipali village	21.41	70.69
**GUJARAT**	Junagadh	21.52	70.47
**GUJARAT**	Nani Monpari	21.36	70.64
**MADHYA PRADESH**	Shajapur	23.43	76.27

Location of Siddhanath Temples in India

STATE	*TOWN*	*N*	*E*
**GUJARAT**	Dholka	22.74	72.35
**GUJARAT**	Munjlav, Olpad	21.32	72.69
**GUJARAT**	Surat	21.11	72.70
**GUJARAT**	Jamnagar	22.47	70.07
**GUJARAT**	Dwarka	22.24	68.95
**GUJARAT**	Somnath (Durgakut Ganesh)	20.89	70.41
**GUJARAT**	Girnar (Revati Ganesh)	21.49	70.53
**GUJARAT**	Chikhli, Navsari	20.75	73.06
**GUJARAT**	Baroda	22.31	73.21
**GUJARAT**	Bharuch	21.71	72.99
**GUJARAT**	Kherva	23.54	72.44
GOA	*Bori, Ponda*	15.37	74.04
MAHARASHTRA	Mhaswad	17.64	74.79
MAHARASHTRA	Kharsundi	17.34	74.88
MAHARASHTRA	Bhood	17.64	74.79
MAHARASHTRA	Renavi	17.27	74.65
MAHARASHTRA	Kanamadi	16.83	75.73
MAHARASHTRA	Maan	17.67	74.50
**MADHYA PRADESH**	Nemawar	22.50	76.97
**MADHYA PRADESH**	Omkareshwar	22.24	76.15
**RAJASTHAN**	Jodhpur	26.28	73.02
**HIMACHAL PRADESH**	Baijnath	32.05	76.65
**UTTAR PRADESH**	Kanpur	26.42	80.42
**UTTAR PRADESH**	Lucknow	26.85	80.95
**UTTAR PRADESH**	Zamania, Ghazipur	25.50	83.62
**UTTAR PRADESH**	Jaunpur	25.45	82.52
**UTTAR PRADESH**	Bahraich	27.58	81.59
**UTTAR PRADESH**	Jajmau	26.46	80.35
**BIHAR**	Bahadurpur, Rafiganj	24.85	84.55
**BIHAR**	Fatuha	25.52	85.32

Table S31. **Male deities in Salcete temples in 1567**. Dark rectangles signify presence of deity, based on inventory created priror to destruction of 300+ temples by the Portuguese Inquisition in 1567.

TOWN / VILLAGE	Narayana	Ravalnath	Other Vishnu	Siddhanath / Ganesh	Ishvar / Mahadev	Bhairava	Other Shiva
Guirdolim	■	■					
Kudtari	■	■		■	■		Chandranath
Macazana	■	■			■		
Mormugao							Vagnath
Issorcim							
Dabolim					■		
Vaddem	■				■		
Chicalim	■				■		
Sancoale	■		Narasimha		■		
Kushasthali	■				■		Manganath
Keloshi	■			■	■		
Nagoa	■	■			■		
Verna	■				■		
Lotli	■		Vamana	■			Ramnath
Raia	■				■		
Camorlim					■	■	
Racaim	■		Trivikrama		■		
Margao	■		Damodar		■		
Dicarpale							
Davorlim							
Pale					■		
Velcao					■		
Cansaulim					■		Naganath
Arossim					■		
Utorda							
Majorda					■		
Calata							
Seraulim					■		
Betalbatim				■	■		
Gandaulim							
Colva	■	■			■	■	
Benaulim	■				■	■	
Telaulim				■			

Orlim	■						
Sernabatim				■			
Varca					■	■	
Deussua						■	Loknath
Carmona					■		
Chinchinim							
Dramapur	■				■	■	
Sarzora							Naganath
Cuncolim					■		
Veroda					■		
Duncolim					■		
Assolna							
Velim							
Ambelim					■		

REFERENCE: Pissurlencar P. (1952) Tombo das Rendas que Sua Magestade tem nas terras de Salcete. *Boletim do Instituto Vasco da Gama* 68: 19-79.

Table S32. **First names of 16th century Lotli Brahmins**. The ratio of Shaivite to Vaishnavite first names for village council member males is shown.

FIRST NAMES

	n	*Shaiva::Vaishnav*
Guirdolim-Macazana	13	0.00
**Curtorim**	31	**1.79**
**Raia**	57	0.88
**Loutolim**	15	0.79
**Quelossim**	10	0.69
**Cortalim**	11	**3.70**
**Sancoale**	9	**1.39**
Chicalim-Vaddem	12	0.00
Benaulim	25	1.19
Margao	25	0.60
VERNA-NAGOA	42	1.19
**ALL BRAHMIN**	250	1.00

REFERENCE: Pissurlencar P. (1952) Tombo das Rendas que Sua Magestade tem nas terras de Salcete. *Boletim do Instituto Vasco da Gama* 68: 19-79.

Table S33. **West-east gotra gradient.** Data are from Suplementary File 2.13. Bride and groom data for individual gotras in each community agreed closely (r2=0.94), providing an internal control. Based on this Distribution it is possible to map the centroid of overall gotra incidence for any Brahmin community or subgroup. The centroid thus calculated for present day Kamat GSB is 971 km east of the corresponding centroid for GSB carrying the surname of Pai (data not shown). Although this does not prove a more easterly (average) origin for Kamat GSB families, it is nevertheless consistent with such a claim.

BRAHMIN GOTRA	WESTERN	CENTRAL	EASTERN
Mother Tongues	Konkani<br>Gujarati<br>Marathi	Hindi-UP<br>Hindi-MP	Bengali<br>Bihari
Sample n (G)	5307	7521	4852
Sample n (B)	3132	4147	2480
Pct Kashyap (G)	7.26	18.15	20.97
Pct Kashyap (B)	7.83	18.13	20.12
Pct Kaushik (G)	22.55	12.53	3.64
Pct Kaushik (B)	23.60	11.84	2.77
Pct Kutsa (G)	33.33	0.00	0.00
Pct Kutsa (B)	32.99	0.52	0.00
Distribution [G]	11.37	65.62	77.32
Distribution [B]	11.24	65.78	77.36

G=grooms; B=brides; Distribution=Kaushik+Kutsa-Kashyap; Source: Jeevansathi.com; downloaded October 2012.

Table S34. **Maithili cohort gotras (7th century).** Bhaskaravarman land grant to 114 Maithili Brahmins in 600-650 CE in the Kamarupa kingdom.

Vedic School	*Gotra*	*Donee #*
Vajasaneya (Yajur)	Alambayana	11
Vajasaneya (Yajur)	Alambayana	1
Vajasaneya (Yajur)	Anjirasa	1
Chhandoga (Sama)	Asvalayana	1
Chhandoga (Sama)	Bharadvaja	2
Chhandoga (Sama)	Bharadvaja	1
Vajasaneya (Yajur)	Bharadvaja	5
Vajasaneya (Yajur)	Bharadvaja	6
Vajasaneya (Yajur)	Bharadvaja	1
Bahvrichya (Rigvedin)	Bharadvaja	1
Vajasaneya (Yajur)	Bharadvaja	1
Vajasaneya (Yajur)	Bharadvaja	1
Vajasaneya (Yajur)	Bharadvaja	1
Vajasaneya (Yajur)	Gargya	1
Bahvrichya (Rigvedin)	Gauratreya	10
Chhandoga (Sama)	Gautama	2
Vajasaneya (Yajur)	Gautama	2
Bahvrichya (Rigvedin)	Gautama	2
Vajasaneya (Yajur)	Gautama	1
Chhandoga (Sama)	Gautama	1
Vajasaneya (Yajur)	Gautama	1
Vajasaneya (Yajur)	Gautama/Kasyapa	2
Vajasaneya (Yajur)	Kasyapa	2
Bahvrichya (Rigvedin)	Kasyapa	1
Taittiriya (Yajur)	Kasyapa	1
Vajasaneya (Yajur)	Kasyapa	1
Chhandoga (Sama)	Katyayana	3
Charakya (Yajur)	Katyayana	1
Vajasaneya (Yajur)	Kaundinya	1
Bahvrichya (Rigvedin)	Kausika	1
Bahvrichya (Rigvedin)	Kausika	2
Bahvrichya (Rigvedin)	Kausika	2
Vajasaneya (Yajur)	Krishnatreya	1
Vajasaneya (Yajur)	Maudgalya	2
Chhandoga (Sama)	Pankalya	1
Charakya (Yajur)	Parasarya	1
Bahvrichya (Rigvedin)	Parasarya	1
Vajasaneya (Yajur)	Prachetasa	3
Vajasaneya (Yajur)	Sakatayana	1
Vajasaneya (Yajur)	Salankayana	1
Vajasaneya (Yajur)	Sandilya	1
Vajasaneya (Yajur)	Saunaka	7
Bahvrichya (Rigvedin)	Saunaka	9

Bahvrichya (Rigvedin)	Varaha	1
Bahvrichya (Rigvedin)	Varaha	1
Bahvrichya (Rigvedin)	Varhaspatya	7
Charakya (Yajur)	Vatsa	2
Bahvrichya (Rigvedin)	Vatsya	1
Bahvrichya (Rigvedin)	Yaska	3
Vajasaneya (Yajur)	Yaska	1

Reference: XIX Epigraphica Indica pp. 115-125.

Table S35. **Orissa donee gotra listing (4th-12th centuries).** Summary tabulation of land grants: gotras of 253 Brahmin donees receiving grants from the kings of Orissa.

Gotra	***301-700 CE***	***701-1000 CE***	***1001-1150 CE***
Agasti	0	0	1
Atreya	15	1	*
Aupamanya	1	0	1
Autathya	0	0	3
Bharadvaja	9	20	12
Barhaspatya	0	0	1
Bhargava	1	0	0
Daksa	0	1	0
Dalbhya	0	0	1
Devarata	1	0	0
Dharini	1	0	0
Gargya	1	0	3
Gautama	2	1	4
Harita	0	1	1
Jatukarnya	0	2	1
Kamakayana	1	1	0
Kanva	0	0	1
Kapisthala	0	0	1
Kasyapa	4	3	14
Katyayana	4	1	0
Kaundinya	4	4	3
Kausika	13	5	13
Kautsa	5	0	1
Krsnatreya	0	0	4
Kumaraharita	0	0	2
Lohita	0	0	2
Mangalya	0	1	0
Maudgalya	0	1	2
Parasara	12	4	8
Sandilya	2	3	2
Savarna	9	0	0
Udavahi	0	1	0
Uluka	0	0	1
Vaisvamitra	0	1	1
Vatsa	19	10	20
Vashistha	1	1	2
Vishnuvrddha	2	0	0

REFERENCE: Singh U. (1994) *Kings, Brahmanas and Temples in Orissa: An Epigraphic Sudy AD 300-1147. Munshiram Manoharlal Publ, New Delhi, p333-334.*

Table S36. **Vangor conversion rate under Portuguese Inquisition**.

	VANGOD	FAMILY	CONVERTS: 1560-1590
Raia	2	KAMAT	
Raia	10	KAMAT	
Raia	12	KAMAT	
Kudtari	1	KAMAT	
Kudtari	3	KAMAT	
Kudtari	4	KAMAT	
Kudtari	5	KAMAT	
Kudtari	7	KAMAT	
Kudtari	8	KAMAT	
Kudtari	9	KAMAT	
Kudtari	10	KAMAT	
Kudtari	12	KAMAT	
Kudtari	14	KAMAT	
Kudtari	15	KAMAT	
Kudtari	16	KAMAT	
Kudtari	17	KAMAT	
Lotli	6	NAYAK	
Lotli	9	NAYAK	
Lotli	11	NAYAK	
Lotli	13	NAYAK	Antonio Barreto, Antonio Quadros, Manuel Alvares
Lotli	14	NAYAK	
Raia	6	NAYAK	
Raia	9	NAYAK	
Kudtari	11	NAYAK	
Lotli	2	PAI	Antonio da Costa
Lotli	5	PAI	Joao de Vargas (Valladares?)
Lotli	7	PAI	Pantaleao de Sa
Lotli	8	PAI	Jeronimo Mascarenhas
Lotli	10	PAI	Pedro Colaco
Lotli	12	PAI	
Raia	1	PAI	
Raia	3	PAI	
Raia	4	PAI	Francisco de Lima
Raia	5	PAI	Pero Parras
Raia	8	PAI	Antonio da Costa
Raia	11	PAI	Antonio Moniz
Raia	13	PAI	
Kudtari	2	PAI	
Kudtari	6	PAI	
Kudtari	13	PAI	
Kudtari	18	PAI	

**Loutolim**	**3**	**SHENAI**	
**Loutolim**	**4**	**SHENAI**	
**Loutolim**	**1**	**KINI**	
**Raia**	**7**	**MALO**	

REFERENCE: Gracias JBA. (1934) Os Primeiros Cristaos em Salcete. *Oriente Portugues*. 6: 306-337.

Table S37. **Presumed godfathers of converts prior to 1590**. By custom, early converts took the first and last name of their Portuguese godfather, usually a priest (in Salcete, Jesuit). As the colonial community was small, as Jesuit records show, the names were unique for that period.

Conversion Date	Father (Vangor)	Convert	Presumed godfather	Visited Goa
Pre-1586	Ram Pai (2)	Antonio da Costa	P. Antonio Da Costa (left Lisbon aboard the S. Filipe 1 April 1555); Reitor de Sao Paulo 23 Jun 1567; d. 1578;	1567-1578
Pre-1586	Narna Pai (5)	Joao de Vargas / Valadares	Unknown	unknown
Pre-1586	Santo Pai (7)	Pantaliao de Sa	D. Pantaliao de Sa (Goa 1577-) sea captain, royal family, left Lisbon 1548 for Lourenco Marques, then Ormuz in 1551; Goa in 1577)	1577-1586
1576-1586	Nar Pai (8)	Jeronymo Mascarenhas	D. Jeronymo Mascarenhas (Goa 1583-1593), captain of fleet, veteran of Bacaim, Ormuz, Malabar, Sangicer, Indonesia); made bequest to Jesuits for construction of Bom Jesus Cathedral;	1576-1593
1560-1571	Anta Pai (10)	Pero Colaco	P. Pero Collaco; Portuguese b 1531-2; Second vicar of Rachol (1562 to 1565); later Orlim (1568) and died in the siege of Chaul (1571);	1562-1566
Post-1586	Dassu Pai (12)	Baltezar de Araujo?	Baltezar de Araujo, Procurador do Colegio de Sao Paulo 1567;	1565-1577
1560-1577	Rama Nayak (13)	Antonio Quadros	P. Antonio de Quadros (Capitania 1 April 1555); 1528-1572; nat Santarem son of Andre de Quadros; joined SJ at 18; blue blood; 14 yrs Provincial (1558-1572);	1560-1572
1560-1577	Rama Nayak (13)	Manuel Alvares	P. Manoel Alvares; (1527-1571; joined SJ 1549) Jewish convert; celebrated painter arr. Goa 1562-64; painted "Conversion of St. Paul" over main altar of St. Paul's church completed 1572; survived shipwreck of S. Paulo ship 1561 off Sumatra, DI vol 91 p.94;	1564-1571
Pre-1586	Rama Nayak (13)	Antonio Barreto	D. Antonio Moniz Barreto, experienced & successful conquistador; Gov. Gen. Goa 9 Dec 1573 - Sept 1576);	1573-1576

REFERENCES:

1. Monumenta historica Societatis Iesu. Vols. 83-96: Documenta Indica (1561-1588). Wicki, Josef, ed.
2. Gracias JBA. (1934) Os Primeiros Cristaos em Salcete. *Oriente Portugues*. 6: 306-337.

Table S38. **Ordination of catholic priests of Lotli town (18th-20th century)**

Year	Name	Father	Mother	Family
1758	Antonio Francisco Xavier da Costa	Miguel da Costa	Rosa de Noronha	COSTA
1758	Jose Manuel Gomes	Agostinho Gomes	Senhorinha Menezes	GOMES
1758	Joao Antonio Francisco Gracias	Andre Gracias	Leonora Vas	GRACIAS
1758	Rafael Gracias	Jose Gracias	Sebastiana Fernandes	GRACIAS
1758	Caetano Xavier de Mendonca	Custodio de Mendonca	Rosa Carvalho	MENDONCA
1759	Jacome Tolentino Alvares	Albano Alvares	Emerciana Pimenta	ALVARES
1759	Antonio Baltazar Rodrigues Faria	Manoel de Faria	Antonia Rodrigues	FARIA
1759	Joao Pereira	Luis Pereira	Brigida Torrado	PEREIRA
1759	Cosme Damiao de Figueiredo e Pereira	Joao de Figueiredo e Pereira	Ana Barreto	PEREIRA
1759	Pedro Paulo Sobrinho	Custodio Sobrinho	Querobina Baptista	SOBRINHO
1760	Pedro Paulo Alvares	Caetano Alvares	Rufina de Araujo	ALVARES
1760	Jose Mascarenhas	Pedro Mascarenhas	Joana Pereira	MASCARENHAS
1760	Jose Caetano de Miranda	Luis de Miranda	Isabel Mazarelo	MIRANDA
1760	Antonio Jose de Noronha	Caetano de Noronha	Benedita Colaco	NORONHA
1760	Manuel Salvador de Rego	Caetano do Rego	Marcelina de Andrade	REGO
1765	Micael Arcanjo de Araujo	Lourenco de Araujo	Bibiana de Melo	ARAUJO
1765	Bartolomeu Mascarenhas	Antonio Joao Mascarenhas	Rosalia de Rebelo	MASCARENHAS
1765	Francisco Manuel Mascarenhas	Damiao Mascarenhas	Bernarda de Quadros	MASCARENHAS
1765	Antonio Joao Sobrinho	Custodio Sobrinho	Querobina Baptista	SOBRINHO
1766	Francisco Carvalho	Sebastiao Carvalho	Florenca Dias	CARVALHO
1766	Manuel Rosario de Quadros	Salvador Antonio Quadros	Maria De Miranda	QUADROS
1767	Pedro Roque Estanislau Barreto	Teotonio Barreto	Jacinta de Figueiredo	BARRETO
1768	Antonio Xavier de Miranda	Joao de Miranda	Maria de Nazare	MIRANDA
1769	Vicente Manoel Barreto	Bartolomeu Barreto	Isabel Fernandes	BARRETO
1769	Rosario Deodato de Figueiredo	Sebastiao de Figueiredo	Agada de Figueiredo	FIGUEIREDO
1769	Jose Joaquim de Figueiredo	Caetano de Figueiredo	Petornila Pereira	FIGUEIREDO
1769	Pedro Menezes	Aleixo de Menezes	Rita Celestina de Menezes	MENEZES
1770	Diogo Vicente Monteiro	Pedro Paulo Monteiro	Maria de Jesus	MONTEIRO
1771	Jose Manuel Alvares	Sebastiao Alvares	Maria Conceicao Barreto	ALVARES
1771	Antonio Manoel da Cruz	Domingos da Cruz	Angela da Cruz	CRUZ
1771	Antonio Paulo Pereira	Lauriano Pereira	Peregrina Rodrigues	PEREIRA
1772	Jose Sebastiao Bernardo Alvares	Antonio Manoel Alvares	Pedrinha Goncalves	ALVARES

1772	Jose Francisco de Figueiredo	Aleixo Nicolau de Figueiredo	Joana de Noronha	FIGUEIREDO
1772	Filipe Pedro Gracias	Miguel Gracias	Ursula Cardoso	GRACIAS
1772	Joao Baptista de Miranda	Bonifacio de Miranda	Isabel da Cunha	MIRANDA
1772	Jose Lourenco Valadares	Mateus Valadares	Benedita Tavares	VALADARES
1773	Paulo Rosario Gracias	Domingos Felipe Gracias	Micaela Vas	GRACIAS
1780	Jose Conceicao de Miranda	Caetano de Miranda	Vitoria de Figueiredo	MIRANDA
1786	Jose Custodio de Jesus Maria Clemente	Urbano Caetano Clemente	Arcangela Rebelo	CLEMENTE
1786	Miguel do Rosario Santiago de Miranda	Inocencio de Miranda	Rita Vas	MIRANDA
1789	Antonio Filipe Santiago Pereira	Joao Baptista Pereira	Ana Francisco de Menezes	PEREIRA
1795	Antonio Caetano de Noronha	Jose Benedito de Noronha	Ana Vitoria de Moreira	NORONHA
1796	Francisco Mariano de Miranda	Bonifacio de Miranda	Isabel de Cunha	MIRANDA
1797	Luis Caetano Gabriel de Figueiredo	Antonio Nicolau de Figueiredo	Maria Esperanca de Melo	FIGUEIREDO
1799	Joao Camilo da Costa	Custodio Manoel da Costa	Sebastiana Rufina Pereira	COSTA
1799	Caetano Francisco Pereira	Jose Vicente Pereira	Petornila Coutinho	PEREIRA
1801	Caetano Rosario da Costa	Jose Salvador da Costa	Ursula de Araujo	COSTA
1802	Caetano Gabriel Arcanjo Mascarenhas	Pedro Paulo do Rosario Mascarenhas	Rosa Conceicao Mascarenhas	MASCARENHAS
1802	Antonio Bras de Rosario e Miranda	Nicolau Miranda	Conceicao Rebelo	MIRANDA
1802	Caetano Joao Pereira	Antonio Pereira	Ana Alvares	PEREIRA
1803	Eusebio Micael Mascarenhas	Pascoal do Rosario Mascarenhas	Sebastiana Pereira	MASCARENHAS
1804	Pedro Custodio Camilo Barreto	Luis Barreto	Rita Maria Colaco	BARRETO
1804	Jose Francisco do Rosario Clemente	Manoel Francisco Clemente	Ana Maria Viegas	CLEMENTE
1804	Luis Caetano de Figueiredo	Vicente Joao de Figueiredo	Ana Secunda Xavier da Costa	FIGUEIREDO
1807	Luis Caetano da Silva	Lourenco da Silva	Aurora Cristina Colaco	SILVA
1808	Justo Sebastiao Alvares	Antonio Nicolau Alvares	Teresa Maria Moreira	ALVARES
1808	Custodio Goncalo de Araujo	Caetano Jose de Araujo	Angela Esperanca Gracias	ARAUJO
1808	Antonio Salvador de Araujo	Menino Peregrino de Araujo	Josefa Maria Lume de Araujo	ARAUJO
1809	Joaquim Francisco Piedade de Figueiredo	Vicente Joao de Figueiredo	Ana Secunda Xavier da Costa	FIGUEIREDO
1810	Inacio Tolentino Camilo Clemente	Gaspar Clemente	Rita Pacheco	CLEMENTE
1815	Vicente Mariano Barreto	Deodato Francisco Barreto	Margarida Xavier Pereira	BARRETO

1815	Roque Constancio da Costa	Roque da Costa	Frutuosa Viegas	COSTA
1815	Urbano Caetano Clemente Faria	Manoel Xavier de Faria	Rita Esperanca Parras	FARIA
1818	Jose Manoel Gomes	Antonio Nicolau Gomes	Angela Teresa Benta Correa	GOMES
1818	Miguel Filipe de Quadros	Antonio Joao de Quadros	Maria Benta da Piedade de Souza	QUADROS
1821	Pedro Alcantara de Quadros	Antonio Joao de Quadros	Maria Benta da Piedade de Souza	QUADROS
1822	Gabriel Caetano do Rosario Mascarenhas	Damiao Vicente Mascarenhas	Rita Eugenia Alvares	MASCARENHAS
1822	Joao Baptista Maximo do Rosario Miranda	Antonio Francisco de Miranda	Rosa Ana Xavier da Costa	MIRANDA
1832	Vitor Mariano Benedito de Araujo	Joaquim Lourenco de Araujo	Maria Josefa de Brito	ARAUJO
1832	Sebastiao Inacio Caetano de Figueiredo	Cosme Damiao Mariano de Figueiredo	Maria Conceicao de Figueiredo	FIGUEIREDO
1832	Joao Monteiro	Custodio Manoel Monteiro	Joaquina Aurora Gracias	MONTEIRO
1834	Joao Otono da Conceicao e Araujo	Frederico Guilherme de Souza e Araujo	Maria Josefa Manoel Piedade da Costa	ARAUJO
1845	Agostinho Monica de Conceicao e Noronha	Miguel Jose de Noronha	Anastasia Barbosa	NORONHA
1849	Domingos Caetano Barreto	Adeodato Francisco Barreto	Margarida Xavier Pereira	BARRETO
1853	Jose Tomas Casimiro de Anunciacao Barreto	Domingos Caetano Barreto	Maria Estela Aurora Colaco	BARRETO
1853	Filipe Neri da Costa	Francisco Xavier da Costa	Micaela Esperanca da Costa	COSTA
1853	Joaquim Francisco Fortunato do Rosario Figueiredo	Antonio Camilo da Conceicao Figueiredo	Rosalia Piedade Barreto	FIGUEIREDO
1853	Gabriel Vitorino Bernardo Felipe Neri Figueiredo	Francisco Caetano Figueiredo	Micael Arcangela Pinto	FIGUEIREDO
1853	Joao Jose Augusto Xavier de Miranda	Custodio do Rosario Piedade Miranda	Ana Rita Especiosa Estibeiro	MIRANDA
1853	Vicente Salvador dos Milagres Monteiro	Nicolau Xavier Monteiro	Emerenciana Severina Faleiro	MONTEIRO
1853	Joaquim Jose Piedade Sobrinho	Sebastiao Diniz Sobrinho	Maria Esperanca Figueiredo	SOBRINHO
1863	Caetano Jose de Figueiredo	Jose Joaquim da Piedade Figueiredo	Ana Florinda Rosalia de Menezes	FIGUEIREDO
1864	Basilio Joao Cosme Piedade Barreto	Domingos Caetano Barreto	Maria Estela Aurora Colaco	BARRETO
1864	Daniel Caetano da Costa	Sebastiao dos Remedios e Costa	Ana Rita Arcangela de Figueiredo	COSTA
1864	Joao Otono de Sa	Joao Gabriel de Sa	Marcelina Vas	DE SA
1864	Joaquim Mariano S. Ana de Figueiredo	Jose Joaquim de Figueiredo	Maria Felicidade do Rosario da Graca	FIGUEIREDO
1864	Evaristo Mariano de Figueiredo	Jose Joaquim da Piedade Figueiredo	Ana Florinda Rosalia de Menezes	FIGUEIREDO

1865	Custodio do Rosario Caetano Barreto	Luis Antonio Barreto	Rita Severina Faleiro	BARRETO
1866	Antonio Constancio Sebastiao Salvador da Costa	Joaquim Vicente Miguel da Costa	Eufregina Felicidade Monteiro	COSTA
1866	Francisco Salvador de Figueiredo	Jose Joaquim da Piedade Figueiredo	Ana Florinda Rosalia de Menezes	FIGUEIREDO
1866	Tomas Aquino Mariano de Figueiredo	Antonio Gregorio dos Santos Figueiredo	Rofina Quiteria da Silva	FIGUEIREDO
1866	Joao Salvador Gracias	Antonio Caetano Gracias	Ana Rita de Souza	GRACIAS
1866	Antonio Visitacao de Ave Maria Monteiro	Francisco Xavier da Piedade Monteiro	Prudencia Teresa Rosario Gracias	MONTEIRO
1867	Roque Hermenegildo Salvador Barreto	Rosario Constancio S. Ana Barreto	Maria Avelina Rozina Monteiro	BARRETO
1867	Romualdo Valeriano de Menezes	Joaquim Esmeraldo de Menezes	Eufrasia Monica Ascencao Mascarenhas	MENEZES
1876	Antonio Inacio Carvalho	Jose Francisco Carvalho	Rosa Deodata Ferrao	CARVALHO
1879	Jose Manoel Constancio Gomes	Luis Xavier Salvador Gomes	Maria Esmeralda Rosalia Felicidade Colaco	GOMES
1879	Joaquim dos Milagres da Conceicao Pimenta	Manoel Salvador Pimenta	Ana Maria Pimenta	PIMENTA
1879	Pedro Alcantara Lamartine Quadros	Miguel Antonio Quadros	Ana Quiteria Piedade Xavier	QUADROS
1879	Manoel Salvador Nascimento Vas	(not recorded)	(not recorded)	VAS
1881	Matias Camilo Barreto	Deodato Francisco Antonio Barreto	Orfelina da Silva	BARRETO
1881	Roque Redualdo das Neves e Costa	Antonio Jose Anunciacao da Costa	Rosa da Piedade Menezes	COSTA
1881	Custodio Salvador Domiciano Miranda	Antonio Francisco Jose Piedade Miranda	Rita Maria Conceicao Correa	MIRANDA
1881	Jose Agostinho Monteiro	Vicente Custodio Monteiro	Maria Francisca da Cruz	MONTEIRO
1881	Cipriano Manoel Mariano da Sacra Familia Sobrinho	Jose Caetano Sobrinho	Ana Rita Aleluia Soares	SOBRINHO
1883	Rafael Xavier Roque Jose de Figueiredo	Salvador do Rosario e Figueiredo	Maria Expectacao de Araujo	FIGUEIREDO
1883	Jose Salustiano Boaventura Monteiro	Francisco Xavier da Piedade Monteiro	Prudencia Teresa Rosario Gracias	MONTEIRO
1883	Pedro Floriano da Piedade Vas	Domingos Santana Vas	Ana Patrocinia Sobrinho	VAS
1885	Roque da Piedade Gracias	Joaquim Sebastiao Rosario da Piedade Gracias	Maria Lucia da Santa Trinidade Borges	GRACIAS
1885	Joao Francisco Sebastiao Xavier da Silva	Salvador da Piedade da Silva	Maria Barbina Colaco	SILVA
1886	Inacio Francisco de Santana Costa	Antonio Jose Anunciacao da Costa	Rosa da Piedade Menezes	COSTA

1886	Jose Manoel Nicolau Nepomuceno Francisco Xavier do Rosario Milagres Gomes	Jose Vicente Gomes	Maria Idultrides Pereira	GOMES
1887	Jose das Augustias Sacramento Francisco Xavier S. Ana Barreto	Adeodato Francisco Antonio Barreto	Julia Orfelina da Silva	BARRETO
1887	Pascoal S. Ana Roque do Rosario Colaco	Manoel Xavier Colaco	Maria Piedade de Quadros	COLACO
1887	Custodio do Rosario Quadros	Jose Rosario dos Remedios Quadros	Maria Josefa Lume de Arcanjo	QUADROS
1888	Custodio Goncalo de Araujo	Vitor Maximo Micael de Araujo	Ana Rita Pereira	ARAUJO
1888	Jose Francisco Eduardo Navarro Lobo	Joaquim Santana Constancio Isac Lobo	Rita Aleluia Monteiro	LOBO
1894	Sebastiao Francisco Anselmo Damasceno de Araujo	Roque de Santana Lourenco Araujo	Quiteria Piedade Pereira	ARAUJO
1894	Joao Vicente Francisco Xavier de Menezes	Camilo Vicente de Exaltacao Menezes	Escolastica Severina do Rosario Cardoso	MENEZES
1897	Henrique Caetano Vitor de Figueiredo	Jose Antonio de Figueiredo	Matilde Sebastiana da Costa	FIGUEIREDO
1897	Jeronimo Pantaleao de Miranda	Manoel Salvador Joaquim Santana Sebastiao de Miranda	Ana Joaquina Florencia Barreto	MIRANDA
1897	Caetano Francisco Platinho do Rosario Monteiro	Inacio Francisco Monteiro	Ana Clarina de Quadros	MONTEIRO
1897	Arcanjo Antonio do Rosario Vas	Caetano Vicente Vas	Emilia Severina Quadros	VAS
1898	Custodio da Piedade Pimenta	Remedio Baltazar Pimenta	Ana Joaquina Quiteria Mascarenhas	PIMENTA
1900	Jose Avelino Joaquim Sebastiao das Dores e Menezes	Jose Avelino Menezes	Maria Felicidade Rodrigues	MENEZES
1901	Caetano Francisco Xavier Lucio Pedro Damiao Araujo	Caetano Joao de Araujo	Maria Caetana Idalina de Santa Rita Viegas	ARAUJO
1904	Gabriel Joao Clementino Sebastiao Valadares	Joao Constancio Santana Valadares	Ana Francisca Etelvina Pimenta	VALADARES
1909	Bernardo Anacleto Joaquim de Figueiredo	Antonio Gabriel de Figueiredo	Ermelinda Parras	FIGUEIREDO
1909	Hipolito Francisco Juveniano Mascarenhas	Vicente Caetano das Angustias Mascarenhas	Rita Severina do Rosario Costa	MASCARENHAS
1909	Custodio Piedade Francisco Monteiro	Jose Xavier Monteiro	Maria Liberata Caridade Araujo	MONTEIRO
1909	Vicente Francisco Rosario da Piedade Monteiro	Esmeraldo Olegario Monteiro	Maria Leocadia da Piedade Gregolina Rebelo	MONTEIRO
1910	Joao Celestino do Rosario Piedade Figueiredo	Francisco Antonio de Figueiredo	Quiteria Faleiro	FIGUEIREDO

1910	Aleixo Santa Rita da Piedade Monteiro	Rosario Agostinho da Exaltacao Monteiro	Maria Angelica Herminia da Piedade Costa	MONTEIRO
1918	Francisco Xavier Inacio Santana Colaco	Filipe Salvador Roque Sebastiao Colaco	Maria Matildes Generosa Gracias e Colaco	COLACO
1921	Manoel Salvador dos Milagres Pimenta	Erasmo Sebastiao Pimenta	Hermenegilda Dias	PIMENTA
1922	Constancio Floriano Faria	Jose Xavier Gelasio de Anunciacao Faria	Luisa Maria Ermelinda Colaco	FARIA
1923	Emiliano Salvador Sebastiao dos Milagres Monteiro	Sebastiao Xavier Feliciano Monteiro	Ana Joaquina Dulcina Da Cunha	MONTEIRO
1924	Bismarck Salisburi Antonio Garibaldi Mascarenhas	Sebastiao Constancio Mascarenhas	Maria Clotildes Amelina Fernandes	MASCARENHAS
1925	Francisco Xavier Colaco	(not recorded)	(not recorded)	COLACO
1760	Filipe Caetano Soares	Antonio Soares	Paula de Orta	SOARES
1765	Miguel Antonio de Fonseca	Ambrosio da Fonseca	Paula Benedita Moniz	FONSECA
1804	Inacio Francisco Xavier da Fonseca	Francisco Xavier da Fonseca	Benedita Baptista	FONSECA
1847	Lopo Jose Joao dos Reis	Bernardo dos Reis	Maria Liberata Pereira	REIS
1853	Joao Vicente Avelino da Fonseca	Jose Salvador das Merces da Fonseca	Querobina Josefa dos Reis	FONSECA
1864	Caetano Salvador Roque Joaquim de Andrade	Jose Santana de Andrade	Maria Joaquina Rodrigues	ANDRADE
1865	Sebastiao Xavier Damasceno Tavares	Inacio Antonio Tavares	Damiana Francisca Pedrosa	TAVARES
1867	Filipe Manoel dos Reis	Jose Joaquim Filipe dos Reis	Maria Aurora Rodrigues	REIS
1881	Joaquim do Rosario Idalino Socorro Albano Torrado	Sebastiao Milagres Cipriano Torrado	Maria Pulqueria da Cunha	TORRADO
1881	Julio Avertano Torrado	Caetano Lourenco Torrado	Celestina Pereira	TORRADO
1883	Manoel do Rosario Jorge	Aleixo Cristovao Jorge	Maria Angelina Anunciacao Tavares	JORGE
1887	Damaso Sertorio Misquita	Remedios Benjamim Misquita	Maria Francisca Figueiredo	MISQUITA
1887	Adolfo Antonio Tavares	Jose Caetano Tavares	Maria Esperanca da Cruz	TAVARES
1904	Lopo Jose dos Reis	Jose Bernardo dos Reis	Josefina Apolonia da Costa Reis	REIS
1765	Joao de Siqueira	Caetano de Siqueira	Maria Fernandes	SIQUEIRA
1766	Pedro Paulo Baptista	Antonio Baptista	Teresa da Cruz	BAPTISTA
1772	Bernardo Jose Antonio Baptista	Roque Baptista	Rita Barbosa	BAPTISTA
1780	Rosario Salvador Cardoso	Custodio Cardoso	Esperanca de Noronha	CARDOSO
1798	Inacio Francisco Fernandes	Francisco Xavier Fernandes	Violante Maria do Rosario	FERNANDES
1801	Camilo Caetano Piedade Baptista	Roque Baptista	Cristina Dias das Neves	BAPTISTA

1806	Miguel Baptista	Luis Antonio Baptista	Feliciana Pascoela Mascarenhas	BAPTISTA
1824	Joaquim Vicente Salvador Fernandes	Filipe Neri Fernandes	Rita da Piedade Rodrigues	FERNANDES
1826	Sebastiao Xavier Cardoso	Mariano Camilo Cardoso	Severina Gomes	CARDOSO
1853	Antonio Caetano da Piedade Baptista	Francisco Pinto Simao Bras Baptista	Maria Conceicao Rodrigues	BAPTISTA
1866	Sebastiao Piedade das Merces Faleiro	Eusebio Nicolau Faleiro	Esperanca Barreto	FALEIRO
1867	Antonio Manuel de Santa Cruz Fernandes	Francisco Xavier Fernandes	Maria Esperanca Xavier	FERNANDES
1879	Jose Custodio Fernandes	Francisco Xavier Fernandes	Maria Esperanca Xavier	FERNANDES
1881	Caetano Sebastiao Cardoso	Jose Xavier Cardoso	Maria Anunciacao Vas	CARDOSO
1883	Pedro paulo da Piedade dos Remedios Mota	Narciso Santa Ana Mota	Caetana Maria Purificacao	MOTA
1888	Francisco Xavier Faleiro	Jose Sebastiao Luciano Faleiro	Rita Piedade Amatildes Faleiro	FALEIRO
1891	Nicolau Sebastiao Joaquim Faleiro	Caetano Salvador do Rosario Faleiro	Ana Maria Monteiro	FALEIRO
1900	Sebastiao Caetano Piedade Cota	Sebastiao Caetano da Piedade Cota	Ana Maria Leonora Amanda Corina Abranches	COTA
1905	Ildefonso da Piedade Cota	Sebastiao Caetano da Piedade Cota	Ana Maria Leonora Amanda Corina Abranches	COTA
1906	Francisco Xavier Fernandes	Joaquim Salvador Pascoal Fernandes	Ismenia Aurora Esperanca Barreto	FERNANDES

REFERENCE: Vaz FX (1925) *Monumenta Goana Eclesiastica*. Tipografia Braganca & Co, Nova Goa.

Table S39. **Vangor extinction rate.**

Extinction of Brahmin Vangods in Salcete (1848)*

	Kms (distance)	Original	Extant
Kushasthali	0	24	7
Quelossim	4	8	3
Nagoa	6	14	7
Verna	7	48	29
Margao	16	28	22
Benaulim	21	12	10
Raia	21	14	12
Lotli	25	14	13
Kudtari	26	27	25

Vangod Survival Rate (1848-present)

VANGODS (#)	**TOWN**	**1848***	**1930-50****
LPK (7)	LOTLI	85.7	71.4
KAMAT (11)	KUDTARI	100.0	100

*1848 data from: Xavier PN. (1907) Bosquejo Historico das Communidades. Tipografia Rangel, Bastorá.

**1930-1950 data from baptismal records

***2012 data from electoral rolls

Table S40. **Economic elites in South Asia**.

Community	Census 1891	Census 2001	Pct. Executives*	Ratio**
**All Hindus**	77.80	80.50	87.97	1.09
**Brahmin**	5.55	5.16	36.26	7.02
**Khatri ^**	0.51	0.54	16.28	30.04
**Kayastha**	0.84	0.66	9.59	14.63
**Lingayat**	0.25	0.80	0.55	0.68
**Muslims**	12.86	13.40	1.12	0.08
**Christians**	0.86	2.30	5.40	2.35
**Jains**	0.53	0.40	1.79	4.48
**Parsis**	0.03	0.01	2.34	379.8

*Combined: (Public & Private Sector) Chairman, President, Managing Director, General Manager, Director, Executive Director (Goyal, 1990)
**Ratio of actual incidence to expected incidence
^ Census combines Arora and Khatri communities

Reference: Goyal S (1990) Social background of Indian corporate executives. In M.S.A. Rao & Francine Frankel (eds.) *Dominance and state power in modern India: Decline of a social order* (pp.535-544). Delhi, India: Oxford University Press.

File S41. **Western cultural corridor in ancient times.**

A.

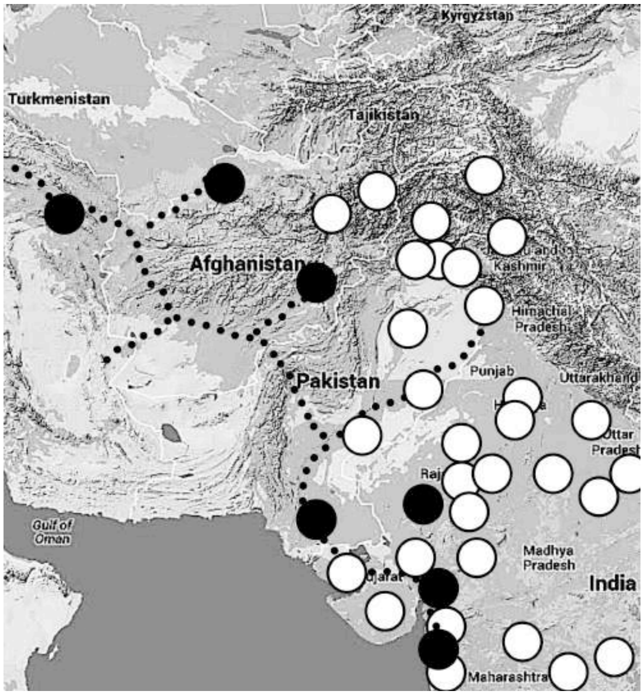

B.

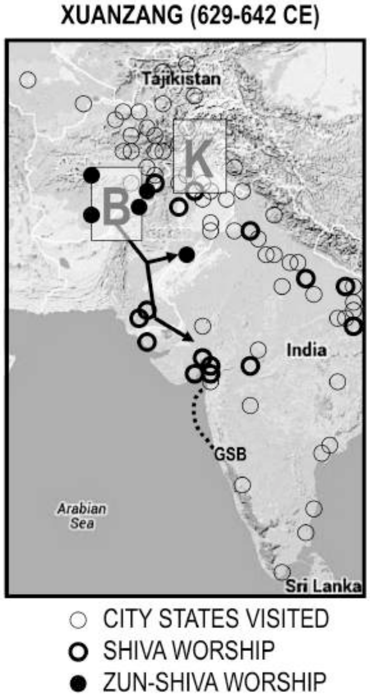

A. The first millennium CE Skanda Purana lists 72 territories (vibhedas) of India. Their location is shown by circles in the illustration. Among these are several territories with names ending with the suffix –ka (black circles), an orthodox Avestan and Vedic ethnonym designating "foreign-ness" [Ciancaglini C, 2012; Supplemental File S24]. The territory surrounding Barygaza, the major port of Gujarat at the beginning of the Common Era, was known as Ariaka [Schoff, 1912]. B. In his religious census, the 7th century Chinese traveller Xuanzang documented Shiva worship and Zun-Shiva worship primarily in the northwestern regions of the subcontinent in 629-642 CE [Rongxi L, 1996; Supplemental File S23].

Ciancaglini C. (2012) Outcomes of the Indo-Iranian Suffix *-ka- in Old Persian and Avestan. In: Dariosh Studies II. Persepolis and its settlements: territorial system and ideology in the achaemenid state. Basello GP and Rossi AV (eds) p.91-100. Naples.

Rongxi L. (tr.) (1996) The Great Tang Dynasty Record of the Western Regions. Numata Center Buddhist Translation, Berkeley, CA.

Schoff WH. (1912) *The Periplus of the Erythraean Sea: Travel and Trade in the Indian Ocean by a Merchant of the First Century. Munshiram Manoharlal Publ., 2001.*

File S42: **Western cultural corridor: Mitra-Varuna tradition of Kaundinya gotra.**

A. B.

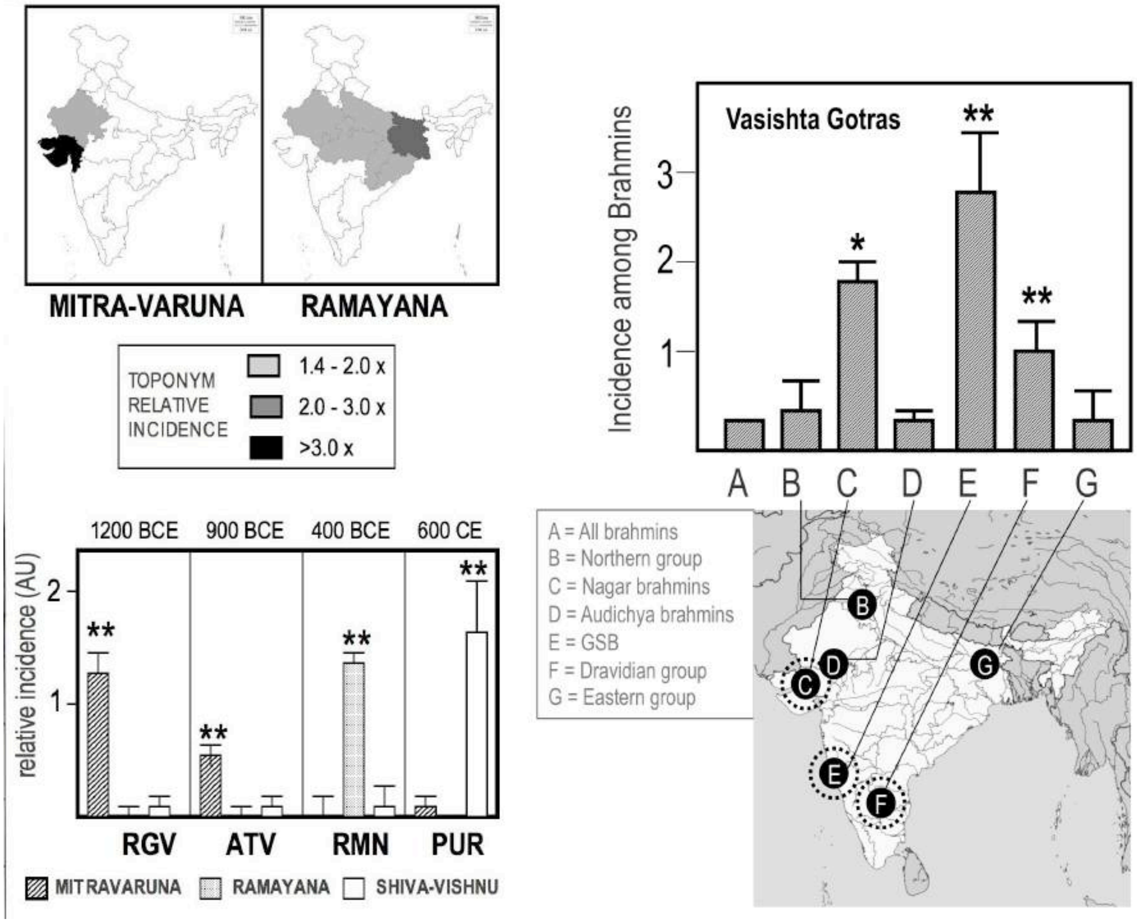

A. 2nd millenium toponymic footprint: Relative incidence of toponyms (Suppl. File S17) was corrected for the geographic incidence of deity or epic hero-related toponyms in the 20 major states and expressed as a ratio to the expected national incidence. The relative incidence of the same word-stems (plus Shiva and Vishnu) was tabulated in ancient texts for the period from 1200 BCE to 600 CE (lower panel; see Suppl. File S21 for raw data). RGV=Rigveda; ATV=Atharvaveda; RMN=Ramayana; PUR=Puranas. Toponyms of Mitra, an Iranic and early Vedic sun god, are strongly associated with the Gujarat entry point to the subcontinent, whereas toponyms of the Ramayana epic tradition (attested ca. 400 BCE) appear associated with OCP-CH domains. As the Iranic god Mitra fell out of favor with the orthodox literary establishment in India during the first millenium BCE based on relative incidence of word stems in ancient texts (lower panel) the geographic distribution of Mitra toponyms suggests a timing of settlement prior to the first millennium BCE.

B. Relative incidence of Vasishta group of gotras among geographically dispersed Brahmin communities, expressed as a multiple of the national average. Each bar is an average of brides and grooms from a matrimonial web site (Suppl. File S25). Patronage of Iranic tribes by the sage Vasishta (a central identity in a Mitra-Varuna tradition to which the LPK community's Kaundinya gotra belongs) is attested in the major ancient Indian epics, the Mahabharata and Ramayana. The above illustration shows that the westerly incidence of Vasishta gotras remains significantly ($p<0.05$) above the national average.

File S43: Saurashtran centers of worship and Arab invasions in the 8th century CE.

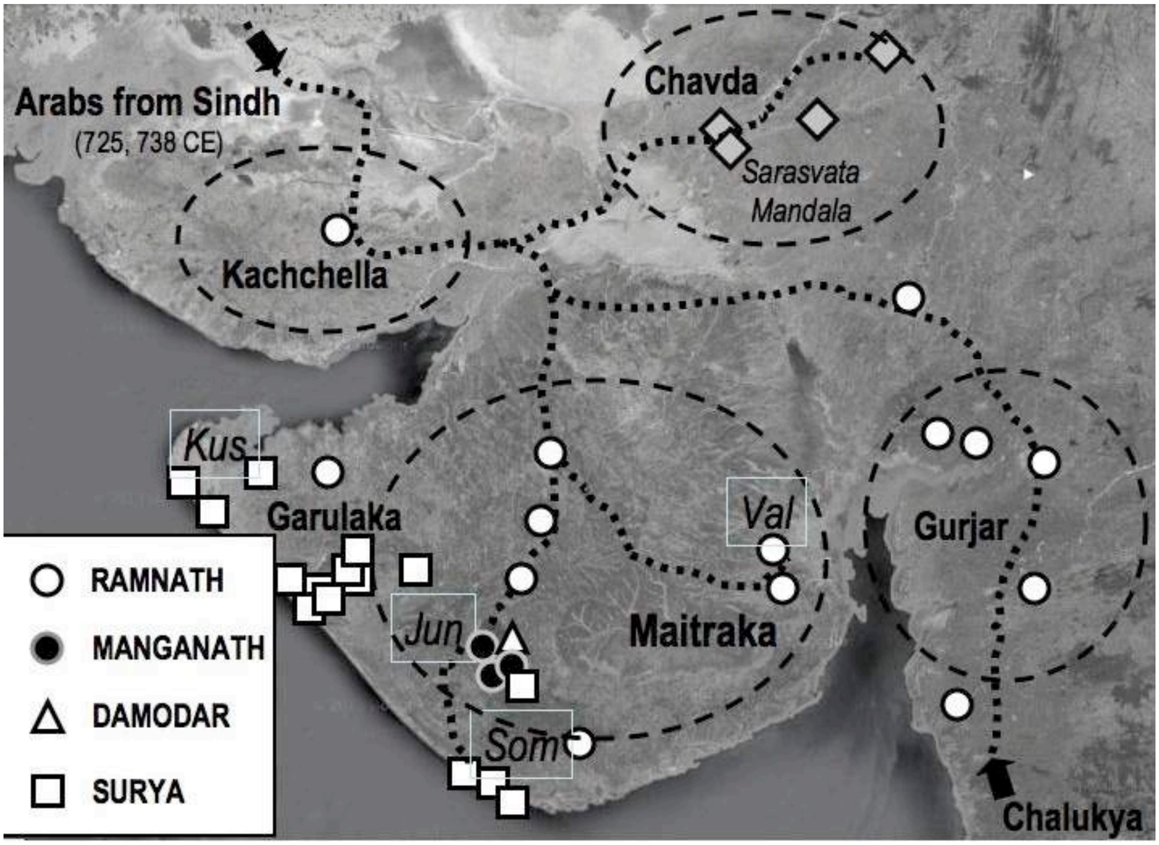

Places of worship in the putative medieval origin of Brahmin migrants to Salcete are shown by white circles and squares. With the exception of a few temples of later vintage in and around Goa, the only known places of worship dedicated to Ramnath (Lotli) and Manganath (Kushasthali) north of Maharashtra are in Saurashtra. These two male clan deities were installed in the earliest Brahmin settlements of 8th century CE Salcete. [Supplemental Files S27, S28, S29, S30, S31]. The presumed route of armed land invasions of the subcontinent by Muslim Arabs (notably in 725 and 738 CE, while Al-Junayd was emir of Sindh) is shown by the dotted line, along with kingdoms (dashed ovals) mentioned by Arab chroniclers. The up-arrow shows where the Arabs were routed by the Chalukya Pulakesi in 738-739 CE, according to the detailed account in the Navsari Plates [reference 1, below]. The invasions destroyed Vallabhi, the Maitraka capital, the most likely location of employment for Brahmins who emigrated south in 740 CE, almost certainly embarking from the port of Somnath-Patan. Chavda Gurjars (Chardo) may have migrated south at a later date (diamond symbols show location of early Chavda settlements). Kus=Kushasthali/Dwarka; Jun=Junagadh; Som=Somnath-Patan; Val=Vallabhi;

Reference 1: "Early Chalukyas of Gujarat", June 14, 2011 issue.
http://inscriptions.whatisindia.com/kalachuri-chedi/kalachuri-chedi-part1/earlychalukyasofgujarat.html
